# Supplementary material for: Genomic analyses of weedy rice reveal historical connections between European and northwestern Chinese rice
Source: Plant Commun. 2026 Mar 10;7(8):101792. doi: 10.1016/j.xplc.2026.101792 (PMC13477037; doi:10.1016/j.xplc.2026.101792)
Supplement: Supplementary file 1 [file mmc1.pdf]

Plant Communications, Volume *7*

Supplemental information

Genomic analyses of weedy rice reveal historical connections between European and northwestern Chinese rice

Yifan Zhong, Wuyue Chen, Shuai Xu, Qijun Zhang, Yixuan Wang, Xiaohui Zhang, Ju Huang, Sihai Yang, and Long Wang

Supplemental Materials

Supplemental Note

Supplemental Note 1 | Evaluating the influence of latitude on allelic effects in flowering-time field trails

Supplemental Note 2 | Assessing the geographic distribution of northwest-derived flowering alleles in public 3K rice genome database

Supplemental Note 3 | Assessing the impact of modern breeding on weedy rice populations

Supplemental Note 4 | Evaluation of the representativeness of weedy rice sampling in northwestern China

Supplemental Figure

Supplemental Figure 1 | Weedy rice phenotypes (awn and seed coat) of partially collected samples.

Supplemental Figure 2 | Phylogenetic tree of 582 rice samples based on genome-wide SNPs with sample IDs.

Supplemental Figure 3 | Primer design and experimental identification of $Hd1^{EH}$ and $Hd4^{NW}$ alleles in rice.

Supplemental Figure 4 | Functional validation of the flowering-time effects of $Hd1^{EH}$ and the deletion-type *Hd4*.

Supplemental Figure 5 | Phylogenetic tree based on SNPs in *Hd1* and its 1k bp promoter region.

Supplemental Figure 6 | Conserved genomic regions between northwestern Chinese weedy rice and European rice identified based on SNP difference ratios.

Supplemental Figure 7 | Geographic distribution of photoperiod genotypes of northwestern origin.

Supplemental Figure 8 | Phylogenies based on SNPs within the gene body and 3-kb upstream and downstream flanking regions of six other photoperiod-related genes.

Supplemental Figure 9 | Schematic diagram of *japonica* weedy rice dispersal and effective

population size history.

Supplemental Figure 10 | Pairwise genetic differentiation (F_{ST}) between the three weedy rice populations.

Supplemental Figure 11 | Minimal impact of modern breeding on local weedy rice populations.

Supplemental Figure 12 | Phylogenetic analysis of the *Rc* gene provides additional evidence for the northwestern Chinese origin of European rice.

Supplemental Figure 13 | Sampling locations of the samples collected from northwestern China in this study.

Supplemental Table

Supplemental Table 1 | IBD segment lengths between Wr_Italy20 and European cultivars

Supplemental Table 2 | IBD segment lengths between European weedy rice individuals and modern European modern cultivars

Supplemental Table 3 | Information of primers in this study.

Supplemental Table 4 | Rice accessions in the 3K rice genome database (3kRG) that carry photoperiod-related alleles found in northwest China.

Supplemental Data

Supplemental Data 1 | List of 23 weedy rice accessions collected and used in this study.

Supplemental Data 2 | List of 559 published rice accessions representing modern cultivars, landraces, and weedy rice.

Supplemental Data 3 | Conserved genomic regions between European rice and northwestern weedy rice.

Supplemental Data 4 | Summary of *Hd1* haplotypes and their distribution across *japonica* rice groups.

Supplemental Data 5 | Summary of *Hd4* haplotypes and their distribution across *japonica* rice groups.

Supplemental Data 6 | Summary of *Hd2* haplotypes and their distribution across *japonica* rice groups.

Supplemental Data 7 | Representative samples used to predict population history.

Supplemental Data 8 | Genotypes of major heading date genes in *japonica* rice samples used in this study.

Supplemental Data 9 | Statistical analyses in this study.

Supplemental references

Supplemental Notes

Supplemental Note 1 | Evaluating the influence of latitude on allelic effects in flowering-time field trails

Because Nanjing is located at a lower latitude than northwestern China and most European rice-growing regions, differences in day-length could influence the absolute heading dates of photoperiod-responsive genes such as *Hd1* and *Hd4*. To assess the robustness of the allelic effects detected in our Nanjing field trials, we further examined multi-site European flowering-time data from Gómez-Ariza et al., (2015), which include five field locations (Italy, France, Greece, Portugal, and Spain) and three years of observations from Italy. For both genes, we restricted comparisons to accessions sharing similar genotypes at other major flowering-time loci to minimize background effects.

For *Hd1*, our Nanjing field trials showed that weedy rice carrying the $Hd1^{EH}$ alleles consistently headed approximately 10.6 days earlier than those carrying the functional $Hd1^{NIP}$ haplotype (Supplemental Figure 4A). Under European field conditions, accessions with matched genotypes at other flowering-time genes (non-functional *Hd5*, functional *Hd2* and *Hd4*) carrying the $Hd1^{EH}$ allele (n = 6) flowered ~6.8 days earlier than those with the functional *Hd1* allele (n = 4) (linear model, $P = 3.591\times 10^{-5}$, Supplemental Figure 4C).

For *Hd4*, our Nanjing trials showed that accessions carrying the $Hd4^{NW}$ alleles flowered approximately 16.0 days earlier than those with the functional $Hd4^{NIP}$ haplotypes (Figure 2G). Consistently, under Europe conditions and matched allelic states at *Hd1* (non-functional), *Hd2* (functional) and *Hd5* (functional), accessions carrying $Hd4^{NW}$ (n = 4) flowered ~9.1 days earlier than those carrying the functional *Hd4* allele (n = 58) (linear model, P = 0.00499, Supplemental Figure 4D).

Collectively, although the absolute magnitudes of flowering-time differences vary between low- and high-latitude environments, likely reflecting photoperiod variation, the direction and relative strength of allelic effects are highly consistent across environments. These results demonstrate that the functional impacts of $Hd1^{EH}$ and $Hd4^{NW}$ are stable across

environments and genetic backgrounds, supporting their roles in photoperiod adaptation in both northwestern Chinese and European rice populations.

Supplemental Note 2 | Assessing the geographic distribution of northwest-derived flowering alleles in public 3K rice genome database

To further evaluate the geographic distribution of key flowering-time alleles, we analyzed variation at the *Hd1*, *Hd2*, and *Hd4* loci using the 3K rice genome database (3kRG). Despite limitations in geographic resolution and the absence of large structural variants, we identified consistent high-latitude enrichment patterns (Supplemental Table 4). The *Hd1*^{EH} haplotype was detected in 39 accessions, predominantly from Europe, while *Hd2*^{NW} was found in 23 accessions, over half of which were distributed in Europe and Georgia (Supplemental Table 4). For *Hd4*, 18 accessions carried the inferred *Hd4*^{NW} deletion haplotype, again largely restricted to high-latitude regions (Supplemental Table 4). Importantly, the full *Hd1*^{EH}-*Hd2*^{NW}-*Hd4*^{NW} allele combination was detected only in a limited number of accessions from Europe, Georgia, and China, a distribution consistent with historical Silk Road regions (Supplemental Table 4). These results independently reinforce the close genetic relationship between European rice and Chinese rice lineages.

Supplemental Note 3 | Assessing the impact of modern breeding on weedy rice populations

Extensive germplasm exchange between Europe and China during modern rice breeding, together with the dynamic emergence of weedy rice in Europe due to changes in cultivation practices, could potentially confound signals of geographic origin. To evaluate these possibilities, we examined accessions with known histories of germplasm exchange and assessed their influence on weedy rice populations.

First, to evaluate the impact of modern breeding activities, we used the European cultivar "*Balila*", a parental line widely used in breeding programs in northeastern China, as a representative example. In our phylogenetic analyses, *Balila* (Inter_Italy_36) clustered within Clade 1, whereas northeastern cultivars clustered in Clade 3, indicating that its

incorporation into Chinese breeding did not distrupt large-scale phylogenetic structure (Supplemental Figure 2). IBD analyses further showed that *Balila* shares extensive IBD segments with European cultivars but only short tracts with northeastern or northwestern Chinese cultivars (Supplemental Figure 11A). In contrast, weedy rice from all three regions showed only minimal IBD sharing with *Balila* (Supplemental Figure 11A), demonstrating that the phylogenetic clustering of weedy rice remains geographically stable and largely unaffected by modern breeding. Moreover, if intensive modern breeding had obscured ancestral genealogies, modern cultivars would be genetically closer to local weedy rice than landraces. However, in northwest China, weedy rice exhibits lower F_{ST} with local landraces (which experienced minimal modern breeding) but higher F_{ST} with modern cultivars introduced from North China, northeastern China, and Japan (Supplemental Figure 11B). This pattern is inconsistent with extensive recent genetic replacement and argues against the erosion of ancestral signals.

Second, consider the impact of recent cultivar escape, if the European weedy rice had arisen predominantly from recent escapes (post-1980s), they would be expected to cluster closely with cultivars widely planted during that period. To test this, we included representative Italian cultivars spanning the 1980s to the present, such as Sant'Andrea (Inter_Italy_03, planted on 10,823 ha in 1985), Smeraldo (Inter_Italy_15, planted on 4,353 ha in 1985), Rubino (Inter_Italy_25, planted on 913 ha in 1980), Gladio (Inter_Italy_34, developed and planted in the 1990s), Augusto (Inter_Italy_29, still cultivated today), and Loto (Inter_Italy_13, still cultivated today) (Data source: https://openknowledge.fao.org/server/api/core/bitstreams/392ee95b-6852-42cd-a8ec-1a8d8ad6eaf8/content/y4347e0z.htm#TopOfPage). These cultivars are all clustered within Clade 1 (Figure 1C, Supplemental Figure 2). In contrast, most Italian weedy rice samples fall outside this clade (Figure 1C, Supplemental Figure 2), indicating that their de-domestication predates the post-1980s expansion. This finding aligns with prior evidence of continuous historical escape events contributing to European weedy rice populations instead of a recent major outbreak (Grimm et al., 2013).

Together, these results show that neither cultivation practices nor modern varietal turnover has substantially altered the deeper population structure of weedy rice. Ancestral genealogical signals remain intact, supporting the robustness of origin inferences based on genomic data.

Supplemental Note 4 | Evaluation of the representativeness of weedy rice sampling in northwestern China

Genomic inferences are inherently sensitive to sampling design and geographic coverage. To minimize potential bias, our study was intentionally structured to expand sampling in regions historically underrepresented in rice evolutionary studies, particularly northwestern China, which has a long history of rice cultivation but a relatively limited record of modern breeding.

First, earlier investigations into the origin of European rice primarily emphasized northeast China and East Asia, while rice populations from northwest China were largely overlooked. Given the long-standing role of northwest China as a major corridor connecting East and West, we explicitly included both cultivated and weedy rice from this region. Because European rice cultivation is dominated by *japonica* (~90%), we also concentrated on *japonica*-growing regions globally, ensuring balanced representation across relevant ancestral source populations.

Second, sampling bias is more problematic when large areas are sparsely sampled. In contrast, rice cultivation in northwest China is geographically constrained to highly localized and river-valley systems (Ouyang et al., 2023). Our sampling spans all major rice-growing areas across Gansu, Ningxia, and Xinjiang, with substantial geographic distances among sites (Supplemental Figure 13). This spatial coverage greatly reduces the risk that our findings are driven by localized genetic clusters and supports the representativeness of our dataset for the region as a whole.

Third, the extensive arid deserts separating rice-growing regions in northwest China

severely limit natural seed dispersal, making long-distance gene flow nearly entirely human mediated. The high genomic similarity and shared flowering-time allelic combinations observed across weedy rice populations in these isolated regions therefore reflect a common human-driven establishment history rather than recent natural diffusion. Crucially, this same genomic background and characteristic suite of adaptative photoperiod alleles also occurs in European weedy rice and a subset of European cultivars. Such concordance is unlikely to result from local or recent spread alone, and is most parsimoniously explained by a historical westward introduction facilitated by human movement.

Overall, the geographic breadth, ecological distinctiveness, and anthropogenic dispersal context of our northwestern China sampling support its representativeness and reinforce the robustness of our origin inferences.

Supplemental Figure

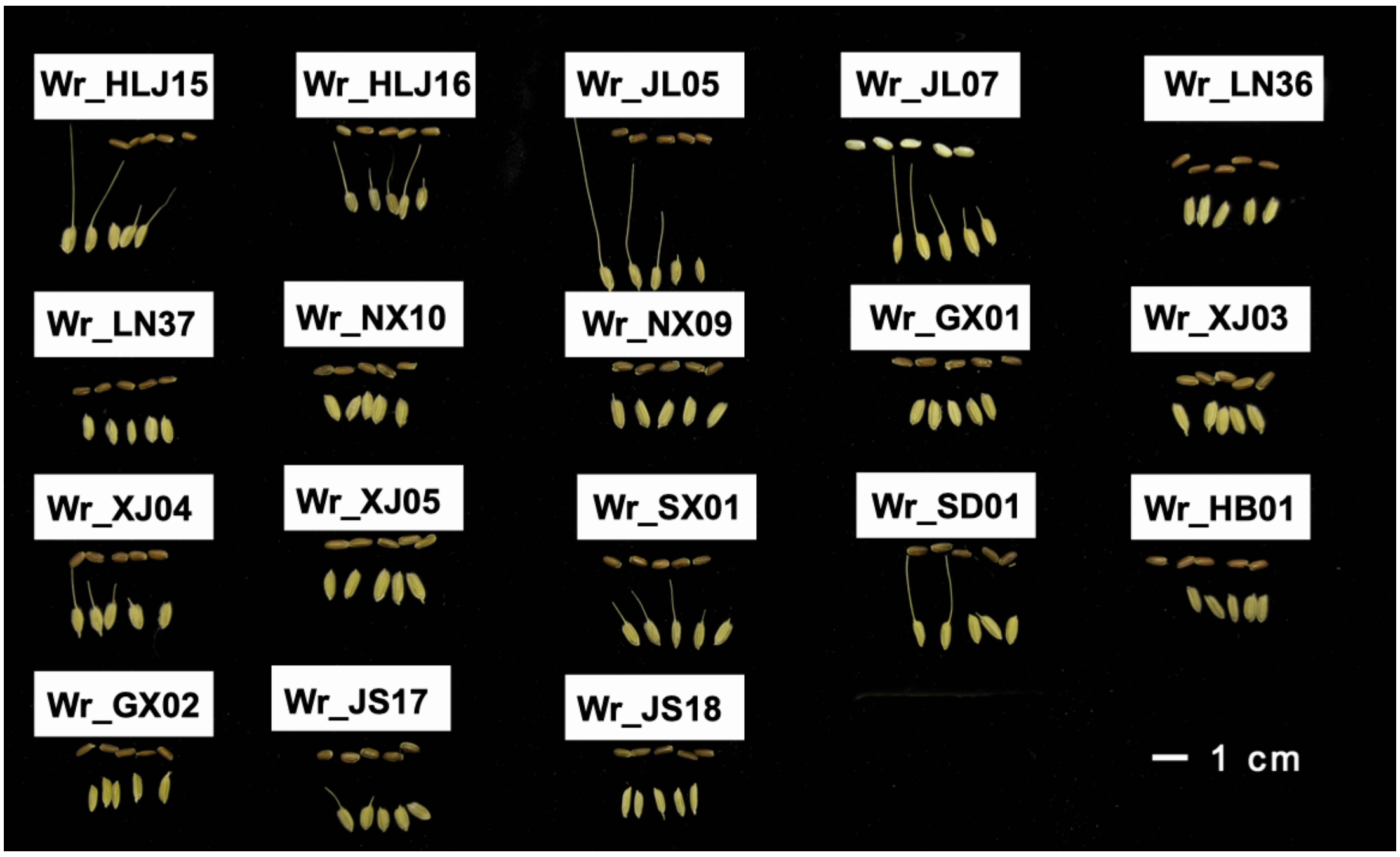

Supplemental Figure 1 | Weedy rice phenotypes (awn and seed coat) of partially collected samples. The white bar represents a length of 1 cm.

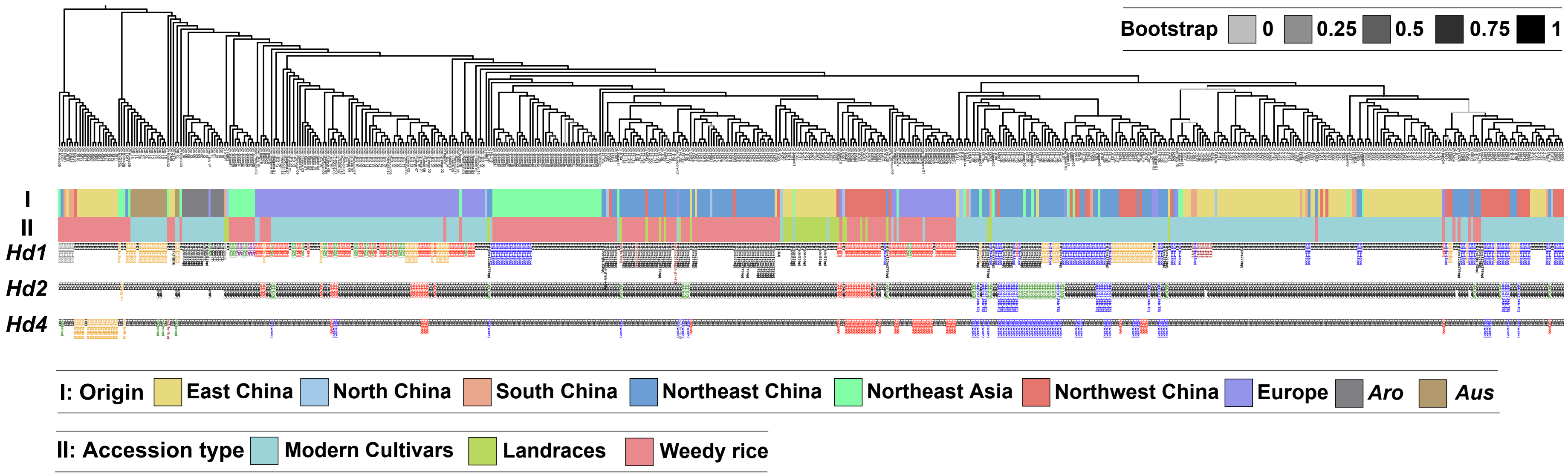

Supplemental Figure 2 | Phylogenetic tree of 582 rice samples based on genome-wide SNPs with sample IDs. Genotypes of *Hd1*, *Hd4*, and *Hd2* are annotated for each sample. The haplotype labels match the numbering used in Supplemental Data 4, Supplemental Data 5 and Supplemental Data 6. Different haplotypes are labeled using different font colors, with NW-originated haplotypes all shown in red. Annotation bars beneath the phylogenetic tree indicate geographic origin (I) and sample classification (II), using the same color scheme as in Figure 1C.

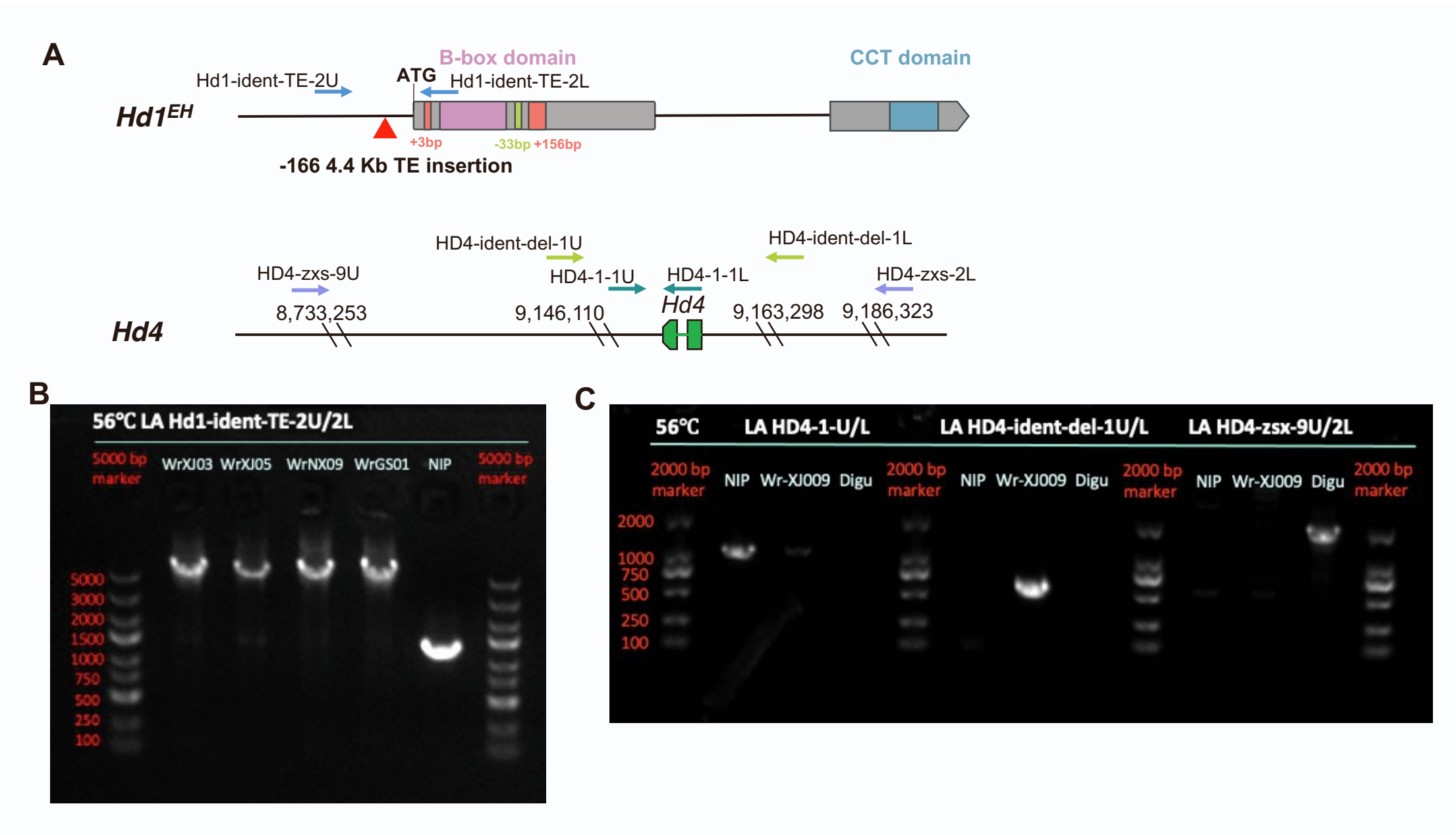

Supplemental Figure 3 | Primer design and experimental identification of $Hd1^{EH}$ and $Hd4^{NW}$ alleles in rice. (A) Schematic diagram showing the primer design regions used for the identification of $Hd1^{EH}$ and $Hd4^{NW}$ alleles. (b-c) PCR amplification results for $Hd1^{EH}$ and $Hd4^{NW}$ alleles. **(B)** Lane 1: DNA marker (size in 5000 bp); Lane 2-4: PCR products for $Hd1^{NW}$, showing a clear band over 5000 bp; Lane 5: PCR products for $Hd1^{NIP}$, showing a clear band at approximately 1380 bp. **(C)** NIP carries an intact Hd4 sequence, while Wr-XJ009 has a 17,187-bp deletion and Digu has a 453,070-bp deletion. Three distinct primer sets were designed to amplify specific bands corresponding to each genotype.

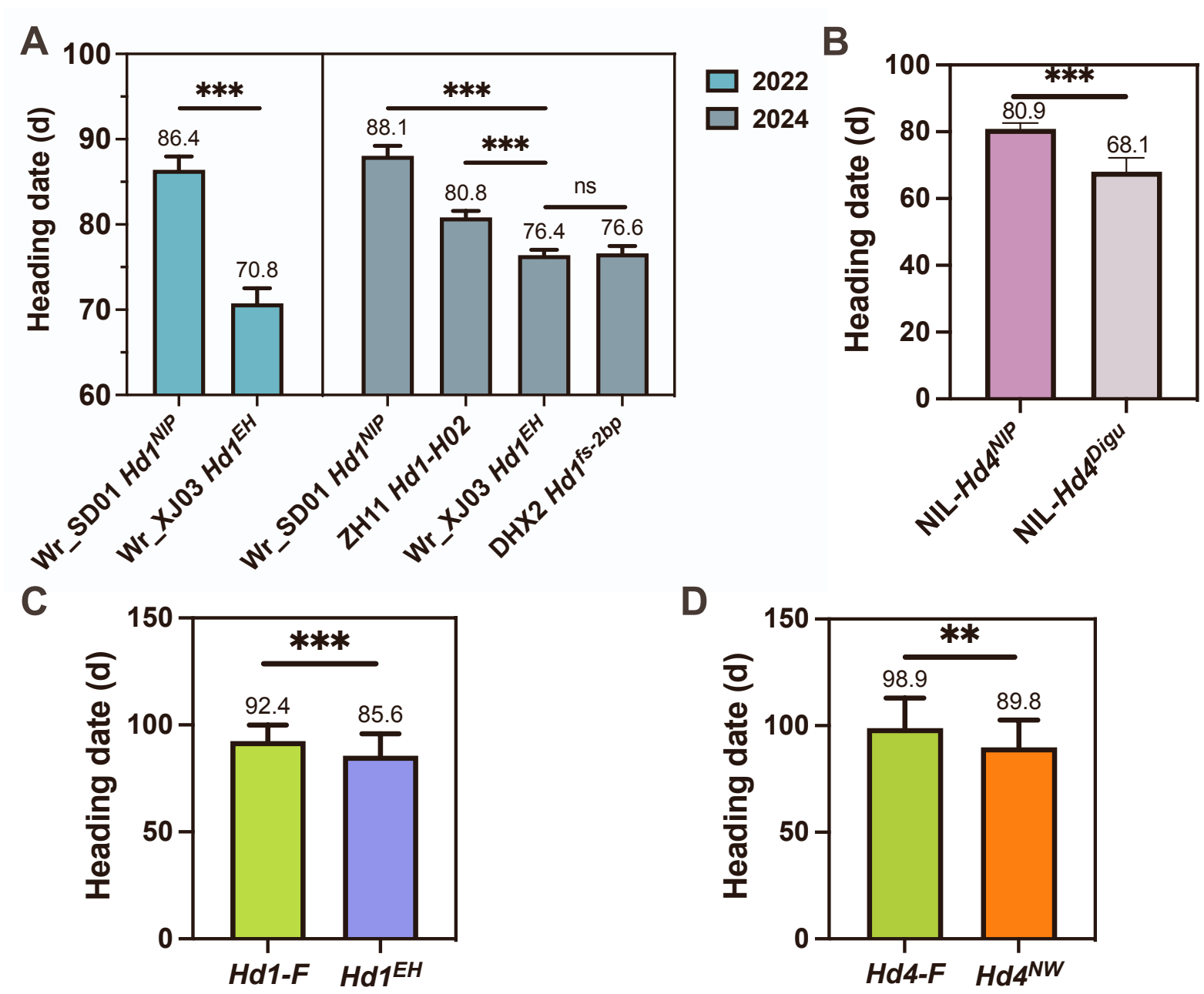

Supplemental Figure 4 | Functional validation of the flowering-time effects of *Hd1*^{EH} and the deletion-type *Hd4*. (A) Weedy rice carrying *Hd1*^{EH} flowers earlier than those with functional *Hd1* in a background with functional *Hd2, Hd4* and *Hd5*. Accessions with *Hd1*^{EH} allele flower significantly earlier than those with functional *Hd1* alleles (Student's t-test, ***P < 0.001, Data S9) but do not differ significantly from the nonfunctional *Hd1*$^{fs\text{-}2bp}$ (P = 0.462, Data S9). **(B)** Flowering-time variation associated with the deletion-type *Hd4* (*Hd4*^{Digu}) versus functional *Hd4* alleles *Hd4*^{NIP} in near isogenic lines (NILs). NILs were generated by crossing PA64s (functional *Hd4*^{NIP}) with Zhongjiazao 17 (deletion-type *Hd4*^{Digu}) and advancing progeny to the F_6 generation. From this segregating population, homozygous individuals with a uniform genetic background (functional *Hd1* and *RFT1*, and nonfunctional *Hd2* and *Hd5*) that differed only at the *Hd4* locus were selected. A significant difference in flowering time was observed between the two *Hd4* genotypes (Student's *t*-test, ***P < 0.001, Data S9). **(C)** Flowering-time differences between *Hd1*^{EH} and functional *Hd1* (*Hd1-F*) alleles in European rice germplasm. Data from Gómez-Ariza et al. (2015); effects were estimated using linear models including genotype, planting site and year as fixed factors (**P < 0.01, Data S9). **(D)** Flowering-time differences between *Hd4*^{NW} and functional *Hd4* (*Hd4-F*) in European rice germplasm. Data from Gómez-Ariza et al. (2015); effects were estimated using linear models including genotype, planting site and year as fixed factors (**P < 0.01, Data S9).

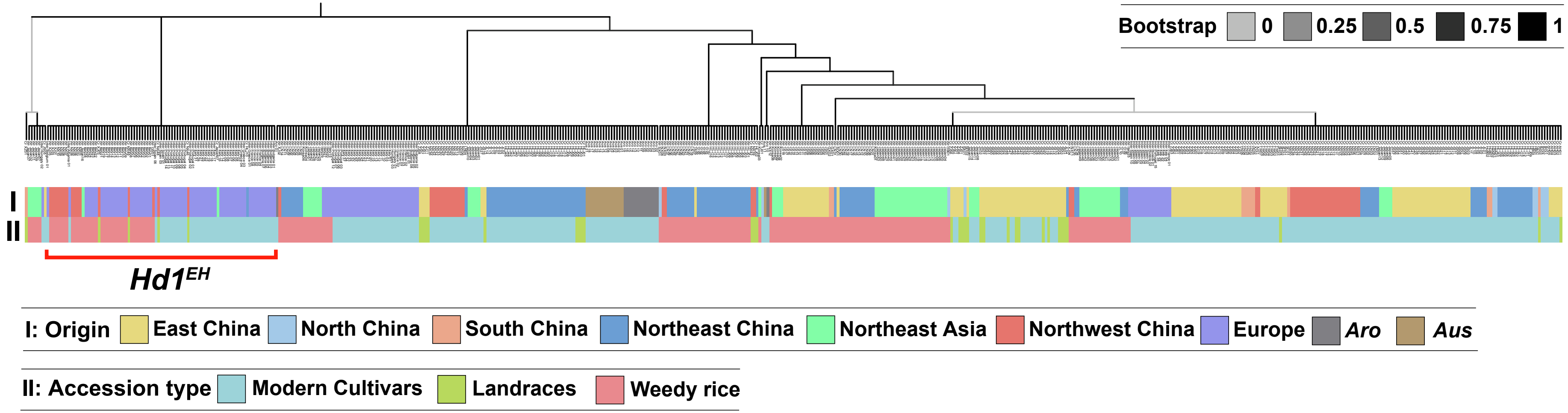

Supplemental Figure 5 | Phylogenetic tree based on SNPs in *Hd1* and its 1k bp promoter region. Individuals with the *Hd1*^{EH} haplotype are highlighted in red parentheses. Sample IDs are shown beneath the phylogeny. Annotation bars beneath the phylogenetic tree indicate geographic origin (I) and sample classification (II), using the same color scheme as in Figure 1C.

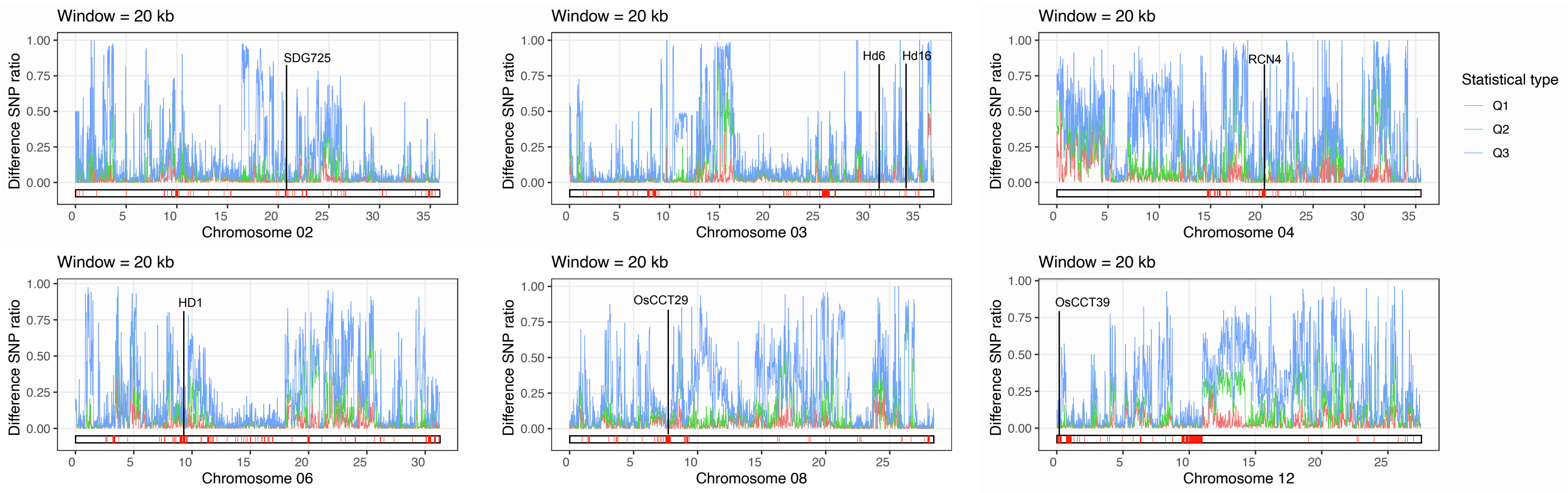

Supplemental Figure 6 | Conserved genomic regions between northwestern Chinese weedy rice and European rice identified based on SNP difference ratios. Red, green, and blue lines indicate the first quartile (Q1), median (Q2), and third quartile (Q3) of the SNP difference distribution, respectively. The red shaded regions at the bottom represent conserved genomic regions where the first quartile of the SNP differential distribution is less than 0.1.

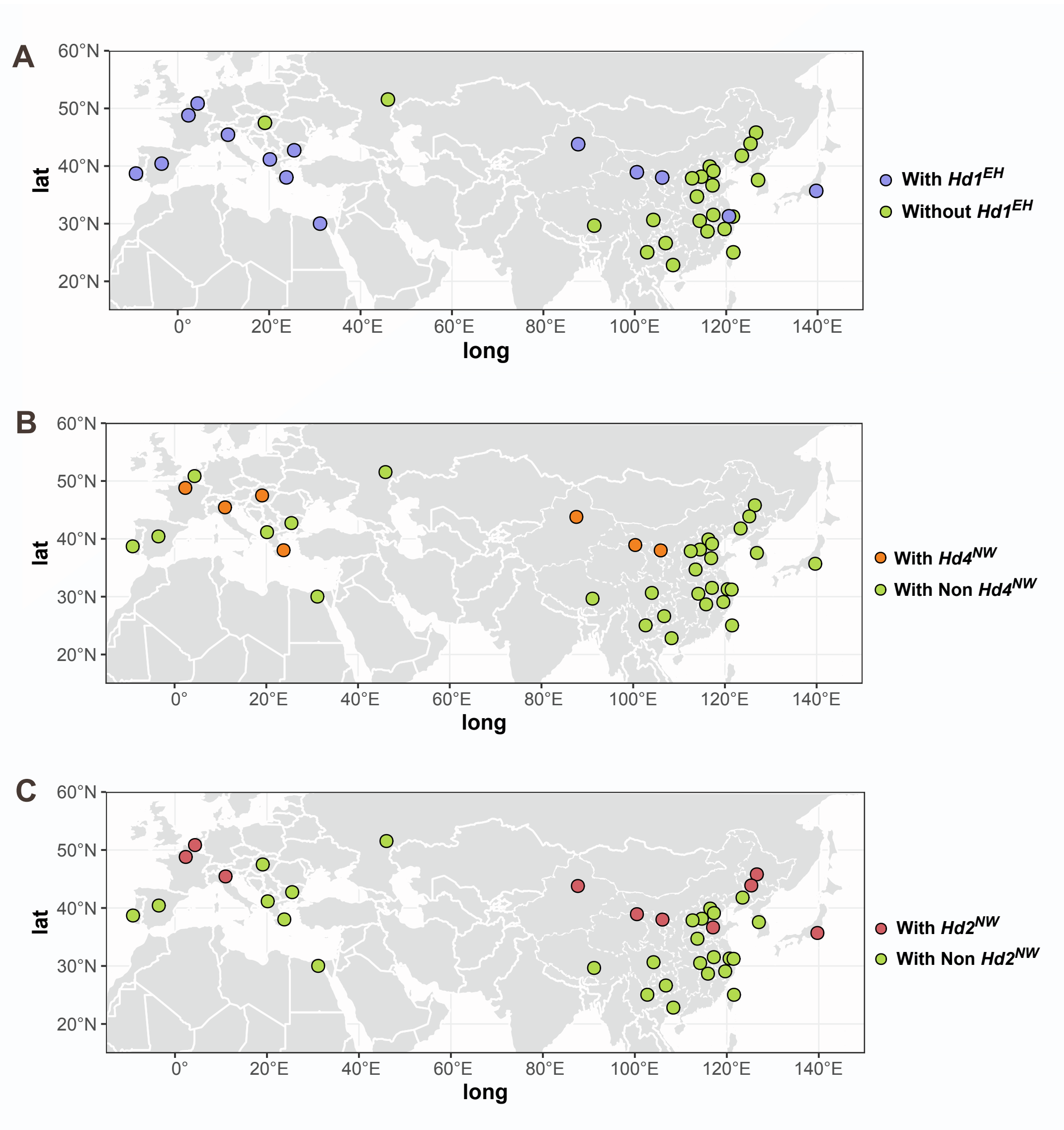

Supplemental Figure 7 | Geographic distribution of photoperiod genotypes of northwestern origin. The geographical distribution of *Hd1*^{EH} **(A)**, *Hd4*^{NW} **(B)** and *Hd2*^{NW} **(C)** in our samples across the Eurasia. Demonstrates a strong correspondence in flowering-time genotypes between northwestern Chinese and European rice populations.

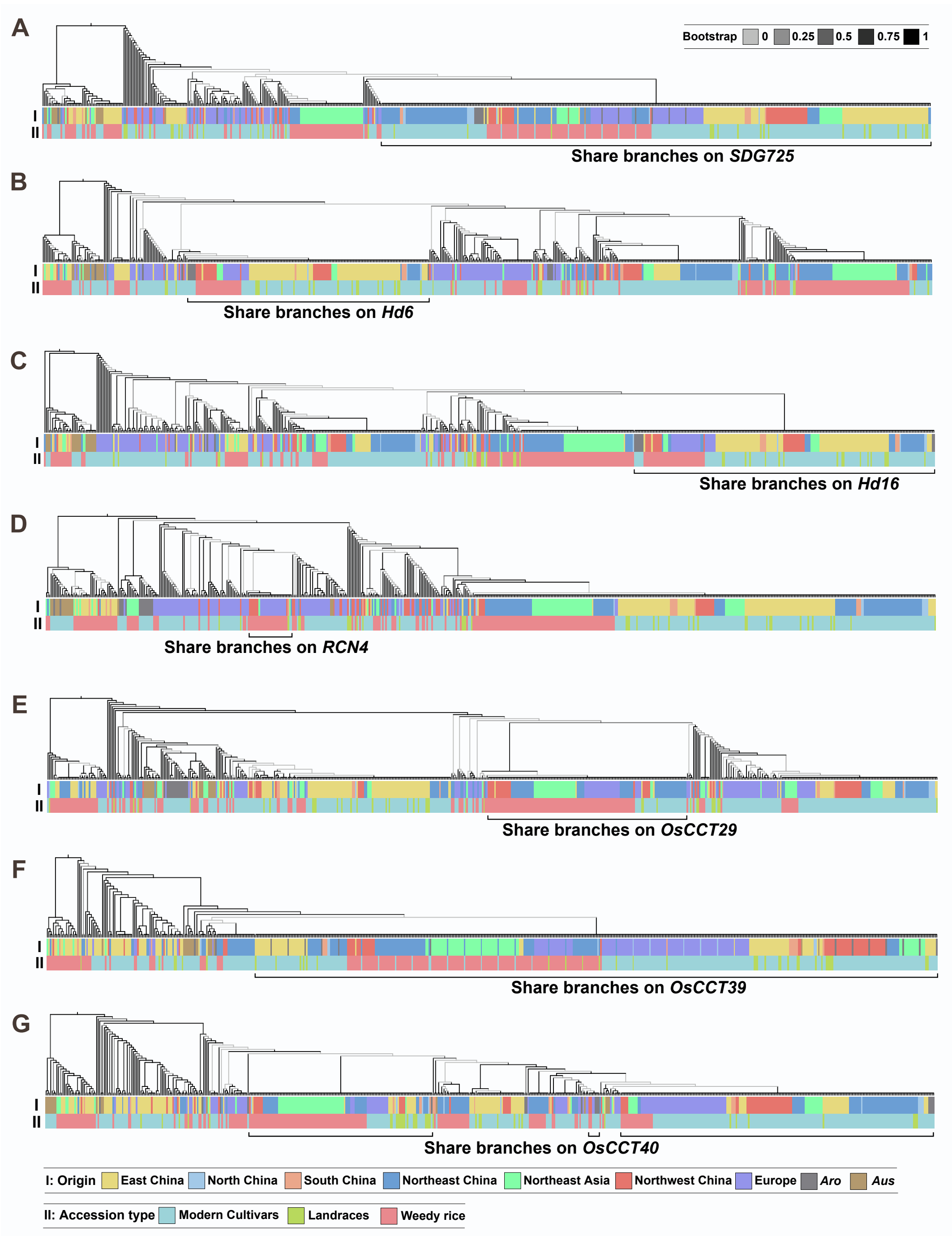

Supplemental Figure 8 | Phylogenies based on SNPs within the gene body and 3-kb upstream and downstream flanking regions of six other photoperiod-related genes. (A) *SDG725*, **(B)** *Hd6*, **(C)** *Hd16*, **(D)** *RCN4*, **(E)** *OsCCT29*, **(F)** *OsCCT39*, **(G)** *OsCCT40*. A subset of European cultivars and European weedy rice consistently cluster with northwestern Chinese weedy rice, and are highlighted with a black arc in the phylogenetic tree. Annotation bars beneath the phylogenetic tree indicate geographic origin (I) and sample classification (II), using the same color scheme as in Figure 1C.

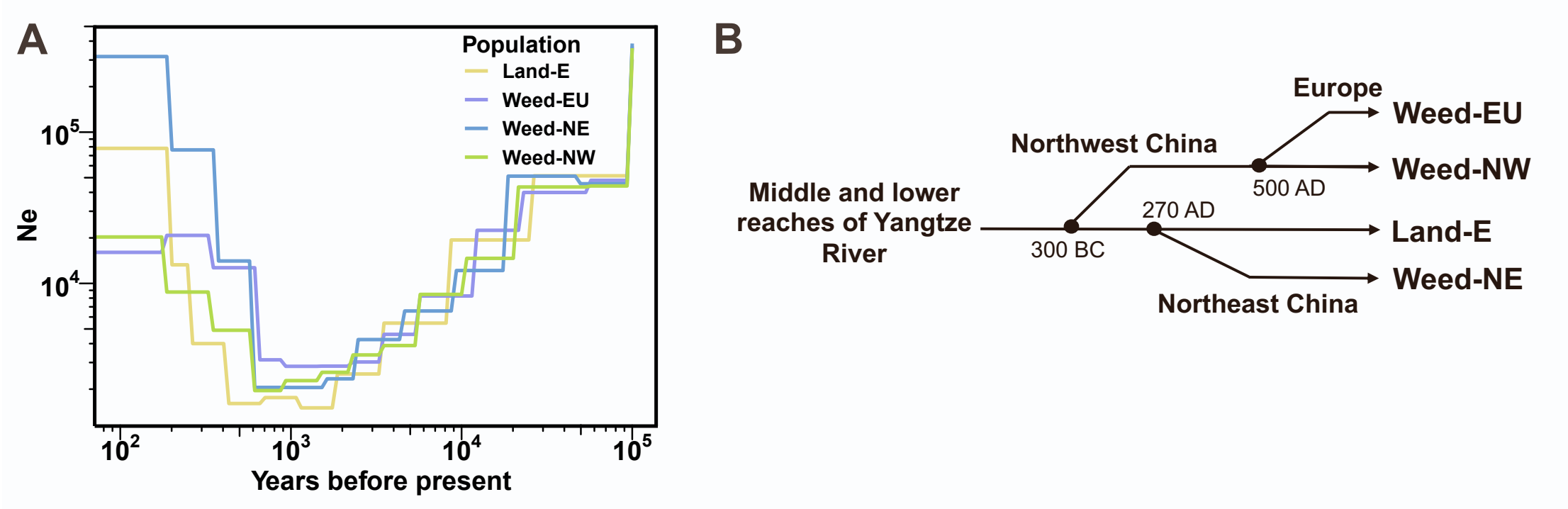

Supplemental Figure 9 | Schematic diagram of *japonica* weedy rice dispersal and effective population size history. (A) Effective population history predicted using the single population method. **(B)** Schematic diagram of the inferred dispersal timeline of *japonica* weedy rice based on effective population size history. Land-E, East Chinese landraces; Weed-NW, northwestern Chinese weedy rice; Weed-NE, northeastern Chinese weedy rice; Weed-EU, European weedy rice.

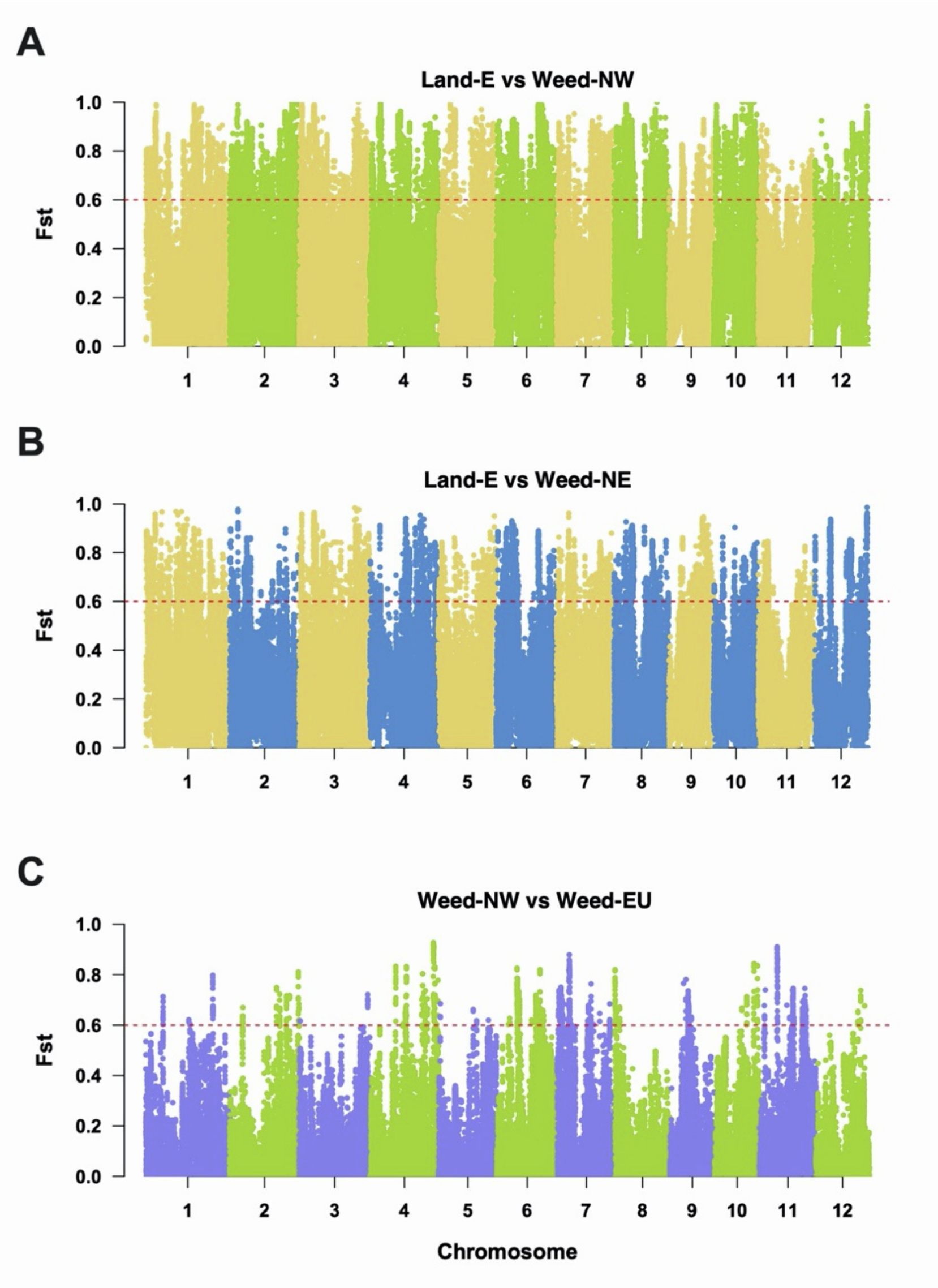

Supplemental Figure 10 | Pairwise genetic differentiation (*F*ST) between the three weedy rice populations. (A) East Chinese landraces versus northwestern Chinese weedy rice; **(B)** East Chinese landraces versus northeastern Chinese weedy rice; (C) northwestern Chinese weedy rice versus European weedy rice. Land-E, East Chinese landraces; Weed-NW, northwestern Chinese weedy rice; Weed-NE, northeastern Chinese weedy rice; Weed-EU, European weedy rice.

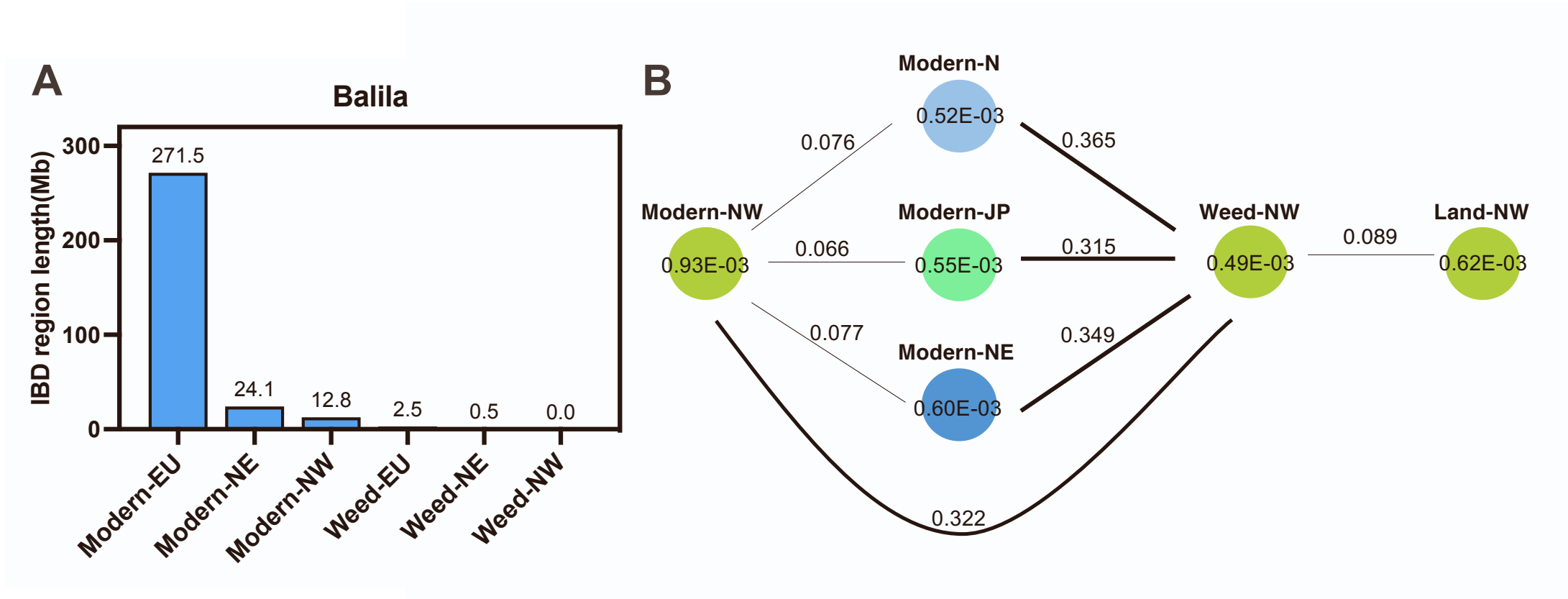

Supplemental Figure 11 | Minimal impact of modern breeding on local weedy rice populations. (A) Total length of IBD segments shared between the European cultivar *Balila* and European, northeastern, and northwestern weedy rice and modern cultivars. Numbers on the bars indicate cumulative IBD segment lengths. **(B)** Nucleotide diversity (π) and population differentiation (F_{ST}) between northwestern weedy rice, modern cultivars from various regions, and local northwestern landraces. Circle values indicate π for each population; numbers on connecting lines indicate F_{ST}. Thinner lines represent lower F_{ST} values. Modern-EU, European modern cultivars; Modern NE, northeastern Chinese modern cultivars; Modern-NW, northwestern Chinese modern cultivars; Weed-EU, European weedy rice; Weed-NE, northeastern Chinese weedy rice; Weed-NW, northwestern Chinese weedy rice; Land-NW, northwestern Chinese landraces; Modern-JP, Japanese modern cultivars.

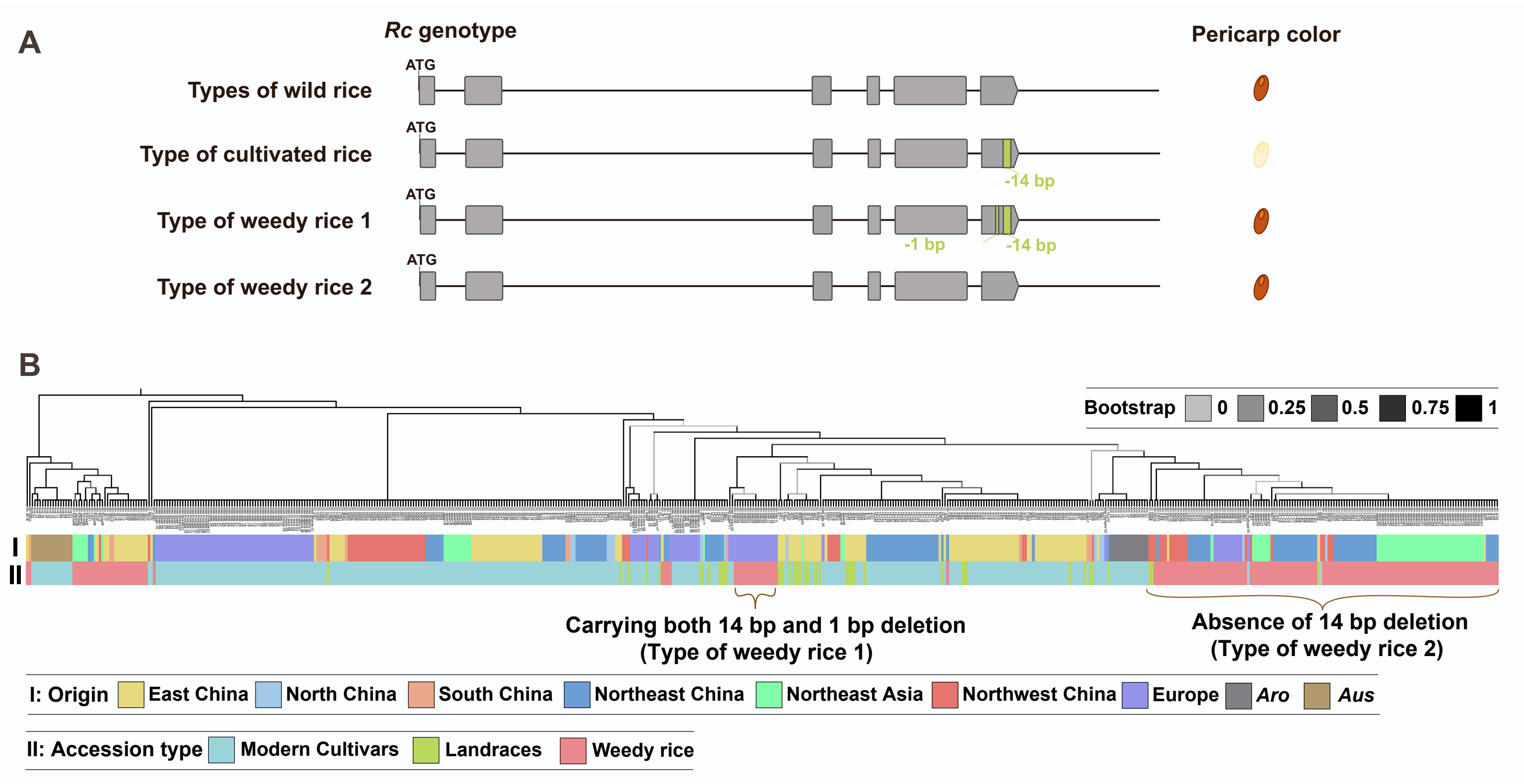

Supplemental Figure 12 | Phylogenetic analysis of the *Rc* gene provides additional evidence for the northwestern Chinese origin of European rice. (A) Representative *Rc* genotypes and corresponding pericarp color phenotypes in wild rice, cultivars, and weedy rice populations. **(B)** Phylogenetic tree constructed using SNPs from the *Rc* gene and its 3-kb upstream and downstream region, with sample IDs labeled. The two genotypes associated with red pericarp in weedy rice are marked in parentheses. Annotation bars beneath the phylogenetic tree indicate geographic origin (I) and sample classification (II), using the same color scheme as in Figure 1C.

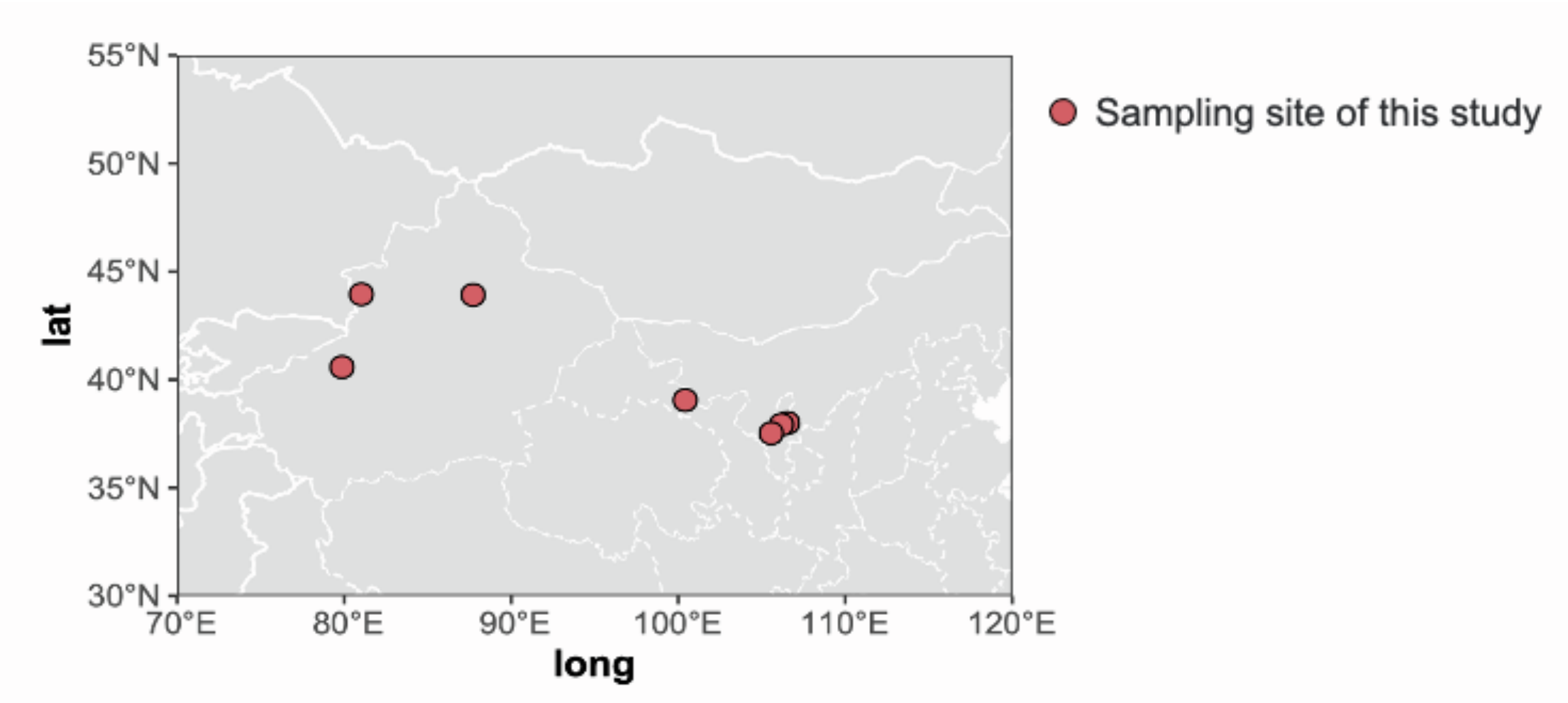

Supplemental Figure 13 | Sampling locations of the samples collected from northwestern China in this study.

Supplemental Table

Supplemental Table 1 | IBD segment lengths between Wr_Italy20 and European cultivars

Sample of European cultivars	IBD size (Mb)
Inter_Albania_01	0
Inter_Bulgaria_01	0
Inter_Bulgaria_02	0
Inter_Bulgaria_03	0.541288
Inter_Bulgaria_04	0
Inter_Bulgaria_05	0
Inter_Bulgaria_06	0
Inter_Bulgaria_07	0
Inter_Bulgaria_08	3.074247
Inter_Egypt_01	0
Inter_France_01	0
Inter_France_02	6.779831
Inter_France_03	0
Inter_France_04	0.890408
Inter_France_05	0
Inter_France_06	0
Inter_France_07	19.130954
Inter_France_08	14.591105
Inter_Greece_01	18.818832
Inter_Greece_02	69.635791
Inter_Greece_03	11.88922
Inter_Greece_04	0
Inter_Hungary_01	0
Inter_Hungary_03	0
Inter_Italy_01	0.78987
Inter_Italy_02	0
Inter_Italy_03	1.184416
Inter_Italy_04	0.702362
Inter_Italy_05	0

Inter_Italy_06	19.220945
Inter_Italy_07	1.626646
Inter_Italy_08	0.941679
Inter_Italy_09	0
Inter_Italy_10	0
Inter_Italy_11	5.636904
Inter_Italy_12	16.600106
Inter_Italy_13	0.354499
Inter_Italy_14	0
Inter_Italy_15	0
Inter_Italy_16	5.607676
Inter_Italy_17	2.053776
Inter_Italy_18	0
Inter_Italy_19	0
Inter_Italy_20	42.871146
Inter_Italy_21	5.596049
Inter_Italy_22	12.847456
Inter_Italy_23	23.386632
Inter_Italy_24	1.108355
Inter_Italy_25	0
Inter_Italy_26	71.973949
Inter_Italy_27	1.812559
Inter_Italy_28	0.468216
Inter_Italy_29	1.038515
Inter_Italy_30	2.188311
Inter_Italy_31	0
Inter_Italy_32	0
Inter_Italy_33	0.67916
Inter_Italy_34	146.413128
Inter_Italy_36	0
Inter_Italy_38	0
Inter_Portugal_01	0.971997
Inter_Portugal_02	6.073548
Inter_Portugal_03	1.037989
Inter_Portugal_04	0
Inter_Portugal_06	0

Inter_Portugal_07	0
Inter_Portugal_08	0
Inter_Portugal_09	0
Inter_Portugal_10	0.633855
Inter_Portugal_11	0
Inter_Portugal_12	0
Inter_Portugal_13	0
Inter_Portugal_14	0
Inter_Portugal_15	0
Inter_Russia_01	0
Inter_Russia_02	0
Inter_Spain_01	0
Inter_Spain_02	0
Inter_Spain_03	0
Inter_Spain_04	0
Inter_Spain_06	0
Inter_Spain_07	0
Inter_Spain_08	3.938009
Inter_Spain_09	1.373842
Inter_Spain_10	0

Supplemental Table 2 | IBD segment lengths between European weedy rice individuals and modern European modern cultivars

Sample	IBD share with CrEU (Mb)
Wr_Italy01	17.042525
Wr_Italy02	26.321698
Wr_Italy03	18.691196
Wr_Italy04	47.85537
Wr_Italy05	25.000292
Wr_Italy06	24.98659
Wr_Italy07	19.318802
Wr_Italy08	37.580425
Wr_Italy09	26.628139
Wr_Italy10	20.475191
Wr_Italy11	35.912636
Wr_Italy12	32.941376
Wr_Italy13	21.57655
Wr_Italy14	82.2356
Wr_Italy15	33.869091
Wr_Italy16	33.221424
Wr_Italy17	25.449308
Wr_Italy18	30.809438
Wr_Italy19	86.264013
Wr_Italy20	236.358609
Wr_Italy21	28.728231
Wr_Italy22	76.575907
Wr_Italy23	37.741635
Wr_Italy24	12.969275
Wr_Italy25	47.700568
Wr_Italy26	29.641857
Wr_Italy27	107.904443
Wr_Italy28	12.723567
Wr_Italy29	55.72497

Supplemental Table 3 | Information of primers in this study.

Primer name	Primer sequence	Purpose
Hd1-ident-TE-2U	ACAAACTCAATTCATCAACACGA	Identification of transposon Insertion in the *Hd1*^{EH}
Hd1-ident-TE-2L	TTTAGCTTGCACCACATGGAC	
Hd4-ident-del-1U	TTGCGGCTATAAAGATTTCCC	Identification of the *Hd4*^{NW} deletion type
Hd4-ident-del-1L	AGGAAGTATCCACAACGCAAG	
HD4-1-1U	GCGAAGCGACCTCTCACT	Identification of the *Hd4*^{NIP} functional type
HD4-1-1L	GTGCTTCTCGGTCTATTTACTT	
ZSX-9U	GCCGAAGATGACTTTATTTT	Identification of the *Hd4*^{Digu} deletion type
ZSX-2L	TGGGACCGCTTCAGTACTA	

Supplemental Table 4 | Rice accessions in the 3K rice genome database (3kRG) that carry photoperiod-related alleles found in northwest China. Accessions in bold represent those with the full *Hd1*^{EH}–*Hd2*^{NW}–*Hd4*^{NW} allele combination.

Haplotype	Ratio in 3kRG	Original area and variety name
Hd1^{EH}	39/1436	Argentina(**PRECOZ 2 F A**), Bulgaria(KALIN, PLOVDIV 22, SAEDINENIE), Chile(BULI INIA), China(AI YEH LU, **SHA TIAO TSAO**), Colombia(CT 23, CT 36, CT 58), France(CALENDAL, CIGALON, **MEJANES 2**), Georgia(**WIR 1951**), Greece(MELAS, ROXANI), Hungary(**SZANISZLO 2**), India(T 757), Italy(GIOVANNI MARCHETTI, GITANO, GRITNA, LUXOR, PIEMONTE, REA, SALVO), Philippines(FIDJI, KASIGNAYAN, PARAY QIKUG KABAYUH), Portugal(CAMPINO, ESCARLATE, FAISCA, HAREM, IBO 400, OTA, S 102/2, SAGRES, TOPAZIO), Red River Delta(DELTA), United States(DACCA6)
Hd2^{NW}	23/3015	Argentina(NANOAY P.A, 104 UPLA, **PRECOZ 2 F A**), Belgium(WIR 1072), Bulgaria(AERJITUO), China(HEIMANGDAO, **SHA TIAO TSAO**, LONGHUAMAOHU, MONGOLIA RICE), France(**MEJANES 2**, KELUODUO B), Georgia(**WIR 1951**, WIR 2091), Greece(REA), Hungary(PI 282203, KOPANCSI KEREK, **SZANISZLO 2**, AOMIERTE 168), Italy(GHIBLI, MIARA), Norway(WIR 884), Philippines(FIDJI), United States of America(M 102)
Hd4^{NW}	18/3024	Argentina(**PRECOZ 2 F A**), China(**SHA TIAO TSAO**, HEIMANGDAO), France(**MEJANES 2**, KELUODUO B), Georgia(**WIR 1951**, WIR 2091), Hungary(PI 282203, KOPANCSI KEREK, **SZANISZLO 2**, H 305-84, AUZGUSTA), Italy(SALVO, OPALE, GHIBLI, MIARA, DORELLA), Norway(WIR 884)

Supplemental Data

Supplemental Data 1 | List of 23 weedy rice accessions collected and used in this study. (Separate Excel File)

Supplemental Data 2 | List of 559 published rice accessions representing modern cultivars, landraces, and weedy rice. (Separate Excel File)

Supplemental Data 3 | Conserved genomic regions between European rice and northwestern weedy rice. (Separate Excel File)

Supplemental Data 4 | Summary of *Hd1* haplotypes and their distribution across *japonica* rice groups. (Separate Excel File)

Supplemental Data 5 | Summary of *Hd4* haplotypes and their distribution across *japonica* rice groups. (Separate Excel File)

Supplemental Data 6 | Summary of *Hd2* haplotypes and their distribution across *japonica* rice groups. (Separate Excel File)

Supplemental Data 7 | Representative samples used to predict population history. (Separate Excel File)

Supplemental Data 8 | Genotypes of major heading date genes in *japonica* rice samples used in this study. (Separate Excel File)

Supplemental Data 9 | Statistical analyses in this study. (Separate Excel File)

Supplemental references

Gómez-Ariza, J., Galbiati, F., Goretti, D., Brambilla, V., Shrestha, R., Pappolla, A., Courtois, B., and Fornara, F. (2015). Loss of floral repressor function adapts rice to higher latitudes in Europe. *Journal of Experimental Botany* **66**:2027–2039.

Grimm, A., Fogliatto, S., Nick, P., Ferrero, A., and Vidotto, F. (2013). Microsatellite markers reveal multiple origins for Italian weedy rice Advance Access published 2013.

Ouyang, Z., Jackson, R. B., McNicol, G., Fluet-Chouinard, E., Runkle, B. R. K., Papale, D., Knox, S. H., Cooley, S., Delwiche, K. B., Feron, S., et al. (2023). Paddy rice methane emissions across Monsoon Asia. *Remote Sensing of Environment* **284**:113335.
