# Supplementary material for: Genomic analyses of weedy rice reveal historical connections between European and northwestern Chinese rice
Source: Plant Commun. 2026 Mar 10;7(8):101792. doi: 10.1016/j.xplc.2026.101792 (PMC13477037; doi:10.1016/j.xplc.2026.101792)
Supplement: Supplementary file 2 [file mmc11.pdf]

Plant Communications
Research article

CellPress
Partner Journal

Genomic analyses of weedy rice reveal historical connections between European and northwestern Chinese rice

Yifan Zhong[1,5], Wuyue Chen[1,5], Shuai Xu[1], Qijun Zhang[4], Yixuan Wang[1], Xiaohui Zhang[1], Ju Huang[3,*], Sihai Yang[1,2,*] and Long Wang[1,*]

[1]State Key Laboratory of Pharmaceutical Biotechnology, School of Life Sciences, Nanjing University, Nanjing 210000, China

[2]Co-Innovation Center for Sustainable Forestry in Southern China, Nanjing Forestry University, Nanjing, Jiangsu 210000, China

[3]State Key Laboratory for Crop Genetics and Germplasm Enhancement, Bioinformatics Center, Academy for Advanced Interdisciplinary Studies, Nanjing Agricultural University, Nanjing, China

[4]Institute of Food Crops, Jiangsu Academy of Agricultural Sciences, Nanjing 210014, China

[5]These authors contributed equally to this article.

***Correspondence: Ju Huang (ponyhuang@njau.edu.cn), Sihai Yang (sihaiyang@nju.edu.cn), Long Wang (wanglong@nju.edu.cn)**

https://doi.org/10.1016/j.xplc.2026.101792

ABSTRACT

Rice (*Oryza sativa*) was domesticated in the Yangtze River basin of China and later spread across Asia into Europe; however, the route of its early introduction into Europe remains unresolved, as modern breeding and repeated introductions have obscured historical signals in cultivated varieties. Here, we use weedy rice—a feral derivative of cultivated rice that has undergone relatively limited artificial selection and retains clearer ancestral genomic features—as a genomic system to trace historical dispersal. By integrating whole-genome resequencing of 196 weedy rice accessions with analyses of photoperiod-related flowering genes, we investigated the evolutionary relationships between Asian and European rice populations. Population genomic and phylogenetic analyses reveal a close genetic affinity between European weedy rice and northwestern Chinese rice lineages, while also uncovering signatures of recent feralization and introgression from modern cultivars. Photoperiod-adaptive alleles enriched in northwestern Chinese weedy rice are also prevalent in modern European cultivars, consistent with repeated utilization of ancestral variation during adaptation to higher latitudes. Demographic modeling further suggests that these shared patterns likely predate modern breeding. Together, our findings demonstrate the value of weedy rice as a model for reconstructing crop evolutionary history and provide genetic evidence supporting a historical westward dispersal of rice into Europe, potentially along routes associated with the Silk Road.

Key words: weedy rice, de-domestication, rice dispersal, photoperiod adaptation, Silk Road

Zhong Y., Chen W., Xu S., Zhang Q., Wang Y., Zhang X., Huang J., Yang S., and Wang L. (2026). Genomic analyses of weedy rice reveal historical connections between European and northwestern Chinese rice. Plant Comm. **7**, 101792.

INTRODUCTION

Rice (*Oryza sativa*) is one of the world's most important staple crops, providing sustenance for more than one-third of the global population (Zhang et al., 2024). Its domestication dates back approximately 11 000 years to the middle and lower reaches of the Yangtze River in China (Huang et al., 2012; Zhang et al., 2024). Phylogenetic and archaeological evidence suggests that *japonica* rice was first domesticated in this region and subsequently spread across East and Southeast Asia (Huang et al., 2012; Jing et al., 2023; Zhang et al., 2024). The origin of *indica* rice remains more debated. One hypothesis proposes independent domestication in South Asia (e.g., Londo et al., 2006; Civáň et al., 2015; Jing et al., 2023), whereas another suggests that indica rice emerged through introgression of *japonica*-derived domestication alleles into local wild or proto-domesticated populations in northern India (e.g., Molina et al., 2011; Huang et al., 2012; Choi et al., 2017). Although the overall history of rice domestication is becoming increasingly clear, key questions remain regarding the origins,

 © 2026 The Author(s). Published by Elsevier Inc. on behalf of CAS Center for Excellence in Molecular Plant Sciences, Chinese Academy of Sciences, and Chinese Society for Plant Biology. This is an open access article under the CC BY license (http://creativecommons.org/licenses/by/4.0/).

dispersal routes, and introduction of cultivated rice into Europe, where rice cultivation is dominated by *japonica* varieties.

Several hypotheses have been proposed to explain how *japonica* rice was introduced to the West. One posits that South Asia, rather than East Asia, served as the primary source of domesticated rice for the Middle East and the Mediterranean, based on archaeological and textual evidence (Muthukumaran, 2023). However, this theory is contested because *japonica rice shows* limited adaptation and cultivation scale in South Asia (Shah et al., 2016; Shang et al., 2022).Another hypothesis suggests that *japonica* rice originated in China and entered South Asia via Yunnan, spreading through the foothills of the southern Himalayas into India (Spengler et al., 2021). However, this scenario lacks direct archaeological support. A third hypothesis, based on discoveries from archaeological sites along the ancient Silk Road, proposes that *japonica* rice spread westward from northwestern China (Ren et al., 2024). Excavations in Xinjiang, southern Kazakhstan, southern Uzbekistan, and northern Iran have uncovered *japonica* rice remains dating from approximately 500 BCE to 500 CE (Spengler et al., 2021; Ren et al., 2024), pointing to a possible overland route of diffusion. Nevertheless, the scarcity of archaeological records and the limited, uneven geographic distribution of rice remains outside Xinjiang cast doubt on their relevance for large-scale dispersal. All three hypotheses therefore remain under debate, as they rely primarily on historical and archaeobotanical records and lack strong genetic evidence (Spengler et al., 2021; Muthukumaran, 2023; Ren et al., 2024), leaving the origin and dispersal pathways of *japonica* rice into Europe unresolved.

Europe's rice cultivation history—particularly in Italy, the continent's largest rice producer—is well documented, with records dating back to the 15th century CE (Spada et al., 2004; Courtois et al., 2012). However, archaeological evidence suggests that rice may have been consumed in Europe as early as the 4th century BCE (Spengler et al., 2021). Modern European *japonica* rice has been strongly shaped by breeding programs that incorporated germplasm from both Asia and the Americas (Faivre-Rampant et al., 2011; Courtois et al., 2012). Population genomic studies indicate that modern European *japonica* carries genetic influence from *aromatic* rice types introduced from the Americas (Faivre-Rampant et al., 2011). In contrast, microsatellite fingerprinting of traditional Italian rice varieties points to ancestry from *japonica* landraces originating in northeastern China (Cai et al., 2013). These contrasting findings highlight the difficulty of reconstructing early rice dispersal routes using modern cultivars, as strong population bottlenecks, recent admixture, and extensive human-mediated germplasm exchange can obscure historical genetic signals.

To overcome these limitations, weedy rice (*O. sativa f. spontanea*), a feral derivative of cultivated rice, provides an alternative and highly informative system for investigating rice dispersal and evolutionary history. Because it is less influenced by artificial breeding, weedy rice retains a larger proportion of ancestral genetic diversity, preserving clearer genomic signatures of its introduction and subsequent diversification (Li et al., 2017; Sun et al., 2019). It also exhibits traits such as seed shattering, dormancy, and rapid growth, which enhance its resilience to agricultural management and make it particularly suitable for tracing historical genetic lineages (Sun et al., 2019; Qiu et al., 2020). Moreover, because weedy rice frequently coexists with cultivated rice in similar ecological settings (Qiu et al., 2017, 2020), it offers a valuable comparative framework for examining patterns of genetic continuity and divergence across geographic regions. This characteristic enables the inference of historical dispersal pathways that may be obscured in modern cultivars.

A critical factor influencing the dispersal of rice is its adaptation to different latitudinal environments. As a short-day crop, rice is highly sensitive to photoperiod, which can limit its successful cultivation in high-latitude regions unless specific genetic adaptations are acquired (Eshed and Lippman, 2019). The northward expansion of *japonica* rice into regions such as northern and northwestern China likely required the selection of alleles conferring photoperiod adaptation (Takahashi et al., 2009; Ye et al., 2018; Cui et al., 2020; Lin et al., 2021). Among these, *Hd1* (*Heading Date 1*) has emerged as a key regulator of flowering time, enabling rice to respond to varying day lengths and thereby facilitating reproductive success in new environments (Gómez-Ariza et al., 2015; Nemoto et al., 2016; Wei et al., 2016; Kim et al., 2018). Studies of European rice cultivars have identified distinct *Hd1* variants that support adaptation to the long-day conditions typical of higher latitudes (Goretti et al., 2017). Notably, a transposon-insertion allele ($Hd1^{EH}$) and an allele carrying an amino acid deletion in the CCT(CONSTANS, CO-LIKE and TIMING OF CAB EXPRESSION1) domain ($Hd1^{Vol}$) together account for 64% of *Hd1* alleles in European cultivars (Goretti et al., 2017). These two major haplotypes impair *Hd1* function through distinct mechanisms—transcriptional suppression and disruption of heterotrimeric NF-Y transcription complex binding—thereby reducing photoperiod sensitivity and facilitating adaptation to high-latitude environments (Goretti et al., 2017). Because Europe's rice-growing regions lie at latitudes similar to those of northern and northwestern China (Gutaker and Purugganan, 2024), photoperiod adaptation likely played a key role in the successful establishment of *japonica* rice in Europe. Determining whether these adaptive alleles arose independently, were retained from ancestral populations, or were introduced through gene flow will provide important insights into the origins and dispersal of European rice.

To address these questions, we analyzed whole-genome resequencing data from 582 weedy and cultivated rice accessions collected from Europe, China, and other parts of Asia. Particular emphasis was placed on weedy rice populations from China's Hexi Corridor, a key region along the ancient Silk Road that has been proposed as a major pathway for the westward dispersal of *japonica* rice. Leveraging the unique genetic characteristics of weedy rice—less influenced by modern breeding and more representative of ancestral diversity—we aimed to reconstruct the historical dispersal routes of *japonica* rice into Europe, identify its Asian origins, and clarify the genetic relationships between European weedy rice and cultivated varieties. In addition, we examined whether adaptation to European photoperiod conditions arose through convergent evolution or through the reuse of ancestral adaptive alleles. We also assessed the extent of gene flow between modern European cultivars and weedy rice populations, with a particular focus on genes associated with photoperiod adaptation.

By integrating genomic and phylogenetic analyses with historical and archaeological evidence, this study provides new insights into the origins and dispersal history of European *japonica* rice. Our results highlight the potential role of ancient landraces and weedy rice in preserving ancestral genetic variation, particularly for adaptive traits such as photoperiod sensitivity. More broadly, this work demonstrates the value of weedy rice as a complementary genetic archive for tracing crop migration pathways, offering an evolutionary perspective on domestication, geographic dispersal, and environmental adaptation while acknowledging the inherent uncertainties in reconstructing historical processes.

RESULTS

Weedy rice samples and genomic sequencing data collection

Given that cultivated rice varieties in Europe are predominantly of the *japonica* type (Courtois et al., 2012), this study focused primarily on *japonica*-type rice, including both cultivated varieties and weedy rice collected from diverse geographic regions. Previous genomic studies have elucidated the evolutionary trajectories of weedy rice in Asia and the United States (Qiu et al., 2017, 2020; Sun et al., 2019; Li et al., 2024a). However, a notable gap remains in the representation of weedy rice populations from northwestern China—a region that has played a pivotal role in the global dispersal of rice because of its geographic and historical significance. To address this gap, we collected and sequenced 23 new weedy rice samples (16 *japonica* type and 7 *indica* type) from 13 provinces in China, including eight samples from northwestern China (Supplemental Data 1).

The newly collected samples exhibited characteristic traits of weedy rice, including reddish-brown seed coats, awned spikelets, and high seed-shattering rates (Supplemental Figure 1). Whole-genome sequencing of these samples achieved an average depth of 31.2× and covered 99.93% of the genome when aligned to the *japonica* Nipponbare reference genome (Supplemental Data 1). This dataset substantially enhances our capacity to reconstruct the evolutionary relationships among modern *japonica* cultivars, landraces, and weedy rice populations across Eurasia.

In total, we compiled a dataset of 196 high-quality weedy rice genomes (164 *japonica* type and 32 *indica* type; average sequencing depth of 19.8× and a mapping rate of 99.04%) by integrating our 23 newly sequenced samples with 173 publicly available weedy rice genomes from diverse geographic regions (Supplemental Data 1 and 2). Because the high-latitude climate of Europe favors *japonica*-type rice due to its shorter growing season and photoperiodic adaptation (Courtois et al., 2012), we further incorporated 355 published genomes of *japonica* cultivated rice (Supplemental Data 2). These included major cultivars from key *japonica*-growing regions in Asia and Europe, as well as several landraces from East Asia. To capture genetic contributions associated with modern breeding, we additionally included 16 *aus* and 15 *aromatic* rice accessions. The 32 *indica*-type weedy rice samples, which do not share a direct lineage with European rice, were used as outgroups for phylogenetic analyses.

Among the 582 collected samples, our analyses focused primarily on 355 cultivated *japonica* rice and 196 weedy rice genomes, representing the major *japonica*-growing regions across Eurasia (Figure 1A). Following stringent quality control (see methods), we identified 6 715 815 high-quality single-nucleotide polymorphisms (SNPs), of which 19.3% were located within coding regions. Among these, 727 337 SNPs are predicted to result in nonsynonymous substitutions, whereas 538 326 correspond to synonymous variants relative to the reference genome. This high-resolution dataset provides a solid foundation for investigating the phylogenetic relationships, population structure, and demographic history of *japonica* cultivars and weedy rice across the sampled regions.

Most European weedy rice shares closer ancestry with northwestern Chinese landraces and weedy rice

Using 6.72 million SNPs, we constructed a maximum-likelihood phylogenetic tree with high bootstrap support (Figure 1C and Supplemental Figure 2; >94.5% of branches with bootstrap values $\geq$ 95%), which clearly separates the *japonica* clade from the *indica*, *aromatic*, and *aus* outgroups, consistent with previous studies (Huang et al., 2012). Within the *japonica* clade, modern cultivars are separated from weedy rice, forming three distinct clades designated clades 1, 2, and 3 (Figure 1C). Clades 1 and 3 represent modern cultivar groups: clade 1 contains 91.2% of European modern cultivars, whereas clade 3 contains 97.0% of Asian modern cultivars. The tree topology places clade 1 between the outgroups and clades 2 and 3, indicating a more peripheral relationship to the latter two groups.

Clade 2 comprises the majority of *japonica* weedy rice samples from both Asia and Europe (140/164), as well as most Chinese landraces (32/37). This pattern is consistent with previous findings showing that European weedy rice is genetically closer to Asian weedy rice than to modern European cultivated rice (Sun et al., 2019). The clustering of most *japonica*-type landraces and weedy rice samples within clade 2 suggests that weedy rice populations in both Europe and Asia likely originated from ancient Chinese landraces and dispersed alongside them during early rice migration events.

Within clade 2, we identified at least five distinct subgroups—designated weed-NW, weed-EU, land-E, weed-NE, and weed-NEA—each closely associated with specific geographic regions (Figure 1C). The weed-NW subgroup comprises six landraces and fourteen weedy rice samples from northwestern China. Weed-EU is dominated by European weedy rice (23 of 29 samples) and also includes two modern European cultivars. Land-E primarily consists of landraces (19 of 24) from the lower Yangtze River region in eastern China, which is widely recognized as the center of *japonica* rice domestication (Huang et al., 2012; Zhang et al., 2024). Weed-NE includes 59 weedy rice samples and six landraces, mostly from northeastern China, as well as one modern cultivar from Europe and one from Japan. Finally, weed-NEA comprises 42 weedy rice samples collected exclusively from northeastern Asia outside China.

The close phylogenetic relationship between weed-NW and weed-EU suggests that European weedy rice—and some modern cultivars—share a common ancestry with landraces and weedy rice from northwestern China (Figure 1C). Clade 2 is primarily composed of Asian landraces and Eurasian weedy rice, within which northwestern Chinese samples cluster closely

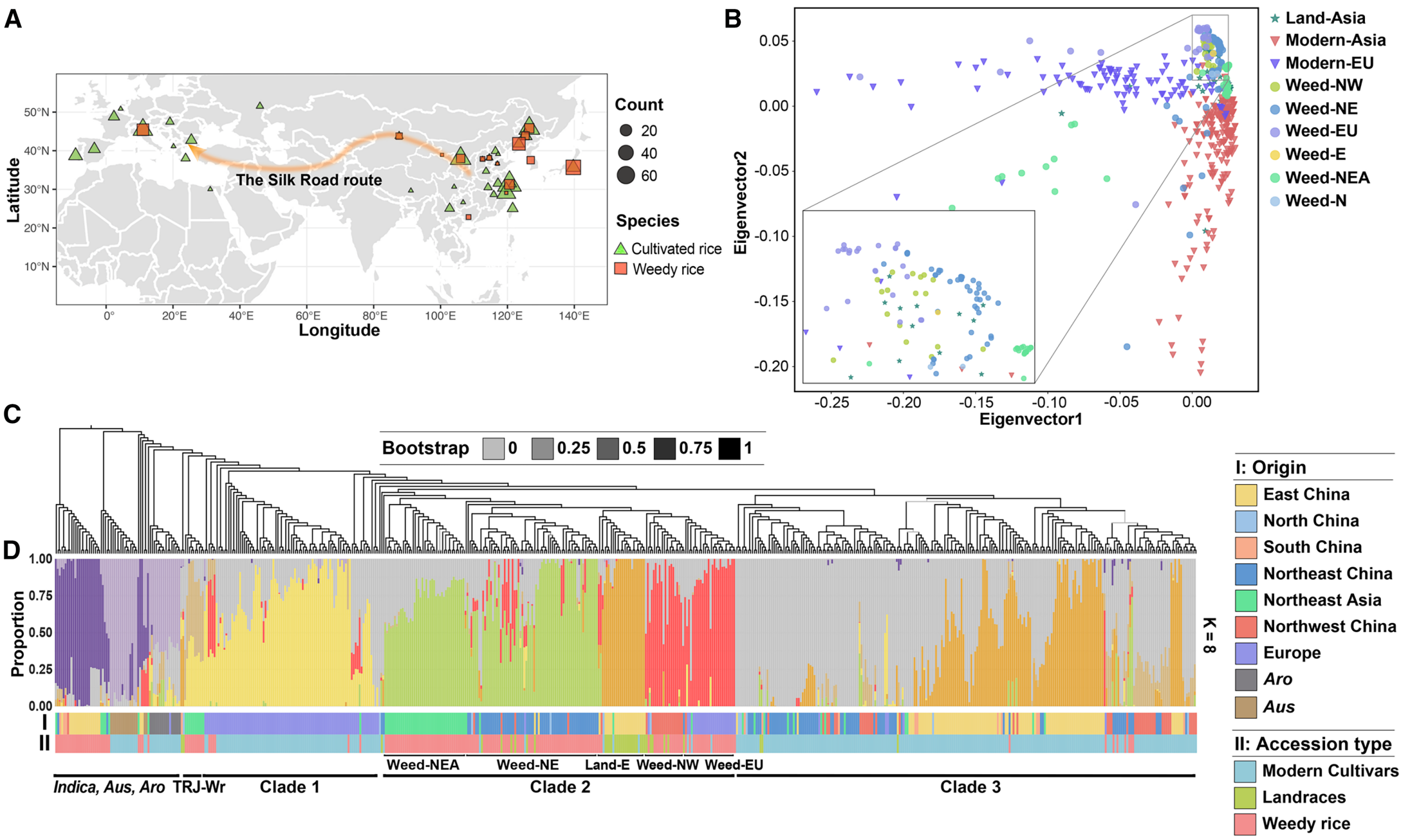

Figure 1. Geographic origin and population structure of the rice samples analyzed.

(A) Geographic distribution of rice samples across Eurasia: cultivated rice (green triangles) and weedy rice (red squares).

(B) Principal component analysis (PCA) of *japonica* samples. Landraces are indicated by stars, cultivated rice by triangles, and weedy rice by circles. The positions of European weedy rice and northwestern Chinese weedy rice are highlighted in enlarged rectangular boxes.

(C) Phylogenetic tree of *japonica* cultivated and weedy rice from East Asia and Europe, with *indica* weedy rice used as outgroups. Annotation bars beneath the phylogenetic tree indicate geographic origin **(I)** and sample classification **(II)**. Accession types are color- coded as follows: modern cultivars (blue), landraces (green), and weedy rice (red). Branch shading represents bootstrap support (1,000 replicates).

(D) Population structure of *japonica* cultivated and weedy rice in East Asia and Europe (maximum-likelihood clustering at K = 8). Labels: Modern-, modern cultivars; Land-, landraces; Weed-, weedy rice. Geographic abbreviations: EU, Europe; E, east China; N, north China; NE, northeast China; NW, northwest China; S, south China; NEA, northeast Asia.

with European weedy rice. This pattern is consistent with a scenario in which European weedy rice derives from ancestral populations related to northwestern Chinese rice.

To validate the phylogenetic relationships within *japonica*-type rice, we performed principal component analysis (PCA) and population structure analysis. The PCA (Figure 1B) clearly separates Asian modern cultivars from Asian landraces, European cultivars, and weedy rice. Notably, European weedy rice clusters closely with northwestern Chinese weedy rice, indicating a strong genetic affinity between these groups. Population structure analysis (Figure 1D) further supports this pattern, showing that European weedy rice and some European cultivars are more closely related to northwestern Chinese weedy rice than to modern European *japonica* cultivars.

Variation in photoperiod genes supports a northwestern Chinese origin of European weedy rice

Rice is a short-day plant with strong photoperiod sensitivity (Nemoto et al., 2016), a trait that historically limited its northward expansion from the Yangtze River domestication center (Huang et al., 2012; Jing et al., 2023). As a result, the co-ancestry of key photoperiod-related genes can provide valuable insights into the dispersal history of rice accessions. The similar latitudes of northwestern China and major European rice-growing regions suggest that, if European weedy rice (specifically those from clade 2) indeed originated from northwestern China, consistent genotypes at key photoperiod-related genes should be observed among European weedy rice, northwestern Chinese weedy rice, and landraces from northwestern China, regardless of when they were introduced.

To test this hypothesis, we analyzed six key photoperiod-related genes known to play important roles in high-latitude adaptation: *Hd1*, *Ehd1*, *Hd2*, *Hd4*, *Hd5,* and *RFT1* (Xue et al., 2008; Takahashi et al., 2009; Fujino et al., 2013; Koo et al., 2013; Wei et al., 2016). We found that *Ehd1* and *RFT1*, which function as positive regulators of flowering under long-day conditions, are highly conserved in *japonica* weedy rice populations, limiting their usefulness for origin inference (Supplemental Data 8). Similarly, *Hd5*, a repressor of flowering under long-day conditions, is also highly conserved across *japonica* weedy rice populations, suggesting little or no contribution to the high-latitude adaptation of European weedy rice (Supplemental Data 8). In contrast, the remaining three genes—also negative regulators of long-day flowering—

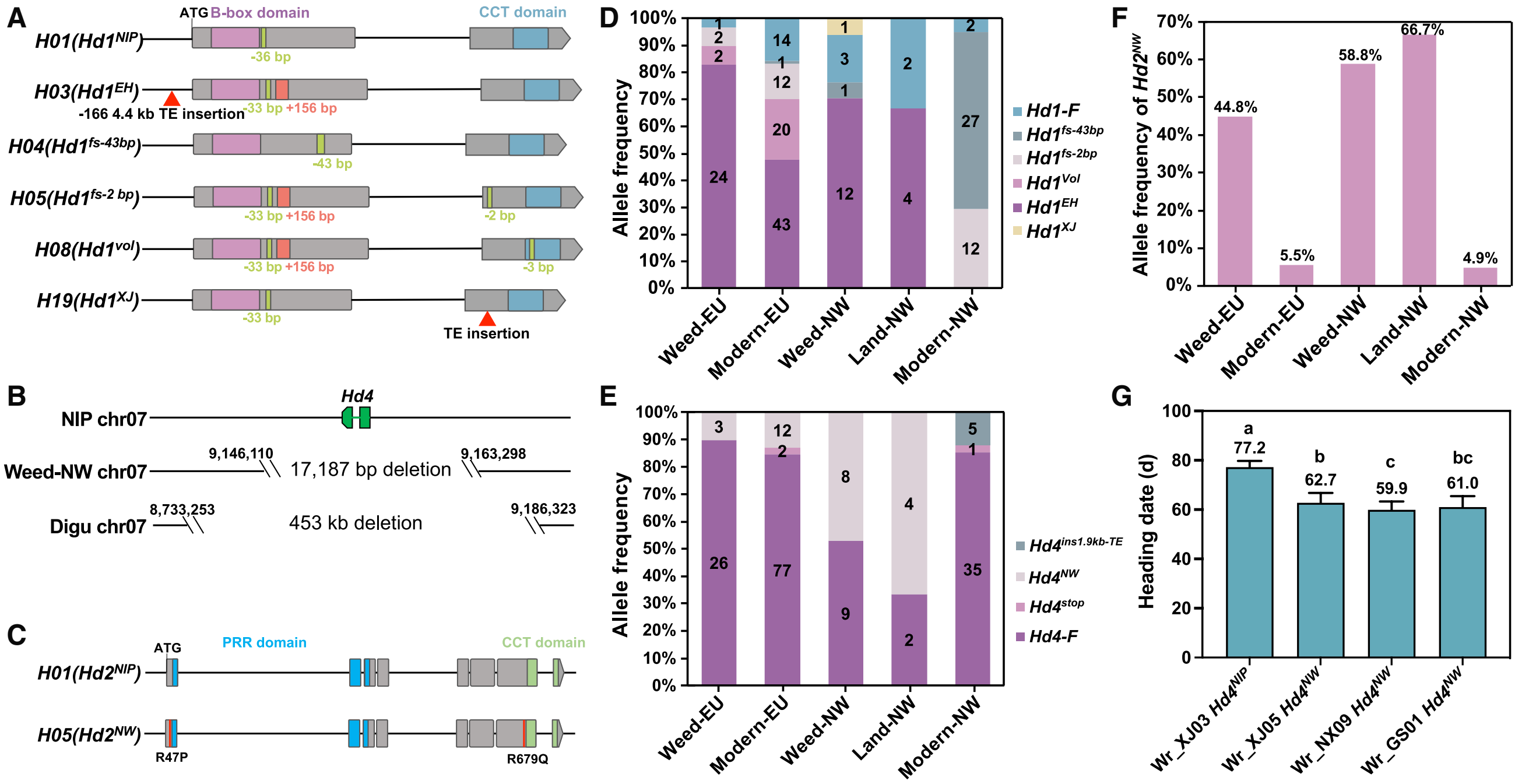

Figure 2. Genetic variation and functional analysis of photoperiod genes in European and northwestern Chinese rice.

(A) Comparison of the *Hd1* promoter and coding sequences among four nonfunctional genotypes and one novel genotype identified in northwestern Chinese weedy rice and European cultivars vs. Nipponbare. The coding region is shown as a gray rectangle. The B-box zinc-finger domain and CCT domain are indicated by lilac and cyan rectangles, respectively. Green and red rectangles denote insertions and deletions, respectively, and red triangles indicate transposon insertions.

(B) Comparison of the *Hd4* gene and its flanking regions among Nipponbare, northwestern Chinese weedy rice, and the *indica* variety Digu. Nipponbare carries the intact *Hd4* genomic region, whereas Digu harbors a 453-kb chromosomal deletion. In contrast, northwestern Chinese weedy rice exhibits a distinct 17 187-bp deletion in this region.

(C) Gene structure comparison between $Hd2^{NW}$ and the Nipponbare-type *Hd2*. The PRR (PSEUDO-RESPONSE REGULATOR) domain and CCT domain are indicated by blue and green rectangles, respectively. Missense variants relative to the Nipponbare *Hd2* haplotype are indicated by red vertical lines.

(D) Proportions of functional and nonfunctional *Hd1* alleles across northwestern Chinese and European rice populations. Sample sizes are indicated within the bars.

(E) Proportions of *Hd4* alleles across the same populations. Sample sizes are indicated within each bar.

(F) Allele frequency of $Hd2^{NW}$ in northwestern Chinese and European rice populations.

(G) Weedy rice carrying $Hd4^{NW}$ flowers earlier than those carrying $Hd4^{NIP}$ in a genetic background with nonfunctional *Hd1* and functional *Hd4* and *Hd5*. Different letters indicate statistically significant differences (Student's *t*-test, $P < 0.001$; see Supplemental Data 9 for statistical analyses). Genotype superscripts denote the following: fs-4bp, 4-bp frameshift deletion; fs-43bp, 43-bp frameshift deletion; ins1.9kb-TE, insertion of a 1.9-kb transposable element; stop, premature stop codon. Population abbreviations: weed-EU, European weedy rice; modern-EU, European modern cultivars; weed-NW, northwestern Chinese weedy rice; land-NW, northwestern Chinese landraces; modern-NW, northwestern Chinese modern cultivars.

particularly *Hd1*, show clear geographic differentiation and functional variation across different geographic regions (Supplemental Data 4), making them informative markers for testing the hypothesis above.

Hd1 is widely recognized as a key determinant of photoperiod adaptation in European rice cultivated at high latitudes (Goretti et al., 2017). Our analysis identified 20 distinct *Hd1* haplotypes across 513 *japonica* samples (Supplemental Data 4). We also discovered a previously unreported *Hd1* haplotype in Xinjiang weedy rice, which we named $Hd1^{XJ}$ (Figure 2A; see also H19 in Supplemental Data 4). This haplotype contains a transposon insertion in the second exon, which may affect its functional integrity. A high proportion (82.3%) of northwestern Chinese weedy rice accessions carried nonfunctional *Hd1* alleles (including H03, H04, and the novel $Hd1^{XJ}$), suggesting an important role for *Hd1* variation in facilitating adaptation to high-latitude environments. The haplotype H03 identified in our study corresponds to the previously reported $Hd1^{EH}$ (Figure 2A and Supplemental Figures 3A and 3B) (Goretti et al., 2017). This haplotype was highly prevalent in northwestern Chinese landraces (66.7%) and weedy rice (70.6%), but rare elsewhere globally (3.5%) and completely absent from modern northwestern Chinese cultivars (Figure 2D; Supplemental Data 4). Consistent with the proposed Chinese origin of European weedy rice, 82.8% of European weedy rice samples (24/29) also carried $Hd1^{EH}$ (Figure 2D).

Functional comparisons showed that weedy rice lines carrying $Hd1^{EH}$ flowered earlier than those harboring functional *Hd1* haplotypes prevalent in European cultivated or weedy rice (Supplemental Figure 4A). In 2 years of field trials in Nanjing, $Hd1^{EH}$ plants flowered on average 10.6 days earlier (Supplemental Figure 4A), and this effect remained stable across different latitude conditions (Supplemental Figure 4C; Supplemental Note 1). Moreover, flowering time did not differ significantly between $Hd1^{EH}$ and another loss-of-function allele,

Hd1$^{fs\text{-}2bp}$, confirming that *Hd1*^{EH} is nonfunctional (Supplemental Figure 4A). Phylogenetic analysis of *Hd1* further showed that most European weedy rice, together with northwestern Chinese landraces and weedy rice, clustered within the same branch (including weed-EU and weed-NW of clade 2), all carrying the *Hd1*^{EH} allele (Supplemental Figure 5).

Similar to *Hd1*, the allelic distribution of *Hd4* further supports a northwestern Chinese origin for European weedy rice (Supplemental Figure 7). Notably, we identified a previously unreported *Hd4* genotype, designated *Hd4*^{NW}, characterized by a 17 187-bp deletion that eliminates the entire *Hd4* gene (Figure 2B; Supplemental Figures 3A and 3C). Although this resembles the ∼453-kb deletion reported in the southwestern *indica* variety Digu (Figure 2B), the distinct size and breakpoints indicate an independent origin. Field experiments demonstrated that weedy rice carrying *Hd4*^{NW} flowered on average 16.0 days earlier than lines with the functional allele, consistent with the expected loss-of-function phenotype of *Hd4* (Xue et al., 2008; Zong et al., 2021; Sun et al., 2022) (Figure 2G and Supplemental Figure 4B; Supplemental Note 1).

Hd4^{NW} was detected exclusively in northwestern Chinese landraces (66.7%, 4/6 samples), weedy rice (47.1%, 8/17 samples), and European weedy rice (10.3%, 3/29 samples) and was absent from all other global rice populations (Figure 2E; Supplemental Data 5). Its relatively low frequency in both weedy and modern European rice (Figure 2E) likely reflects stronger selective constraints on *Hd4*, as functional alleles are known to enhance yield and nitrogen-use efficiency in cultivars (Wang et al., 2021; Lou et al., 2025) and improve environmental resilience in weedy rice under high-latitude conditions (Herath, 2019; Nagalla et al., 2021; Li et al., 2022; Vicentini et al., 2023).

Additionally, we identified a novel haplotype of *Hd2* that carried a G-to-A substitution that results in an amino acid change from a positively charged arginine (Arg) to a neutral glutamine (Gln) at position 679 of the Hd2 protein (Figure 2C). This haplotype is widely distributed in northwestern Chinese landraces (4/6 samples), weedy rice (10/17 samples), and European weedy rice (13/29 samples) but occurs only sporadically in other populations (7 samples from other regions; Figure 2F; Supplemental Data 6). Because this mutation lies near the C-terminal CCT domain of *Hd2* and alters the amino acid charge, it may have functional significance and warrants further investigation.

The observed genetic structuring of *Hd1*, *Hd4*, and *Hd2* indicates that European weedy rice shares early-flowering alleles—particularly at *Hd1* and *Hd4*—with northwestern Chinese weedy rice, reflecting parallel adaptation to high-latitude photoperiod conditions. These patterns are consistent with the genome-wide phylogenetic inference that clade 2 European weedy rice likely originated from northwestern China.

Shared photoperiod alleles between European weedy and cultivated rice indicate an early link to northwestern China

The analyses above support the conclusion that clade 2 European weedy rice most likely originated from northwestern China. However, it remains unclear whether this shared ancestry reflects a relatively recent introduction or an ancient co-dispersal with early European cultivated rice, followed by subsequent divergence from modern cultivars.

Because modern European cultivated rice largely derives from *aus* varieties introduced from North America in recent centuries (Faivre-Rampant et al., 2011; Grimm et al., 2013), two contrasting scenarios may explain the evolution of photoperiod adaptation genes. One possibility is that modern European cultivars underwent independent selection, resulting in novel genotypes specifically adapted to high-latitude photoperiod conditions. Under this scenario, substantial genetic divergence in photoperiod-related genes would be expected between modern European cultivars and older European rice lines, European weedy rice, and northwestern Chinese weedy rice and landraces. Alternatively, modern European cultivars may have reused photoperiod adaptation alleles from earlier European rice varieties that were already adapted to local photoperiod conditions. In this case, if European weedy rice was introduced alongside these earlier cultivars—or arose through escape from them—before the advent of modern rice breeding in Europe, modern European cultivars would be expected to retain some ancestral photoperiod adaptation alleles. Such a pattern would indicate a shared historical connection among European weedy rice, earlier cultivated rice varieties with similar photoperiod genotypes, and northwestern Chinese rice lineages. This relationship may reflect ancient westward dispersal along routes such as the Silk Road, a historically important pathway for the spread of domesticated crops (Zhou et al., 2020; Kang et al., 2021; Huang et al., 2023; Gutaker and Purugganan, 2024).

To test these two hypotheses, we identified 16.3 Mb of conserved genomic regions (Figure 3A; Supplemental Data 3) that may have contributed to European cultivars through ancestral introductions, by searching for genomic segments shared among northwestern Chinese weedy rice, European weedy rice, and European cultivated rice (see methods for details). These conserved regions contain eight well-characterized heading-date genes known to influence photoperiod sensitivity, including the previously examined *Hd1*, as well as *Hd6*, *Hd16*, *RCN4*, *SDG725*, *OsCCT29*, *OsCCT39,* and *OsCCT40* (Figure 3A and Supplemental Figure 6). We further examined allelic variation and phylogenetic relationships of these genes across different rice populations.

Haplotype analysis of *Hd1* identified six genotypes among 91 European cultivars, whereas the 29 European weedy rice samples contained four genotypes, all of which overlapped with those detected in the cultivars (Figures 2A and 2D; Supplemental Data 4). In our dataset, *Hd1*^{EH} accounted for 47.8% of European cultivars, consistent with the previously reported 45% frequency (Goretti et al., 2017), although this proportion is lower than the 82.8% observed in European weedy rice (Figure 2D).

Within the *Hd1* phylogeny, a substantial proportion—nearly half—of European cultivars carrying *Hd1*^{EH} were located within the modern European cultivar branch (clade 1) and clustered with European weedy rice and northwestern Chinese weedy rice (Supplemental Figure 5). We further investigated the

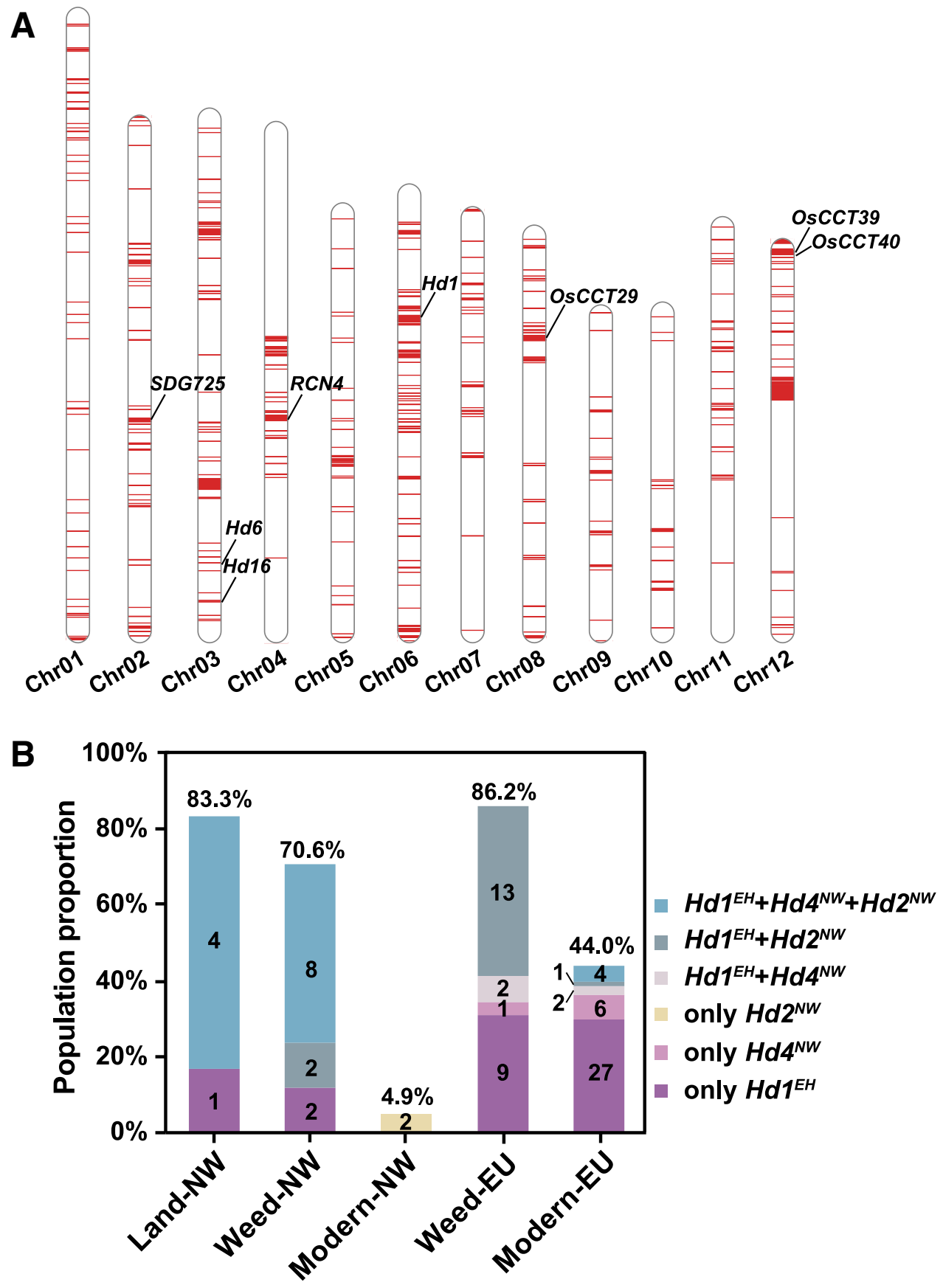

Figure 3. Conserved genomic regions and the distribution of photoperiod-related alleles in northwestern Chinese and European rice populations.

(A) Chromosomal distribution of conserved genomic regions shared among northwestern Chinese weedy rice, European weedy rice, and European cultivated rice. Conserved regions are highlighted with red bars, and genes known to be involved in photoperiod regulation are indicated. **(B)** Distribution of *Hd1*^{EH}, *Hd4*^{NW}, and *Hd2*^{NW} alleles and their combinations in northwestern Chinese and European rice populations. Percentages on the bars indicate the proportion of individuals carrying at least one NW-oriented flowering-time allele. Numbers above the bars denote the sample size for each allele type. Land-NW, northwestern Chinese landraces; weed-NW, northwestern Chinese weedy rice; modern-NW, northwestern Chinese modern cultivars; weed-EU, European weedy rice; modern-EU, European modern cultivars.

population-level phylogenetic relationships of seven additional heading-date genes and observed a consistent pattern in which northwestern Chinese weedy rice, European weedy rice, and European cultivated rice shared the same evolutionary branches (Supplemental Figure 8).

Given the widespread occurrence of *Hd1*^{EH} and other shared alleles in both northwestern Chinese and European rice populations, particularly in modern European cultivars, these patterns suggest that this haplotype was likely introduced into Europe at an earlier stage. This finding is consistent with the hypothesis that European weedy rice may have arrived alongside early cultivated rice varieties carrying the same genotype, potentially through historical dispersal routes such as the ancient Silk Road.

Genetic linkage of *Hd2*^{NW} and *Hd4*^{NW} with *Hd1*^{EH} further supports an early northwestern Chinese origin of European rice

Rice adaptation to high-latitude photoperiod conditions is shaped not only by functional variation in individual genes but also by the combination and interaction of alleles at multiple flowering-time loci (Ye et al., 2018; Cui et al., 2020). To explore this, we analyzed the combination patterns of key photoperiod-related genes shared between European and northwestern Chinese rice populations. In northwestern China, all four landraces carrying the *Hd1*^{EH} genotype also harbored *Hd4*$^{NW\ and}$ *Hd2*^{NW}, while among the 12 *Hd1*^{EH}-carrying weedy rice samples, eight also contained *Hd4*$^{NW\ and}$ *Hd2*^{NW} (Figure 3B). In other words, although these loci reside on different chromosomes (*Hd1*^{EH} on chromosome 6 and *Hd4*^{NW} and *Hd2*$^{NW\ on}$ chromosome 7), every northwestern Chinese sample (landrace or weedy rice) with detectable *Hd4*^{NW} and *Hd2*^{NW} also carried *Hd1*^{EH}. This pattern indicates strong genetic linkage among these three loci and suggests an ancient allele combination that may enhance adaptation to high-latitude environments.

In European populations, several cultivars and weedy rice samples also retained *Hd1*^{EH}, *Hd4*^{NW,} and *Hd2*^{NW} (Figure 3B), underscoring the functional significance of this allele combination in photoperiod adaptation. This pattern is further supported by their geographic distribution in the 3K Rice Genomes Database (Supplemental Table 4; Supplemental Note 2). The four European cultivars carrying all three alleles clustered within the modern European cultivar branch (clade 1) in the genome-wide phylogeny (Supplemental Figure 2), consistent with a northwestern Chinese origin. In contrast, most European weedy rice samples retained only one or two of these alleles, likely reflecting allele loss or reshuffling during de-domestication. A similar trend was observed in northeastern China, where weedy rice populations carried fewer defective flowering-time alleles than local modern cultivars (Supplemental Data 8).

The distribution of these three photoperiod-related alleles further supports an early connection between European and northwestern Chinese rice populations that predates modern breeding. It also suggests parallel evolutionary trajectories between weedy and cultivated rice following the early introduction of rice into Europe. The retention of these genotypes in European rice populations indicates that modern breeding has repeatedly exploited pre-existing photoperiod-adaptive variation, consistent with a historical introduction from northwestern China.

Evidence of introgression from modern cultivars into European weedy rice

Although most European weedy rice samples cluster with northwestern Chinese weedy rice, a few fall at the periphery of—or within—the European cultivated group (clade 1). To determine whether these weedy rice samples represent recent escapes from modern cultivars or older weedy lineages affected by gene flow, we grouped the weedy rice and cultivars in clade 1 according to whether they carried NW-oriented flowering-time alleles. We then quantified identity-by-descent (IBD) segment sharing with weed-NW and weed-EU in clade 2 for each group. We found that weedy rice carrying NW-oriented flowering-time alleles

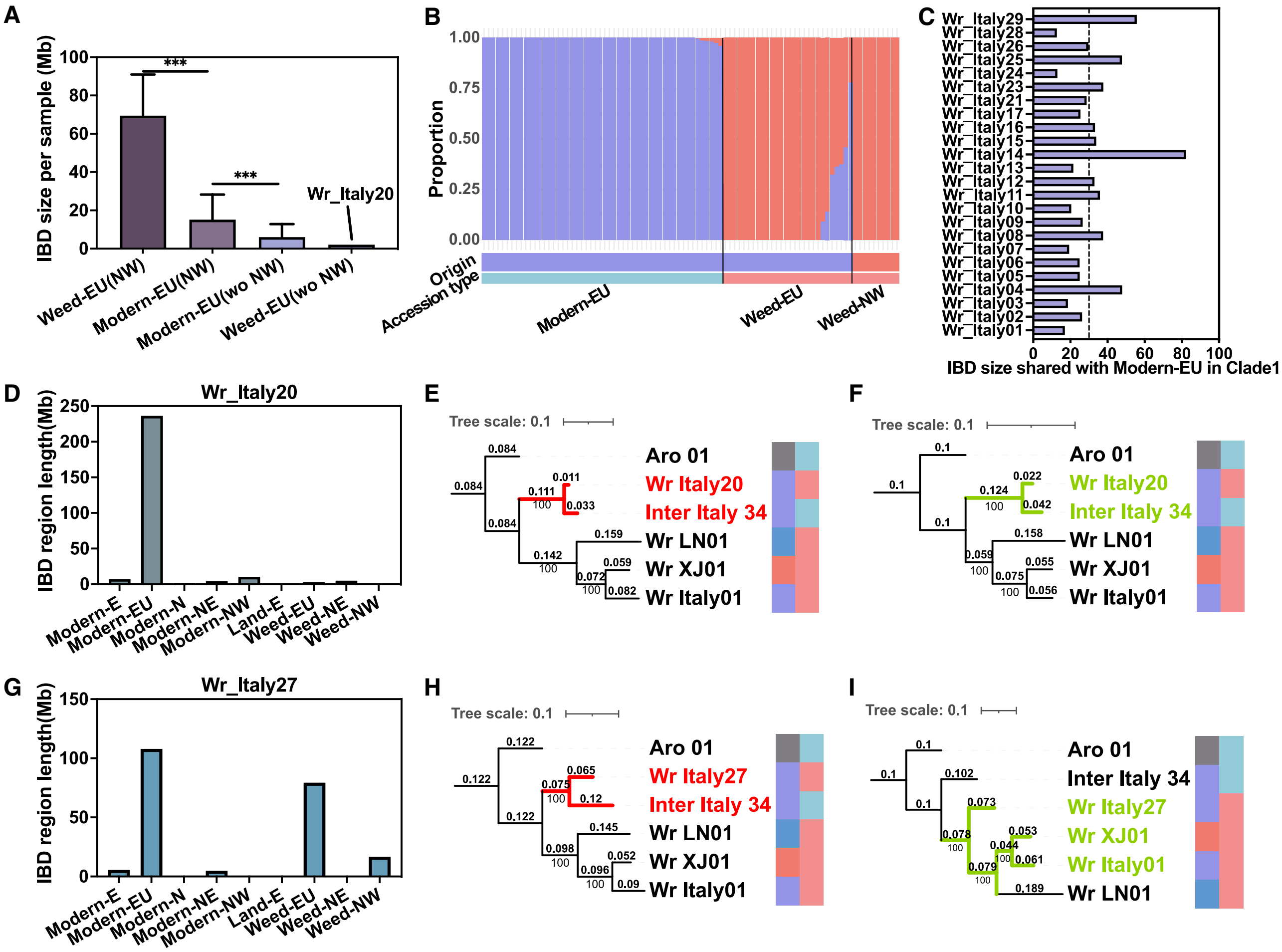

Figure 4. Gene introgression from European cultivated rice to European weedy rice.
(A) Comparison of IBD segment lengths shared between the four clade 1 groups and weed-EU and weed-NW in clade 2 (see Supplemental Data 9 for statistical analyses). NW, individuals carrying NW-oriented alleles; wo NW, individuals without NW-oriented alleles.
(B) Admixture analysis (K = 2) showing genetic contributions of European cultivated rice to European weedy rice.
(C) Size distribution of IBD segments shared between weed-EU in clade 2 and modern-EU in clade 1.
(D and G) Size distributions of IBD segments shared between individual European weedy rice accessions (**D,** Wr_Italy20; **G,** Wr_Italy27) and different rice populations.
(E and H) Phylogenetic trees constructed from IBD segments shared between Wr_Italy20 and modern-EU **(E)** and between Wr_Italy27 and modern-EU **(H)**. Red branches indicate the closest relatives of the European weedy rice accessions.
(F and I) Phylogenetic trees constructed based on non-IBD segments for comparison. Green branches indicate the closest relatives of the European weedy rice accessions. Population abbreviations: modern-E, eastern Chinese modern cultivars; modern-EU, European modern cultivars; modern-N, northern Chinese modern cultivars; modern-NE, northeastern Chinese modern cultivars; modern-NW, northwestern Chinese modern cultivars; land-E, eastern Chinese landraces; weed-EU, European weedy rice; weed-NE, northeastern Chinese weedy rice; weed-NW, northwestern Chinese weedy rice.

exhibited significantly higher levels of IBD sharing with weed-NW and weed-EU than both cultivated rice groups and weedy rice lacking these alleles (Figure 4A; statistical analyses are provided in Supplemental Data 9). These results indicate that these weedy rice individuals likely descended from pre-existing weedy rice populations that later experienced introgression from modern cultivars, rather than originating from *de novo* escapes of clade 1 cultivars carrying the same flowering-time alleles.

Notably, one sample (Wr_Italy20) is phylogenetically closest to modern European cultivars and clearly distinct from northwestern Chinese samples (Figure 1C and Supplemental Figure 2), sharing minimal IBD segments with weed-NW and weed-EU (Figures 4A and 4D). Wr_Italy20 clusters tightly with European modern cultivars, suggesting a likely origin from local cultivated varieties. Unlike the NW-originated weedy rice, Wr_Italy20 carries the *Hd1*$^{fs\text{-}2bp}$ allele as well as functional alleles of *Hd2* and *Hd4* (Supplemental Figure 2), consistent with the genotype of European cultivars. Its markedly different genomic profile and photoperiod genotypes further support a local origin. Wr_Italy20 also shares extensive IBD segments (~236 Mb) with Modern-EU (Figure 4D), and this close relationship persists even after excluding IBD regions (Figures 4E and 4F), indicating a recent escape from the modern-EU lineage. Based on phylogenetic relationships and IBD sharing (Figure 1C and Supplemental Figure 2; Supplemental Table 1), we identify the cultivated variety "Gladio" (Inter_Italy_34) as the most likely ancestral source of Wr_Italy20.

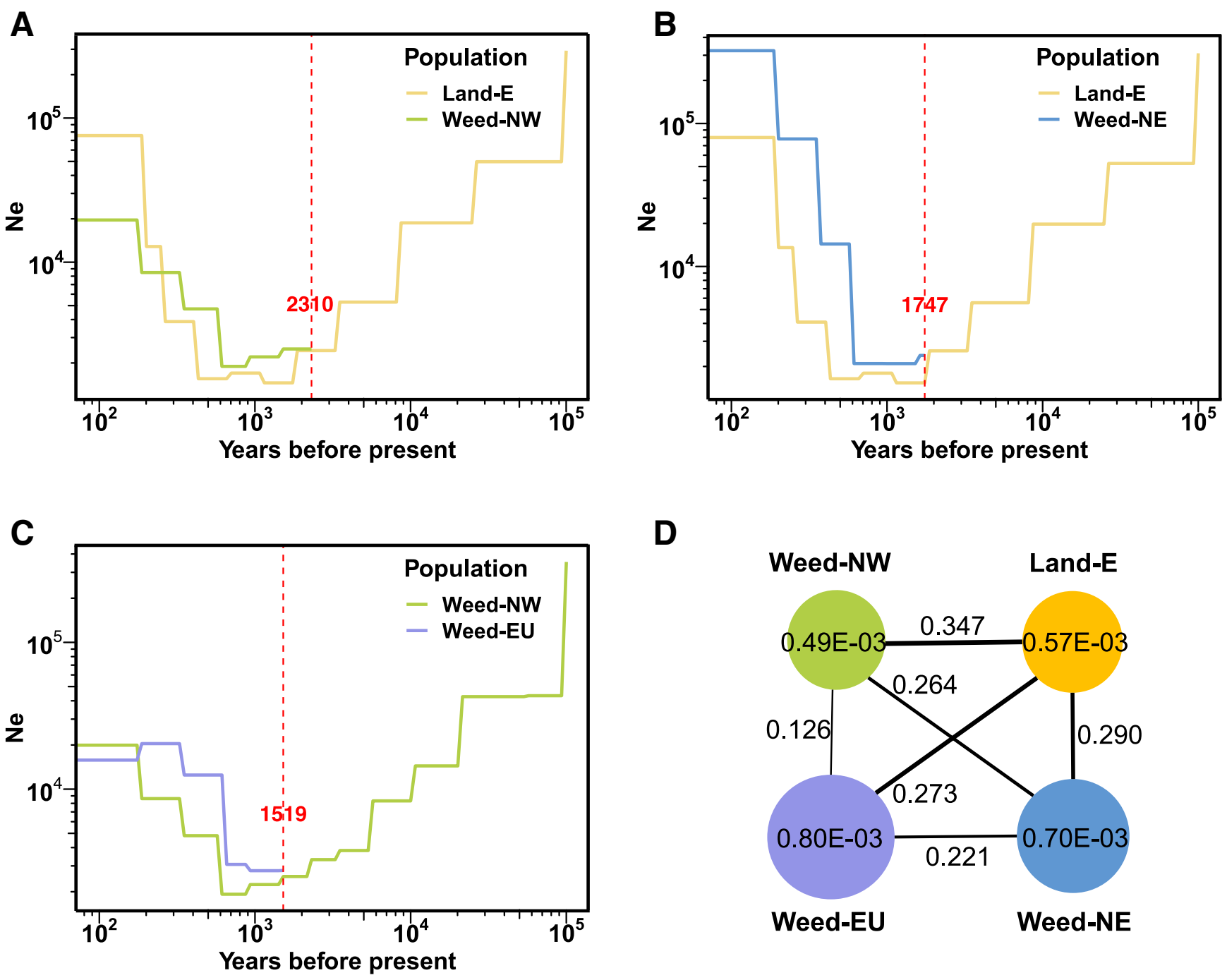

Figure 5. Demographic history, genetic differentiation, and nucleotide diversity of weedy rice populations.

(A–C) Changes in effective population size (N_e) over time for European weedy rice (weed-EU), northeastern Chinese weedy rice (weed-NE), and northwestern Chinese weedy rice (weed-NW) populations, with divergence times estimated using SMC++ (red vertical lines).

(D) Summary of nucleotide diversity (π) and genetic differentiation (F_{ST}) among populations. Circle size and internal values represent nucleotide diversity, whereas line thickness and external values indicate F_{ST} between populations. Population abbreviations: land-E, eastern Chinese landraces; weed-NW, northwestern Chinese weedy rice; weed-NE, northeastern Chinese weedy rice; weed-EU, European weedy rice.

The remaining six European weedy rice samples (including four in clade 1) exhibit admixed origins from both northwestern Chinese rice and local European cultivars (Figure 4B). At least 10 samples contain substantial IBD segments derived from modern-EU ($\geq$30 Mb) yet maintain shorter genomic distances to weed-EU and weed-NW samples (Figures 1C and 4C; Supplemental Table 2), supporting recent introgression events. Notably, their photoperiod-related genes retain the same alleles and combinations observed in weed-EU (Supplemental Figure 2). These introgressed segments, together with *de novo* escape events, may enhance adaptability in feral environments and warrant further investigation.

Exceptionally, the sample Wr_Italy27 exhibits a longer IBD segment with modern-EU (107.90 Mb) than with weed-EU (79.40 Mb) (Figure 4G), while its non-IBD regions show closer genetic distances to weed-NW and weed-EU (Figures 4H and 4I). This pattern likely reflects recent introgression from modern cultivars into weedy rice. Wr_Italy27 also carries a unique combination of photoperiod genotypes—$Hd1^{fs\text{-}2bp}$ and $Hd4^{NW}$ (Supplemental Figure 2), indicating a recombinant genetic background derived from both modern cultivars and ancient weedy lineages.

Overall, our findings suggest that the subsequent evolution of European weedy rice, following its possible introduction from northwestern China, has been continually shaped by local modern cultivars, either through gene introgression or *de novo* escape events. This pattern reflects the breeding history and genetic legacy of regional cultivated varieties.

Population history supports the introduction of European weedy rice via the Hexi Corridor

Unlike cultivated rice, which has experienced extensive gene flow due to breeding practices that complicate demographic inference, weedy rice has been relatively less affected by artificial selection. This makes it a valuable proxy for tracing the historical dispersal of rice populations. To investigate the demographic history of northwestern-derived weedy rice, we used SMC++ to analyze representative samples from major lineages within clade 2 (Terhorst et al., 2017): 19 eastern Chinese landraces, 14 northwestern Chinese weedy rice samples, 44 northeastern Chinese weedy rice samples, and 23 European weedy rice samples (Supplemental Data 7).

Single-population analyses showed that weedy rice experienced a pronounced contraction in effective population size (N_e) between 30 000 and 1 000 years ago, declining to approximately 2 000 individuals (Supplemental Figure 9A). This was followed by a rapid population expansion during the past 1 000 years, reaching an N_e of more than 18 000 (Supplemental Figure 9A). A similar demographic pattern was observed in landraces (Supplemental Figure 9A). Divergence time estimates indicated that northwestern Chinese weedy rice diverged from eastern Chinese landraces around 2 310 years before present (BP), representing the earliest divergence among the analyzed weedy rice groups. Subsequently (Figure 5A), northeastern Chinese weedy rice diverged from the same landrace population approximately 1 747 years BP (Figure 5B). European weedy rice, which is most closely related to northwestern Chinese landraces and weedy rice, diverged approximately 1 519 years BP (Figure 5C).

Population genetic differentiation (F_{ST}) analyses revealed substantial genetic divergence between eastern Chinese landraces and each of the three weedy rice groups. Differentiation is greatest between landraces and northwestern Chinese weedy rice ($F_{ST\ (land\text{-}E\ vs\ weed\text{-}NW)}$ = 0.347), which is higher than that observed between landraces and northeastern Chinese weedy rice ($F_{ST\ (land\text{-}E\ vs.\ weed\text{-}NE)}$ = 0.290), indicating an earlier divergence of the northwestern group (Figure 5D and Supplemental Figures 10A and 10B). In contrast, the much lower differentiation between northwestern Chinese and European weedy rice ($F_{ST\ (weed\text{-}NW\ vs.\ weed\text{-}EU)}$ = 0.126; Figure 5D and Supplemental Figure 10C) indicates a more recent divergence and closer

genetic relationship, consistent with the demographic patterns inferred from N_e trajectories (Figure 5C). The substantial differentiation between eastern Chinese landraces and European weedy rice ($F_{ST\ (land\text{-}E\ vs.}\ F_{ST\ weed\text{-}EU)}$ = 0.273; Figure 5D) further highlights their distinct evolutionary histories.

Nucleotide diversity (π) analyses revealed low diversity in eastern Chinese landraces and northwestern Chinese weedy rice ($\pi_{land\text{-}E} = 0.57 \times 10^{-3}$; $\pi_{weed\text{-}NW} = 0.49 \times 10^{-3}$; Figure 5D), whereas higher diversity was observed in European and northeastern Chinese weedy rice ($\pi_{weed\text{-}EU} = 0.80 \times 10^{-3}$; $\pi_{weed\text{-}NE} = 0.70 \times 10^{-3}$). The elevated diversity in these populations likely reflects introgression from cultivated rice, particularly in regions with a long history of intensive rice cultivation.

In conclusion, our findings support a dispersal scenario in which most European weedy rice and its ancestral populations trace back to the middle and lower Yangtze River, a primary center of rice domestication (Jing et al., 2023; Guo et al., 2025). The ancestral gene pool likely spread to northwestern and northeastern China and was subsequently introduced to Europe via the Hexi Corridor (Supplemental Figure 9B). The well-documented role of the Silk Road in facilitating agricultural exchange (Zhou et al., 2020; Kang et al., 2021; Huang et al., 2023; Gutaker and Purugganan, 2024) is consistent with the possibility that *japonica*-type landraces and associated weedy rice dispersed along this route, ultimately contributing to the establishment of most European weedy rice populations.

DISCUSSION

Our genomic analysis of weedy rice populations provides strong genetic evidence supporting an early introduction of rice into Europe from northwestern China, prior to modern breeding efforts in Europe. Earlier studies based on cultivated varieties suggested a northeastern origin for European rice (Cai et al., 2013). However, these patterns likely reflect historical breeding interventions, including the introduction of blast-resistant cultivars from northern China and Japan following the 18th-century blast epidemic, as well as extensive 20th-century germplasm exchange (Faivre-Rampant et al., 2011). Such breeding-driven admixture complicates attempts to infer older dispersal histories from modern cultivars. In contrast, European weedy rice, which largely derives from historical escape events and has been minimally affected by modern breeding (Supplemental Note 3), retains clearer ancestral genetic signals. A similarly limited impact of modern breeding is also observed in northwestern Chinese weedy rice (Supplemental Figure 11; Supplemental Note 3). We observed distinct clustering of European, northeastern, and northwestern weedy rice, with limited gene flow among regions (Figure 1C). Rare cross-regional introductions, as well as cultivar introgression and escape events, were readily detected through phylogenetic and IBD analyses and excluded from demographic inference (Figures 1C, 4B, 4D, and 4G). The strong genetic affinity between European and northwestern Chinese weedy rice therefore reflects shared ancestry rather than recent admixture, consistent with a westward introduction of *japonica* rice along the Silk Road. Because selection during de-domestication targets genomic regions largely distinct from domestication loci (Qiu et al., 2020), weedy rice may retain ancestral domestication alleles, making it a valuable proxy for reconstructing ancient crop dispersal processes.

Successful crop dispersal depends not only on human-mediated movement but also on the ability of crops to adapt to new environments. Among the three hypotheses proposed for the origin of rice in Europe (Spengler et al., 2021; Muthukumaran, 2023; Ren et al., 2024), our results do not exclude the possibility that southern routes through South Asia contributed to its introduction. However, rice varieties from low-latitude regions, such as Yunnan in China or India, generally lack the adaptations required to tolerate Europe's long-day photoperiods and cooler end-of-season temperatures. Our analysis of photoperiod-related alleles supports this view: both European cultivars and weedy rice carry the same flowering-time genotypes as landraces and weedy rice from northwestern China, a region located at a similar latitude. This genetic correspondence provides further evidence for a contribution from northwestern Chinese populations to early European rice. Even if rice was introduced via alternative routes, varieties originating from lower-latitude regions would have been less likely to establish successfully because of their limited adaptation to long-day and cooler conditions. The predominance of *japonica* rice in Europe, accounting for approximately 90% of cultivated rice, further underscores the importance of environmental adaptation, as *indica* varieties from South Asia are generally more sensitive to cold stress (Courtois et al., 2012). More broadly, domesticated crops often exhibit narrower environmental adaptability, reflecting adaptations to specific ecological conditions (Zhang et al., 2015; Dong et al., 2021; Zhou et al., 2021; Li et al., 2024b). Consequently, crop introductions are more likely to succeed when source populations share similar ecological conditions with the new habitat. In this context, adaptive alleles—such as those involved in photoperiod responses—can serve as valuable markers for reconstructing the history of crop dispersal from the perspective of environmental adaptation.

Establishing the early escape of weedy rice in Europe is essential for using it as a tool to understand rice dispersal history. Our phylogenetic analysis, PCA, and population structure results show that most European weedy rice accessions are distinct from modern European cultivars and are more closely related to northwestern Chinese weedy rice and landraces. Although the number of samples from northwestern China is limited (Supplemental Note 4), the phylogenetic patterns, allele distributions, and inferred demographic history are highly consistent, supporting the robustness and reliability of our conclusions. These findings suggest a shared ancestry that predates modern rice breeding in Europe. The presence of shared photoperiod alleles further supports this relationship and highlights their roles in facilitating adaptation to the European environment. We estimate that northwestern and European weedy rice diverged approximately 1500 years ago, indicating that the introduction of rice into Europe likely occurred at or before this time. This period coincides with the Southern and Northern Dynasties in China, when trade along the Silk Road flourished, surpassing that of earlier periods, including the Han dynasty (Fang et al., 2024). Historical records indicate that overland trade routes to the Eastern Roman Empire—referred to as Daqin (大秦) or Pulan (普岚) in Chinese sources—were established during the Northern Wei period and facilitated frequent exchanges (Fang et al., 2024). Supporting this historical context, *japonica*-type rice grains from the 4th century CE have been discovered in Xinjiang, with phytolith evidence dating back to as early as the 4th century BCE (Spengler et al., 2021; Ren et al., 2024). The oldest rice seeds

discovered in Xinjiang date to approximately 3000 years ago (Heywood et al., 2024), suggesting a long history of rice cultivation in northwestern China and its potential role in westward dissemination. In Europe, the earliest archaeological evidence of rice dates to the 1st century CE (Spengler et al., 2021). In addition, *japonica*-type rice grains have been reported in Iran (120 BCE), Tajikistan (91–109 BCE), and Uzbekistan (236–386 CE)—key hubs along the Silk Road (Spengler et al., 2021; Huang et al., 2023). These archaeological records correspond closely with the period during which the overland Silk Road emerged and flourished. By integrating historical records, archaeological findings, and genomic evidence, we propose that the progenitors of most European weedy rice were likely introduced from northwestern China via the Silk Road during or before this period. Furthermore, our data indicate that eastern Chinese landraces diverged from northwestern and northeastern Chinese weedy rice approximately 2310 and 1747 years ago, respectively. This pattern is consistent with the hypothesis that Asian *japonica* rice originated from ancient landraces in eastern China (Huang et al., 2012; Zhang et al., 2024; Guo et al., 2025), subsequently spreading westward and ultimately reaching Europe via the Silk Road (Ren et al., 2024), thereby contributing to the genetic makeup of European rice populations.

Our findings also reveal that European weedy rice has a mixed origin, involving ancient introduction events, introgression from modern cultivars, and recent *de novo* feralization. Sun et al. showed that northeastern Chinese weedy rice originated from semi-domesticated landraces (Sun et al., 2019). In our phylogenetic tree, northeastern weedy rice clusters with northwestern and most European weedy rice within the same clade (clade 2), suggesting a shared ancestry distinct from modern cultivars. These lineages may therefore represent early escape events from ancient northwestern landraces. Meanwhile, Qiu et al. (2020) reported that *indica*-type weedy rice in eastern China originated from elite cultivars bred in the 1960s. Consistent with this pattern, we identified individuals in the Italian weedy rice population that likely arose from recent escape events. Phylogenetic analysis suggests that the Italian cultivar Gladio, a long-B-grain-type rice released in 1998 and widely cultivated thereafter (Lupotto et al., 2008), may be the direct ancestor of Wr_Italy20. Because long B-grain types in Italy were developed using American germplasm in the 1990s (Faivre-Rampant et al., 2011), it is plausible that Wr_Italy20 emerged within the past three decades. Additionally, several Italian weedy rice individuals harbor introgressed genomic fragments from modern European cultivars, consistent with previous reports from this region (Jiang et al., 2012). Notably, these introgressed segments do not overlap among individuals but instead display a mosaic-like distribution, suggesting that gene flow from cultivated rice has occurred but that these fragments have not yet reached fixation. This observation also has practical implications: during large-scale cultivation of genetically modified rice, such gene flow could facilitate the spread of transgenes—such as herbicide-tolerant or insect-resistant traits—into weedy rice populations.

One intriguing but unresolved question is whether European weedy rice in clade 2 originated from ancient northwestern cultivars that escaped after their introduction into Europe, or whether it had already evolved as a weedy form before dispersal. Because modern European cultivars have undergone extensive breeding, divergence time estimates between cultivars and weedy rice are unreliable. However, phylogenetic analysis of the *Rc* gene, which controls red pericarp (Sweeney et al., 2006; Grimm et al., 2020), provides a potential clue. Red pericarp in weedy rice can arise through spontaneous back mutations, gene flow from wild relatives, or standing variation inherited from ancient red-pericarp rice (Xie et al., 2024). In our dataset, European weedy rice harbors two major *Rc* haplotypes. One carries the typical 14-bp frameshift deletion found in most cultivars, together with an upstream 1-bp deletion that may restore pigmentation (Grimm et al., 2020). The other lacks the 14-bp deletion entirely, representing a likely ancestral haplotype closely related to wild rice (Supplemental Figure 12). This latter allele is also present in northwestern Chinese weedy rice, two landraces from the same region, and one Portuguese cultivar. These observations suggest that red pericarp in European weedy rice may derive from standing variation in ancient landraces, through two possible routes: pigmented cultivars were introduced and later gave rise to weedy rice through escape, or weedy rice carrying this haplotype was introduced from northwestern China and later cultivated as a pigmented variety. Considering that independent de-domestication of ancient cultivars in Europe would likely have resulted in genomic divergence from northwestern Chinese weedy rice, the closer genetic affinity of European weedy rice to northwestern Chinese weedy rice—at the *Rc* gene, flowering-time loci, and genome-wide level—suggests that the second scenario is more likely, namely that weedy rice from northwestern China was introduced directly into Europe. Although our findings support this interpretation, broader sampling and analysis of additional de-domestication loci will be required to further clarify these scenarios.

In summary, our results provide genetic evidence consistent with the westward spread of rice along the overland Silk Road, suggesting that northwestern China contributed substantially to the ancestry of early European rice populations and highlighting the potential role of ancient trade routes in crop dispersal. These findings also underscore the unique value of weedy rice in preserving genetic signatures of ancient rice, offering important insights into the historical spread of this crop. Environmentally adaptive alleles, such as those involved in photoperiod sensitivity, can serve as markers of ancient populations, shedding light on the timing and geographic origins of dispersal while also representing potential resources for crop improvement. Additionally, the diverse evolutionary trajectories of European weedy rice revealed in this study have important implications for managing weedy rice populations and mitigating transgene escape from genetically modified crops. Finally, as with most evolutionary reconstructions, our conclusions are limited by current sampling and model assumptions. Although the genomic patterns presented here are consistent with a westward dispersal scenario, alternative histories cannot be completely excluded and will require broader sampling and future integrative analyses.

METHODS

Sample collection, phenotyping, and sequencing

We collected 23 weedy rice samples from 20 regions across 13 provinces in China, all displaying typical weedy rice morphological characteristics (Supplemental Data 1; Supplemental Figure 1). These samples were grown under natural long-day field conditions in Nanjing, Jiangsu Province

(32.12°N, 118.96°E) during the 2022 and 2024 growing seasons. In 2024, we additionally cultivated Asian cultivated and weedy rice lines carrying *Hd1* haplotypes commonly observed in European cultivars, allowing direct comparison of their flowering times with weedy rice lines harboring the $Hd1^{EH}$ allele under summer field conditions in Nanjing.

To evaluate the effect of the deletion-type *Hd4* allele, we generated near-isogenic lines by crossing the maternal inbred *O. sativa* ssp. *indica* cv. PA64s, a photo-thermosensitive male sterile line carrying the functional $Hd4^{NIP}$ allele, with the paternal inbred *O. sativa* ssp. *indica* cv. Zhongjiazao 17, which carries the deletion-type $Hd4^{Digu}$ allele. The progeny were advanced to the F_6 generation. From this segregating population, we selected homozygous individuals with a uniform genetic background (functional *Hd1* and *RFT1*, and nonfunctional *Hd2* and *Hd5*) that differed only at the *Hd4* locus. The resulting plants were grown in 2024.

All rice accessions used in this study were sown in mid-May, and heading dates were recorded at 3-day intervals starting in July. After excluding edge plants, at least 15 individuals per accession were retained for phenotypic analyses. To evaluate the potential influence of latitude on allelic effects, we further compared our field observations with multi-site European flowering-time datasets reported by Gómez-Ariza et al. (2015), thereby assessing the robustness of our conclusions across different environments (Supplemental Note 1).

Genomic DNA was extracted from leaf tissue using the cetyltrimethylammonium bromide method (Clarke, 2009). DNA samples were then sent to the Beijing Genome Institute (BGI, Beijing, China) for quality assessment and library preparation. Paired-end sequencing was performed on the DNBSEQ-T7 platform with a read length of 150 bp.

In addition, we incorporated previously published high-quality genomic datasets of weedy and cultivated rice from Asia and Europe, including 173 weedy rice genomes, the genomes of 355 *japonica* cultivars (37 landraces and 318 modern cultivars), 15 *aromatic* rice genomes, and 16 *aus* rice genomes (Xu et al., 2012; Qiu et al., 2017, 2020; Wang et al., 2018; Mao et al., 2019; Li et al., 2020; Han et al., 2022; Ye et al., 2022; Cao et al., 2023). The final dataset comprised 582 rice samples, including 196 weedy rice and 386 cultivated rice accessions (Supplemental Data 1 and 2). The geographic distribution of rice samples across Eurasia was visualized in R using the *ggplot2* package (Gómez-Rubio, 2017).

Sequencing data processing and variant calling

Raw sequencing reads were filtered using SOAPnuke to remove adaptor sequences and low-quality reads (Chen et al., 2018). Reads containing more than 0.1% N bases or with >50% of bases having a quality score below 10 were excluded. The filtered clean reads were then aligned to the Nipponbare reference genome (IRGSP-1.0) (Kawahara et al., 2013) using BWA-MEM (v0.7.10-r789) (Li, 2013). The resulting SAM files were sorted, and PCR duplicates were removed using Picard tools (https://broadinstitute.github.io/picard/, v1.114, accessed October 18, 2020). To improve the accuracy of variant calling, regions surrounding insertions or deletions (indels) were realigned using GATK (v3.7) (McKenna et al., 2010).

Variant calling was performed using GATK. Specifically, HaplotypeCaller was used to generate GVCF files for each sample, followed by joint genotyping with GenotypeGVCFs (-stand_call_conf 30.0). To further reduce false-positive variants, we applied filtering criteria commonly used in previous rice genomic studies (Wang et al., 2018, 2023). Specifically, VCFtools was used with the following parameters: –max-missing 0.9 –minDP 4 –maxDP 1000 –minQ 60 –minGQ 0 –min-alleles 2 –max-alleles 2 –remove-indels. After filtering, 6 715 815 high-quality SNPs were retained for downstream analyses and designated as the 6.71M-SNP dataset. This dataset was subsequently used for phylogenetic analysis, PCA, and population structure analysis to determine the phylogenetic status of European weedy rice.

Phylogenetic and population structure analyses

A maximum-likelihood phylogenetic tree was constructed for the 582 rice accessions based on the 6.71M SNP dataset using FastTree (v2.1.11, double precision) with the Jukes-Cantor (JC) model and 1000 bootstrap replicates (Price et al., 2009). The phylogenetic tree was visualized using iTOL (https://itol.embl.de/) (Letunic and Bork, 2024). PCA of *japonica* rice was performed using GCTA (v1.94.1) based on the 6.71M SNP dataset (Yang et al., 2011), and the results were visualized using a Python script. Population structure of *japonica* rice was inferred using ADMIXTURE (v1.3.0) to estimate ancestry proportions and categorize populations (Alexander et al., 2009). The number of ancestral populations (K) was tested from 2 to 15. The same approach was applied to examine population structure among modern-EU, weed-EU, and weed-NW to investigate the origin of European weedy rice and potential gene introgression events.

Screening for conserved genomic regions between European rice and northwestern weedy rice

Conserved genomic regions were identified based on sequence divergence between pairs of samples from different populations. To minimize the impact of genetic introgression from recent breeding, population bottlenecks, and conserved regions shared among *japonica* rice populations, we applied the following strategy for conserved-region identification.

First, the genome was divided into 20-kb windows, and the differential SNP ratio between sample pairs of European weedy rice and European cultivated rice was calculated using a Perl script (see data and code availability). The distribution of differential SNP ratios within each window was then evaluated, and conserved regions were defined using a third-quartile threshold of <0.1, ensuring that the selected genomic regions were conserved across most individuals. Considering potential bottleneck effects during the dispersal process, a first-quartile threshold of <0.1 was used to identify conserved regions between European weedy rice and northwestern Chinese weedy rice. The intersection of conserved regions identified in these two comparisons was then selected as candidate conserved regions shared by European rice and northwestern Chinese weedy rice. To eliminate potential false positives arising from conserved regions common within *japonica* rice populations, we removed regions that were also conserved between European weedy rice and Asian modern cultivated rice. Finally, the chromosomal distribution of conserved regions was visualized using the *RIdeogram* package in R (Hao et al., 2020).

Genotype analysis of photoperiod-related genes

SNPs and indels located within the gene body and the 3,000-bp flanking regions of candidate genes in *japonica* rice samples were identified using GATK. Variant effects were predicted using SNPeffect (v4.3) (De Baets et al., 2012). The Integrative Genomics Viewer was used to inspect BAM files for each sample to detect large segmental tandem duplications (Thorvaldsdottir et al., 2013), transposon insertions, and other structural variations that might have been missed in the VCF files. Haplotypes in the coding regions, non-coding regions, and flanking regions of candidate genes were identified and visualized using the R packages *quickHapR* (https://github.com/ZhangRenL/quickHapR) and *pheatmap* (v1.0.12). The $Hd1^{EH}$ transposon insertion and the $Hd4^{NW}$ large-fragment deletion were further validated by PCR cloning. Primers used for PCR cloning are listed in Supplemental Table 3.

Population genetic analysis

Rice samples were categorized into populations based on their geographic origin and their positions in the phylogenetic tree (see Supplemental Data 1 and 2). To reduce the impacts of overly strict filtering criteria and noise introduced by rare variants, population genetic diversity and

inter-population variation were analyzed using SNPs filtered with a missing rate of less than 20% and a minor allele frequency of less than 0.05 (Wang et al., 2018; Ye et al., 2022). After filtering, a dataset containing 2 521 894 SNPs was obtained and designated the 2.5M-SNP dataset, which was used for subsequent population genetic analyses. Nucleotide diversity (π) within populations was calculated using VCFtools with a 20-kb window size and a 5-kb step size across the genome (Danecek et al., 2011). The fixation index (F_{ST}) was also computed using VCFtools with the same sliding-window parameters (20-kb window and 5-kb step). The results were visualized as Manhattan plots using the *qqman* (v0.1.9) (Turner, 2018) and *Cairo* (v1.6-2; https://github.com/s-u/Cairo) packages in R.

IBD fragment detection and gene introgression analysis

To investigate genome-wide introgression between European weedy rice and European cultivated rice, we used refined-IBD (v17Jan20.102) to identify haplotypes in European weedy rice accessions that were identical by descent (IBD) with individuals from each population (Browning and Browning, 2013). Prior to IBD detection, to improve the resolution of population structure and minimize redundant information, a linkage disequilibrium-pruned SNP set containing 61 858 SNPs was generated from the 2.5M-SNP dataset using PLINK (v1.90b6.21) with the parameters 10 SNPs per 50-kb window and $r^2 < 0.2$ (Purcell et al., 2007). Genotype imputation and phasing were conducted with BEAGLE (v5.0) using default parameters based on genotype likelihoods (Browning and Browning, 2016). IBD sharing between an individual and a group was defined as the union of IBD segments shared between the individual and each member of the group. We first measured the IBD fragments shared between each individual in clade 1 and the northwestern Chinese and European weedy rice accessions in clade 2, which were treated as ancestral haplotypes. Individuals in clade 1 were then categorized according to whether they carried $Hd1^{EH}$ or $Hd4^{NW}$, as well as by their classification as weedy or cultivated rice. A two-sided *t*-test was performed to evaluate significant differences between groups. The same approach was used to identify IBD segments shared between weed-EU and modern-EU populations.

For Wr_Italy20 and Wr_Italy27, we extracted two SNP datasets: (i) SNPs located within IBD regions shared with European cultivated rice, and (ii) SNPs from genomic regions excluding IBD segments shared with any other group. Phylogenetic trees were constructed using these two SNP datasets for representative individuals selected from the modern-EU, weed-EU, weed-NW, weed-NE, and *aromatic* groups to examine the origins of the IBD regions and the genetic backgrounds of the two European weedy rice accessions.

Estimation of divergence time and demographic history

To infer the timing of rice dispersal from northwestern China to Europe, we used SMC^{++} to estimate population size histories and split times between northwestern Chinese weedy rice and European weedy rice (Terhorst et al., 2017). All SNPs were included in the inference of effective population size (N_e) history. Based on the phylogenetic tree, individuals located on the main clades were selected as representative samples. These representatives included 19 eastern Chinese landraces, 14 northwestern Chinese weedy rice samples, 44 northeastern Chinese weedy rice samples, and 23 European weedy rice samples (Supplemental Data 7). A mutation rate of 6.5×10^{-9} substitutions per synonymous site per generation and a generation time of 1 year (reflecting the annual life cycle of weedy rice) (Chandra Roy et al., 2023) were specified in the SMC^{++} *estimate* function, with all other parameters set to default following previous studies (Qiu et al., 2020). The mutation rate was derived from synonymous substitution rates estimated for the conserved *Adh1* and *Adh2* genes in grasses, whose synonymous sites are generally considered largely neutral (Gaut et al., 1996). Divergence times among geographically distinct rice populations were subsequently inferred using the SMC^{++} *split* function (Terhorst et al., 2017).

DATA AND CODE AVAILABILITY

The complete DNA sequencing data generated in this study have been deposited in the NCBI Sequence Read Archive (SRA) under accession number PRJNA1290498. The custom scripts used in this study are available at: https://git.nju.edu.cn/Zhongyf/Codes-for-The-spread-of-weed-rice-between-China-and-Europe.git.

FUNDING

This work was supported by the National Natural Science Foundation of China (32170327, 32470235, and 32270664), the Natural Science Foundation of Jiangsu Province for Distinguished Young Scholars (BK20240064), and the Fundamental Research Funds for the Central Universities (14380209).

ACKNOWLEDGMENTS

The authors thank the National Mid-term Genebank for Rice of the China National Rice Research Institute for providing the rice germplasm collection. No conflict of interest declared.

AUTHOR CONTRIBUTIONS

Conceptualization and design of the research: S.Y., L.W., J.H., and Q.Z.; sample collection, extraction, and processing: Y.Z., W.C., and S.X.; data processing and analyses: S.Y., L.W., Y.Z., and Y.W.; molecular experiments, including PCR cloning: W.C., S.X., and X.Z.; manuscript drafting and figure preparation: S.Y., L.W., and Y.Z. All authors commented on the manuscript. Y.Z. and W.C. contributed equally to this work.

DECLARATION OF GENERATIVE AI AND AI-ASSISTED TECHNOLOGIES IN THE WRITING PROCESS

During the preparation of this work, the authors used ChatGPT to improve the English language. After using this tool, the authors reviewed and edited the content as necessary and take full responsibility for the content of the published article.

SUPPLEMENTAL INFORMATION

Supplemental information is available at *Plant Communications Online*.

Received: July 15, 2025
Revised: December 15, 2025
Accepted: March 5, 2026
Published: March 10, 2026

REFERENCES

Alexander, D.H., Novembre, J., and Lange, K. (2009). Fast model-based estimation of ancestry in unrelated individuals. Genome Res. **19**:1655–1664.

Browning, B.L., and Browning, S.R. (2013). Improving the Accuracy and Efficiency of Identity-by-Descent Detection in Population Data. Genetics **194**:459–471.

Browning, B.L., and Browning, S.R. (2016). Genotype Imputation with millions of reference samples. Am. J. Hum. Genet. **98**:116–126.

Cai, X., Fan, J., Jiang, Z., Basso, B., Sala, F., Spada, A., Grassi, F., and Lu, B.-R. (2013). The Puzzle of Italian Rice Origin and Evolution: Determining Genetic Divergence and Affinity of Rice Germplasm from Italy and Asia. PLoS One **8**:e80351.

Cao, S., Chen, K., Lu, K., Chen, S., Zhang, X., Shen, C., Zhu, S., Niu, Y., Fan, L., Chen, Z.J., et al. (2023). Asymmetric variation in DNA methylation during domestication and de-domestication of rice. Plant Cell **35**:3429–3443. https://doi.org/10.1093/plcell/koad160.

Chandra Roy, S., Singh, L., and Sarkar, S. (2023). Agro-morphological Characteristics of Weedy Rice (Oryza sativa f. spontanea): A Precious Genetic Resource for Rice Improvement. Curr. Agri. Res. Jour. **11**:450–467.

Chen, Y., Chen, Y., Shi, C., Huang, Z., Zhang, Y., Li, S., Li, Y., Ye, J., Yu, C., Li, Z., et al. (2018). SOAPnuke: a MapReduce acceleration-supported software for integrated quality control and preprocessing of high-throughput sequencing data. GigaScience **7**:1–6.

Choi, J.Y., Platts, A.E., Fuller, D.Q., Hsing, Y.-I., Wing, R.A., and Purugganan, M.D. (2017). The rice paradox: Multiple origins but single domestication in Asian rice. Mol. Biol. Evol. **34**:969–979. https://doi.org/10.1093/molbev/msx049.

Civáň, P., Craig, H., Cox, C.J., and Brown, T.A. (2015). Three geographically separate domestications of Asian rice. Nat. Plants **1**:15164.

Clarke, J.D. (2009). Cetyltrimethyl ammonium bromide (CTAB) DNA miniprep for plant DNA isolation. Cold Spring Harb. Protoc. **2009**:pdb.prot5177.

Courtois, B., Frouin, J., Greco, R., Bruschi, G., Droc, G., Hamelin, C., Ruiz, M., Clément, G., Evrard, J., Van Coppenole, S., et al. (2012). Genetic Diversity and Population Structure in a European Collection of Rice. Crop Sci. **52**:1663–1675.

Cui, Y., Wang, J., Feng, L., Liu, S., Li, J., Qiao, W., Song, Y., Zhang, Z., Cheng, Y., Zhang, L., et al. (2020). A Combination of Long-Day Suppressor Genes Contributes to the Northward Expansion of Rice. Front. Plant Sci. **11**:864.

D. Turner, S. (2018). qqman: an R package for visualizing GWAS results using Q-Q and manhattan plots. JOSS **3**:731.

Danecek, P., Auton, A., Abecasis, G., Albers, C.A., Banks, E., DePristo, M.A., Handsaker, R.E., Lunter, G., Marth, G.T., Sherry, S.T., et al. (2011). The variant call format and VCFtools. Bioinformatics **27**:2156–2158.

De Baets, G., Van Durme, J., Reumers, J., Maurer-Stroh, S., Vanhee, P., Dopazo, J., Schymkowitz, J., and Rousseau, F. (2012). SNPeffect 4.0: on-line prediction of molecular and structural effects of protein-coding variants. Nucleic Acids Res. **40**:D935–D939.

Dong, L., Fang, C., Cheng, Q., Su, T., Kou, K., Kong, L., Zhang, C., Li, H., Hou, Z., Zhang, Y., et al. (2021). Genetic basis and adaptation trajectory of soybean from its temperate origin to tropics. Nat. Commun. **12**:5445.

Eshed, Y., and Lippman, Z.B. (2019). Revolutions in agriculture chart a course for targeted breeding of old and new crops. Science **366**:eaax0025.

Faivre-Rampant, O., Bruschi, G., Abbruscato, P., Cavigiolo, S., Picco, A.M., Borgo, L., Lupotto, E., and Piffanelli, P. (2011). Assessment of genetic diversity in Italian rice germplasm related to agronomic traits and blast resistance (Magnaporthe oryzae). Mol. Breeding **27**:233–246.

Fang, G., Xu, K., and Hu, S. (2024). Historical status of the silk road in the Northern and Southern dynasties of the Wei and Jin dynasties (in Chinese). Chin. Econ. History Rev. **2**:22–24.

Fujino, K., Yamanouchi, U., and Yano, M. (2013). Roles of the Hd5 gene controlling heading date for adaptation to the northern limits of rice cultivation. Theor. Appl. Genet. **126**:611–618.

Gaut, B.S., Morton, B.R., McCaig, B.C., and Clegg, M.T. (1996). Substitution rate comparisons between grasses and palms: synonymous rate differences at the nuclear gene Adh parallel rate differences at the plastid gene rbcL. Proc. Natl. Acad. Sci. USA **93**:10274–10279.

Gómez-Ariza, J., Galbiati, F., Goretti, D., Brambilla, V., Shrestha, R., Pappolla, A., Courtois, B., and Fornara, F. (2015). Loss of floral repressor function adapts rice to higher latitudes in Europe. J. Exp. Bot. **66**:2027–2039.

Gómez-Rubio, V. (2017). ggplot2 - Elegant Graphics for Data Analysis (2nd Edition) (J. Stat. Soft.), p. 77.

Goretti, D., Martignago, D., Landini, M., Brambilla, V., Gómez-Ariza, J., Gnesutta, N., Galbiati, F., Collani, S., Takagi, H., Terauchi, R., et al. (2017). Transcriptional and Post-transcriptional Mechanisms Limit Heading Date 1 (Hd1) Function to Adapt Rice to High Latitudes. PLoS Genet. **13**:e1006530.

Grimm, A., Fogliatto, S., Nick, P., Ferrero, A., Vidotto, F., et al. (2013). Microsatellite markers reveal multiple origins for Italian weedy rice. Ecol. Evol **3**:4786–4798.

Grimm, A., Sahi, V.P., Amann, M., Vidotto, F., Fogliatto, S., Devos, K.M., Ferrero, A., and Nick, P. (2020). Italian weedy rice—A case of de-domestication? Ecol. Evol. **10**:8449–8464.

Guo, D., Li, Y., Lu, H., Zhao, Y., Kurata, N., Wei, X., Wang, A., Wang, Y., Zhan, Q., Fan, D., et al. (2025). A pangenome reference of wild and cultivated rice. Nature **642**:662–671.

Gutaker, R.M., and Purugganan, M.D. (2024). Adaptation and the Geographic Spread of Crop Species. Annu. Rev. Plant Biol. **75**:679–706.

Han, B., Cui, D., Ma, X., Cao, G., Zhang, H., Koh, H.J., and Han, L. (2022). Evidence for evolution and selection of drought-resistant genes based on high-throughput resequencing in weedy rice. J. Exp. Bot. **73**:1949–1962.

Hao, Z., Lv, D., Ge, Y., Shi, J., Weijers, D., Yu, G., and Chen, J. (2020). *RIdeogram* : drawing SVG graphics to visualize and map genome-wide data on the idiograms. PeerJ Comput. Sci. **6**:e251.

Herath, V. (2019). The architecture of the GhD7 promoter reveals the roles of GhD7 in growth, development and the abiotic stress response in rice. Comput. Biol. Chem. **82**:1–8.

Heywood, S., Spate, M., Betts, A., Jia, P., and Fairbairn, A. (2024). Late Bronze Age agriculture and the early westward transmission of rice at Luanzagangzi, Northern Xinjiang, China. Holocene **34**:202–211.

Huang, X., Kurata, N., Wei, X., Wang, Z.-X., Wang, A., Zhao, Q., Zhao, Y., Liu, K., Lu, H., Li, W., et al. (2012). A map of rice genome variation reveals the origin of cultivated rice. Nature **490**:497–501.

Huang, Y., Deng, Z., Nashli, H.F., Fuller, D.Q., Wu, X., and Safari, M. (2023). The early adoption of East Asian crops in West Asia: rice and broomcorn millet in northern Iran. Antiquity **97**:674–689.

Jiang, Z., Xia, H., Basso, B., and Lu, B.-R. (2012). Introgression from cultivated rice influences genetic differentiation of weedy rice populations at a local spatial scale. Theor. Appl. Genet. **124**:309–322.

Jing, C.-Y., Zhang, F.-M., Wang, X.-H., Wang, M.-X., Zhou, L., Cai, Z., Han, J.-D., Geng, M.-F., Yu, W.-H., Jiao, Z.-H., et al. (2023). Multiple domestications of Asian rice. Nat. Plants **9**:1221–1235.

Kang, L., Qian, L., Zheng, M., Chen, L., Chen, H., Yang, L., You, L., Yang, B., Yan, M., Gu, Y., et al. (2021). Genomic insights into the origin, domestication and diversification of Brassica juncea. Nat. Genet. **53**:1392–1402.

Kawahara, Y., De La Bastide, M., Hamilton, J.P., Kanamori, H., McCombie, W.R., Ouyang, S., Schwartz, D.C., Tanaka, T., Wu, J., Zhou, S., et al. (2013). Improvement of the Oryza sativa Nipponbare reference genome using next generation sequence and optical map data. Rice **6**:4.

Kim, S.-R., Torollo, G., Yoon, M.-R., Kwak, J., Lee, C.-K., Prahalada, G.D., Choi, I.-R., Yeo, U.-S., Jeong, O.-Y., Jena, K.K., and Lee, J.S. (2018). Loss-of-Function Alleles of Heading date 1 (Hd1) Are Associated With Adaptation of Temperate Japonica Rice Plants to the Tropical Region. Front. Plant Sci. **9**:1827.

Koo, B.-H., Yoo, S.-C., Park, J.-W., Kwon, C.-T., Lee, B.-D., An, G., Zhang, Z., Li, J., Li, Z., and Paek, N.-C. (2013). Natural Variation in OsPRR37 Regulates Heading Date and Contributes to Rice Cultivation at a Wide Range of Latitudes. Mol. Plant **6**:1877–1888.

Letunic, I., and Bork, P. (2024). Interactive Tree of Life (iTOL) v6: recent updates to the phylogenetic tree display and annotation tool. Nucleic Acids Res. **52**:W78–W82.

Li, H. (2013). Aligning sequence reads, clone sequences and assembly contigs with BWA-MEM. arXiv. https://doi.org/10.48550/arXiv.1303.3997.

Li, X., Chen, Z., Zhang, G., Lu, H., Qin, P., Qi, M., Yu, Y., Jiao, B., Zhao, X., Gao, Q., et al. (2020). Analysis of genetic architecture and favorable allele usage of agronomic traits in a large collection of Chinese rice accessions. Sci. China Life Sci. **63**:1688–1702.

Li, Z., Gui, R., Yu, X., Liang, C., Cui, J., Zhao, X., Zhang, X., Yu, P., Chen, W., and Sun, J. (2022). Genetic basis of the early heading of high-latitude weedy rice. Front. Plant Sci. **13**:1059197.

Li, L.-F., Li, Y.-L., Jia, Y., Caicedo, A.L., and Olsen, K.M. (2017). Signatures of adaptation in the weedy rice genome. Nat. Genet. **49**:811–814.

Li, L.-F., Pusadee, T., Wedger, M.J., Li, Y.-L., Li, M.-R., Lau, Y.-L., Yap, S.-J., Jamjod, S., Rerkasem, B., Hao, Y., et al. (2024a). Porous borders at the wild-crop interface promote weed adaptation in Southeast Asia. Nat. Commun. **15**:1182.

Li, J., Li, Y., Agyenim-Boateng, K.G., Shaibu, A.S., Liu, Y., Feng, Y., Qi, J., Li, B., Zhang, S., and Sun, J. (2024b). Natural variation of domestication-related genes contributed to latitudinal expansion and adaptation in soybean. BMC Plant Biol. **24**:651.

Lin, X., Fang, C., Liu, B., and Kong, F. (2021). Natural variation and artificial selection of photoperiodic flowering genes and their applications in crop adaptation. aBIOTECH **2**:156–169.

Londo, J.P., Chiang, Y.-C., Hung, K.-H., Chiang, T.-Y., and Schaal, B.A. (2006). Phylogeography of Asian wild rice, *Oryza rufipogon* , reveals multiple independent domestications of cultivated rice, *Oryza sativa*. Proc. Natl. Acad. Sci. USA **103**:9578–9583.

Lou, G., Chen, P., Li, P., Gao, H., Xiong, J., Wan, S., Zheng, Y., Wang, Y., Alam, M., Chen, Y., et al. (2025). Antagonistic Ghd7-OsNAC42 Complexes Modulate Carbon and Nitrogen Metabolism to Achieve Superior Quality and High Yield in Rice. Adv. Sci. **12**:e04163.

Lupotto, E., Cavagna, B., Corana, F., et al. (2008). Quality evaluation and high throughput analysis of aromatic Italian rice varieties through HS-SPME/GC-MS analysis. In Consumer Driven Cereal Innovation, P.L. Weegels, C.M. Courtin, A. Arrachid, and M. Silvestri, eds. (Elsevier), pp. 78–82.

Mao, D., Xin, Y., Tan, Y., Hu, X., Bai, J., Liu, Z.Y., Yu, Y., Li, L., Peng, C., Fan, T., et al. (2019). Natural variation in the *HAN1* gene confers chilling tolerance in rice and allowed adaptation to a temperate climate. Proc. Natl. Acad. Sci. USA **116**:3494–3501.

McKenna, A., Hanna, M., Banks, E., Sivachenko, A., Cibulskis, K., Kernytsky, A., Garimella, K., Altshuler, D., Gabriel, S., Daly, M., and DePristo, M.A. (2010). The Genome Analysis Toolkit: A MapReduce framework for analyzing next-generation DNA sequencing data. Genome Res. **20**:1297–1303.

Molina, J., Sikora, M., Garud, N., Flowers, J.M., Rubinstein, S., Reynolds, A., Huang, P., Jackson, S., Schaal, B.A., Bustamante, C.D., et al. (2011). Molecular evidence for a single evolutionary origin of domesticated rice. Proc. Natl. Acad. Sci. USA **108**:8351–8356.

Muthukumaran, S. (2023). Ancient crop movements from South Asia to the Middle East and the Mediterranean. Veg. Hist. Archaeobot. **35**:35–43. https://doi.org/10.1007/s00334-023-00948-8.

Nagalla, A.D., Nishide, N., Hibara, K.I., and Izawa, T. (2021). High Ambient Temperatures Inhibit Ghd7-mediated Flowering Repression in Rice. Plant Cell Physiol. **62**:1745–1759. https://doi.org/10.1093/pcp/pcab129.

Nemoto, Y., Nonoue, Y., Yano, M., and Izawa, T. (2016). *Hd1* ,a *CONSTANS* ortholog in rice, functions as an *Ehd1* repressor through interaction with monocot-specific CCT-domain protein Ghd7. Plant J. **86**:221–233.

Price, M.N., Dehal, P.S., and Arkin, A.P. (2009). FastTree: Computing Large Minimum Evolution Trees with Profiles instead of a Distance Matrix. Mol. Biol. Evol. **26**:1641–1650.

Purcell, S., Neale, B., Todd-Brown, K., Thomas, L., Ferreira, M.A.R., Bender, D., Maller, J., Sklar, P., De Bakker, P.I.W., Daly, M.J., et al. (2007). PLINK: a tool set for whole-genome association and population-based linkage analyses. Am. J. Hum. Genet. **81**:559–575.

Qiu, J., Zhou, Y., Mao, L., Ye, C., Wang, W., Zhang, J., Yu, Y., Fu, F., Wang, Y., Qian, F., et al. (2017). Genomic variation associated with local adaptation of weedy rice during de-domestication. Nat. Commun. **8**:15323.

Qiu, J., Jia, L., Wu, D., Weng, X., Chen, L., Sun, J., Chen, M., Mao, L., Jiang, B., Ye, C., et al. (2020). Diverse genetic mechanisms underlie worldwide convergent rice feralization. Genome Biol. **21**:70.

Ren, L., Li, Y., Zuo, X., Li, H., Storozum, M., Wang, X., and Jia, X. (2024). Tracing the Xinjiang Routes for the westward dispersal of rice: Phytolith analysis at the Haheren site in the southern Tianshan foothills of eastern Central Asia. J. Archaeol. Sci.: Reports **57**:104623.

Shah, S.M., Arif, M., Aslam, K., Shabir, G., and Thomson, M.J. (2016). Genetic diversity analysis of Pakistan rice (Oryza sativa) germplasm using multiplexed single nucleotide polymorphism markers. Genet. Resour. Crop Evol. **63**:1113–1126.

Shang, L., Li, X., He, H., Yuan, Q., Song, Y., Wei, Z., Lin, H., Hu, M., Zhao, F., Zhang, C., et al. (2022). A super pan-genomic landscape of rice. Cell Res. **32**:878–896.

Spada, A., Mantegazza, R., Biloni, M., Caporali, E., and Sala, F. (2004). Italian rice varieties: historical data, molecular markers and pedigrees to reveal their genetic relationships. Plant Breed. **123**:105–111.

Spengler, R.N., Stark, S., Zhou, X., Fuks, D., Tang, L., Mir-Makhamad, B., Bjørn, R., Jiang, H., Olivieri, L.M., Begmatov, A., and Boivin, N. (2021). A Journey to the West: The Ancient Dispersal of Rice Out of East Asia. Rice **14**:83.

Sun, J., Ma, D., Tang, L., Zhao, M., Zhang, G., Wang, W., Song, J., Li, X., Liu, Z., Zhang, W., et al. (2019). Population Genomic Analysis and De Novo Assembly Reveal the Origin of Weedy Rice as an Evolutionary Game. Mol. Plant **12**:632–647.

Sun, K., Huang, M., Zong, W., Xiao, D., Lei, C., Luo, Y., Song, Y., Li, S., Hao, Y., Luo, W., et al. (2022). Hd1, Ghd7, and DTH8 synergistically determine the rice heading date and yield-related agronomic traits. Journal of Genetics and Genomics **49**:437–447.

Sweeney, M.T., Thomson, M.J., Pfeil, B.E., and McCouch, S. (2006). Caught Red-Handed: *Rc* Encodes a Basic Helix-Loop-Helix Protein Conditioning Red Pericarp in Rice. Plant Cell **18**:283–294.

Takahashi, Y., Teshima, K.M., Yokoi, S., Innan, H., and Shimamoto, K. (2009). Variations in Hd1 proteins, Hd3a promoters, and Ehd1 expression levels contribute to diversity of flowering time in cultivated rice. Proc. Natl. Acad. Sci. USA **106**:4555–4560.

Terhorst, J., Kamm, J.A., and Song, Y.S. (2017). Robust and scalable inference of population history from hundreds of unphased whole genomes. Nat. Genet. **49**:303–309.

Thorvaldsdóttir, H., Robinson, J.T., and Mesirov, J.P. (2013). Integrative Genomics Viewer (IGV): high-performance genomics data visualization and exploration. Brief. Bioinform. **14**:178–192.

Vicentini, G., Biancucci, M., Mineri, L., Chirivì, D., Giaume, F., Miao, Y., Kyozuka, J., Brambilla, V., Betti, C., and Fornara, F. (2023). Environmental control of rice flowering time. Plant Commun. **4**:100610. https://doi.org/10.1016/j.xplc.2023.100610.

Wang, W., Mauleon, R., Hu, Z., Chebotarov, D., Tai, S., Wu, Z., Li, M., Zheng, T., Fuentes, R.R., Zhang, F., et al. (2018). Genomic variation in 3,010 diverse accessions of Asian cultivated rice. Nature **557**:43–49.

Wang, Q., Su, Q., Nian, J., Zhang, J., Guo, M., Dong, G., Hu, J., Wang, R., Wei, C., Li, G., et al. (2021). The Ghd7 transcription factor represses ARE1 expression to enhance nitrogen utilization and grain yield in rice. Mol. Plant **14**:1012–1023.

Wang, T., He, W., Li, X., Zhang, C., He, H., Yuan, Q., Zhang, B., Zhang, H., Leng, Y., Wei, H., et al. (2023). A rice variation map derived from 10 548 rice accessions reveals the importance of rare variants. Nucleic Acids Res. **51**:10924–10933.

Wei, F.-J., Tsai, Y.-C., Wu, H.-P., Huang, L.-T., Chen, Y.-C., Chen, Y.-F., Wu, C.-C., Tseng, Y.-T., and Hsing, Y.I.C. (2016). Both Hd1 and Ehd1 are important for artificial selection of flowering time in cultivated rice. Plant Sci. **242**:187–194.

Xie, L., Wu, D., Fang, Y., Ye, C., Zhu, Q.-H., Wei, X., and Fan, L. (2024). Population genomic analysis unravels the evolutionary roadmap of pericarp color in rice. Plant Commun. **5**:100778.

Xu, X., Liu, X., Ge, S., Jensen, J.D., Hu, F., Li, X., Dong, Y., Gutenkunst, R.N., Fang, L., Huang, L., et al. (2011). Resequencing 50 accessions of cultivated and wild rice yields markers for identifying agronomically important genes. Nat. Biotechnol. **30**:105–111.

Xue, W., Xing, Y., Weng, X., Zhao, Y., Tang, W., Wang, L., Zhou, H., Yu, S., Xu, C., Li, X., and Zhang, Q. (2008). Natural variation in Ghd7 is an important regulator of heading date and yield potential in rice. Nat. Genet. **40**:761–767.

Yang, J., Lee, S.H., Goddard, M.E., and Visscher, P.M. (2011). GCTA: A Tool for Genome-wide Complex Trait Analysis. Am. J. Hum. Genet. **88**:76–82.

Ye, J., Niu, X., Yang, Y., Wang, S., Xu, Q., Yuan, X., Yu, H., Wang, Y., Wang, S., Feng, Y., and Wei, X. (2018). Divergent Hd1, Ghd7, and DTH7 Alleles Control Heading Date and Yield Potential of Japonica Rice in Northeast China. Front. Plant Sci. **9**:35.

Ye, J., Zhang, M., Yuan, X., Hu, D., Zhang, Y., Xu, S., Li, Z., Li, R., Liu, J., Sun, Y., et al. (2022). Genomic insight into genetic changes and shaping of major inbred rice cultivars in China. New Phytol. **236**:2311–2326.

Zhang, J., Zhou, X., Yan, W., Zhang, Z., Lu, L., Han, Z., Zhao, H., Liu, H., Song, P., Hu, Y., et al. (2015). Combinations of the *Ghd7* , *Ghd8* and *Hd1* genes largely define the ecogeographical adaptation and yield potential of cultivated rice. New Phytol. **208**:1056–1066.

Zhang, J., Jiang, L., Yu, L., Huan, X., Zhou, L., Wang, C., Jin, J., Zuo, X., Wu, N., Zhao, Z., et al. (2024). Rice's trajectory from wild to domesticated in East Asia. Science **384**:901–906.

Zhou, X., Yu, J., Spengler, R.N., Shen, H., Zhao, K., Ge, J., Bao, Y., Liu, J., Yang, Q., Chen, G., et al. (2020). 5,200-year-old cereal grains from the eastern Altai Mountains redate the trans-Eurasian crop exchange. Nat. Plants **6**:78–87.

Zhou, X., Nong, C., Wu, B., Zhou, T., Zhang, B., Liu, X., Gao, G., Mi, J., Zhang, Q., Liu, H., et al. (2021). Combinations of *Ghd7* , *Ghd8* , and *Hd1* determine strong heterosis of commercial rice hybrids in diverse ecological regions. J. Exp. Bot. **72**:6963–6976.

Zong, W., Ren, D., Huang, M., Sun, K., Feng, J., Zhao, J., Xiao, D., Xie, W., Liu, S., Zhang, H., et al. (2021). Strong photoperiod sensitivity is controlled by cooperation and competition among Hd1, Ghd7 and DTH8 in rice heading. New Phytol. **229**:1635–1649.

Plant Communications, Volume 7

Supplemental information

Genomic analyses of weedy rice reveal historical connections between European and northwestern Chinese rice

Yifan Zhong, Wuyue Chen, Shuai Xu, Qijun Zhang, Yixuan Wang, Xiaohui Zhang, Ju Huang, Sihai Yang, and Long Wang

Supplemental Materials

Supplemental Note

Supplemental Note 1 | Evaluating the influence of latitude on allelic effects in flowering-time field trails

Supplemental Note 2 | Assessing the geographic distribution of northwest-derived flowering alleles in public 3K rice genome database

Supplemental Note 3 | Assessing the impact of modern breeding on weedy rice populations

Supplemental Note 4 | Evaluation of the representativeness of weedy rice sampling in northwestern China

Supplemental Figure

Supplemental Figure 1 | Weedy rice phenotypes (awn and seed coat) of partially collected samples.

Supplemental Figure 2 | Phylogenetic tree of 582 rice samples based on genome-wide SNPs with sample IDs.

Supplemental Figure 3 | Primer design and experimental identification of $Hd1^{EH}$ and $Hd4^{NW}$ alleles in rice.

Supplemental Figure 4 | Functional validation of the flowering-time effects of $Hd1^{EH}$ and the deletion-type *Hd4*.

Supplemental Figure 5 | Phylogenetic tree based on SNPs in *Hd1* and its 1k bp promoter region.

Supplemental Figure 6 | Conserved genomic regions between northwestern Chinese weedy rice and European rice identified based on SNP difference ratios.

Supplemental Figure 7 | Geographic distribution of photoperiod genotypes of northwestern origin.

Supplemental Figure 8 | Phylogenies based on SNPs within the gene body and 3-kb upstream and downstream flanking regions of six other photoperiod-related genes.

Supplemental Figure 9 | Schematic diagram of *japonica* weedy rice dispersal and effective

population size history.

Supplemental Figure 10 | Pairwise genetic differentiation (F_{ST}) between the three weedy rice populations.

Supplemental Figure 11 | Minimal impact of modern breeding on local weedy rice populations.

Supplemental Figure 12 | Phylogenetic analysis of the *Rc* gene provides additional evidence for the northwestern Chinese origin of European rice.

Supplemental Figure 13 | Sampling locations of the samples collected from northwestern China in this study.

Supplemental Table

Supplemental Table 1 | IBD segment lengths between Wr_Italy20 and European cultivars

Supplemental Table 2 | IBD segment lengths between European weedy rice individuals and modern European modern cultivars

Supplemental Table 3 | Information of primers in this study.

Supplemental Table 4 | Rice accessions in the 3K rice genome database (3kRG) that carry photoperiod-related alleles found in northwest China.

Supplemental Data

Supplemental Data 1 | List of 23 weedy rice accessions collected and used in this study.

Supplemental Data 2 | List of 559 published rice accessions representing modern cultivars, landraces, and weedy rice.

Supplemental Data 3 | Conserved genomic regions between European rice and northwestern weedy rice.

Supplemental Data 4 | Summary of *Hd1* haplotypes and their distribution across *japonica* rice groups.

Supplemental Data 5 | Summary of *Hd4* haplotypes and their distribution across *japonica* rice groups.

Supplemental Data 6 | Summary of *Hd2* haplotypes and their distribution across *japonica* rice groups.

Supplemental Data 7 | Representative samples used to predict population history.

Supplemental Data 8 | Genotypes of major heading date genes in *japonica* rice samples used in this study.

Supplemental Data 9 | Statistical analyses in this study.

Supplemental references

Supplemental Notes

Supplemental Note 1 | Evaluating the influence of latitude on allelic effects in flowering-time field trails

Because Nanjing is located at a lower latitude than northwestern China and most European rice-growing regions, differences in day-length could influence the absolute heading dates of photoperiod-responsive genes such as *Hd1* and *Hd4*. To assess the robustness of the allelic effects detected in our Nanjing field trials, we further examined multi-site European flowering-time data from Gómez-Ariza et al., (2015), which include five field locations (Italy, France, Greece, Portugal, and Spain) and three years of observations from Italy. For both genes, we restricted comparisons to accessions sharing similar genotypes at other major flowering-time loci to minimize background effects.

For *Hd1*, our Nanjing field trials showed that weedy rice carrying the $Hd1^{EH}$ alleles consistently headed approximately 10.6 days earlier than those carrying the functional $Hd1^{NIP}$ haplotype (Supplemental Figure 4A). Under European field conditions, accessions with matched genotypes at other flowering-time genes (non-functional *Hd5*, functional *Hd2* and *Hd4*) carrying the $Hd1^{EH}$ allele (n = 6) flowered ~6.8 days earlier than those with the functional *Hd1* allele (n = 4) (linear model, $P = 3.591\times 10^{-5}$, Supplemental Figure 4C).

For *Hd4*, our Nanjing trials showed that accessions carrying the $Hd4^{NW}$ alleles flowered approximately 16.0 days earlier than those with the functional $Hd4^{NIP}$ haplotypes (Figure 2G). Consistently, under Europe conditions and matched allelic states at *Hd1* (non-functional), *Hd2* (functional) and *Hd5* (functional), accessions carrying $Hd4^{NW}$ (n = 4) flowered ~9.1 days earlier than those carrying the functional *Hd4* allele (n = 58) (linear model, P = 0.00499, Supplemental Figure 4D).

Collectively, although the absolute magnitudes of flowering-time differences vary between low- and high-latitude environments, likely reflecting photoperiod variation, the direction and relative strength of allelic effects are highly consistent across environments. These results demonstrate that the functional impacts of $Hd1^{EH}$ and $Hd4^{NW}$ are stable across

environments and genetic backgrounds, supporting their roles in photoperiod adaptation in both northwestern Chinese and European rice populations.

Supplemental Note 2 | Assessing the geographic distribution of northwest-derived flowering alleles in public 3K rice genome database

To further evaluate the geographic distribution of key flowering-time alleles, we analyzed variation at the *Hd1*, *Hd2*, and *Hd4* loci using the 3K rice genome database (3kRG). Despite limitations in geographic resolution and the absence of large structural variants, we identified consistent high-latitude enrichment patterns (Supplemental Table 4). The $Hd1^{EH}$ haplotype was detected in 39 accessions, predominantly from Europe, while $Hd2^{NW}$ was found in 23 accessions, over half of which were distributed in Europe and Georgia (Supplemental Table 4). For *Hd4*, 18 accessions carried the inferred $Hd4^{NW}$ deletion haplotype, again largely restricted to high-latitude regions (Supplemental Table 4). Importantly, the full $Hd1^{EH}$-$Hd2^{NW}$-$Hd4^{NW}$ allele combination was detected only in a limited number of accessions from Europe, Georgia, and China, a distribution consistent with historical Silk Road regions (Supplemental Table 4). These results independently reinforce the close genetic relationship between European rice and Chinese rice lineages.

Supplemental Note 3 | Assessing the impact of modern breeding on weedy rice populations

Extensive germplasm exchange between Europe and China during modern rice breeding, together with the dynamic emergence of weedy rice in Europe due to changes in cultivation practices, could potentially confound signals of geographic origin. To evaluate these possibilities, we examined accessions with known histories of germplasm exchange and assessed their influence on weedy rice populations.

First, to evaluate the impact of modern breeding activities, we used the European cultivar "*Balila*", a parental line widely used in breeding programs in northeastern China, as a representative example. In our phylogenetic analyses, *Balila* (Inter_Italy_36) clustered within Clade 1, whereas northeastern cultivars clustered in Clade 3, indicating that its

incorporation into Chinese breeding did not distrupt large-scale phylogenetic structure (Supplemental Figure 2). IBD analyses further showed that *Balila* shares extensive IBD segments with European cultivars but only short tracts with northeastern or northwestern Chinese cultivars (Supplemental Figure 11A). In contrast, weedy rice from all three regions showed only minimal IBD sharing with *Balila* (Supplemental Figure 11A), demonstrating that the phylogenetic clustering of weedy rice remains geographically stable and largely unaffected by modern breeding. Moreover, if intensive modern breeding had obscured ancestral genealogies, modern cultivars would be genetically closer to local weedy rice than landraces. However, in northwest China, weedy rice exhibits lower F_{ST} with local landraces (which experienced minimal modern breeding) but higher F_{ST} with modern cultivars introduced from North China, northeastern China, and Japan (Supplemental Figure 11B). This pattern is inconsistent with extensive recent genetic replacement and argues against the erosion of ancestral signals.

Second, consider the impact of recent cultivar escape, if the European weedy rice had arisen predominantly from recent escapes (post-1980s), they would be expected to cluster closely with cultivars widely planted during that period. To test this, we included representative Italian cultivars spanning the 1980s to the present, such as Sant'Andrea (Inter_Italy_03, planted on 10,823 ha in 1985), Smeraldo (Inter_Italy_15, planted on 4,353 ha in 1985), Rubino (Inter_Italy_25, planted on 913 ha in 1980), Gladio (Inter_Italy_34, developed and planted in the 1990s), Augusto (Inter_Italy_29, still cultivated today), and Loto (Inter_Italy_13, still cultivated today) (Data source: https://openknowledge.fao.org/server/api/core/bitstreams/392ee95b-6852-42cd-a8ec-1a8d8ad6eaf8/content/y4347e0z.htm#TopOfPage). These cultivars are all clustered within Clade 1 (Figure 1C, Supplemental Figure 2). In contrast, most Italian weedy rice samples fall outside this clade (Figure 1C, Supplemental Figure 2), indicating that their de-domestication predates the post-1980s expansion. This finding aligns with prior evidence of continuous historical escape events contributing to European weedy rice populations instead of a recent major outbreak (Grimm et al., 2013).

Together, these results show that neither cultivation practices nor modern varietal turnover has substantially altered the deeper population structure of weedy rice. Ancestral genealogical signals remain intact, supporting the robustness of origin inferences based on genomic data.

Supplemental Note 4 | Evaluation of the representativeness of weedy rice sampling in northwestern China

Genomic inferences are inherently sensitive to sampling design and geographic coverage. To minimize potential bias, our study was intentionally structured to expand sampling in regions historically underrepresented in rice evolutionary studies, particularly northwestern China, which has a long history of rice cultivation but a relatively limited record of modern breeding.

First, earlier investigations into the origin of European rice primarily emphasized northeast China and East Asia, while rice populations from northwest China were largely overlooked. Given the long-standing role of northwest China as a major corridor connecting East and West, we explicitly included both cultivated and weedy rice from this region. Because European rice cultivation is dominated by *japonica* (~90%), we also concentrated on *japonica*-growing regions globally, ensuring balanced representation across relevant ancestral source populations.

Second, sampling bias is more problematic when large areas are sparsely sampled. In contrast, rice cultivation in northwest China is geographically constrained to highly localized and river-valley systems (Ouyang et al., 2023). Our sampling spans all major rice-growing areas across Gansu, Ningxia, and Xinjiang, with substantial geographic distances among sites (Supplemental Figure 13). This spatial coverage greatly reduces the risk that our findings are driven by localized genetic clusters and supports the representativeness of our dataset for the region as a whole.

Third, the extensive arid deserts separating rice-growing regions in northwest China

severely limit natural seed dispersal, making long-distance gene flow nearly entirely human mediated. The high genomic similarity and shared flowering-time allelic combinations observed across weedy rice populations in these isolated regions therefore reflect a common human-driven establishment history rather than recent natural diffusion. Crucially, this same genomic background and characteristic suite of adaptative photoperiod alleles also occurs in European weedy rice and a subset of European cultivars. Such concordance is unlikely to result from local or recent spread alone, and is most parsimoniously explained by a historical westward introduction facilitated by human movement.

Overall, the geographic breadth, ecological distinctiveness, and anthropogenic dispersal context of our northwestern China sampling support its representativeness and reinforce the robustness of our origin inferences.

Supplemental Figure

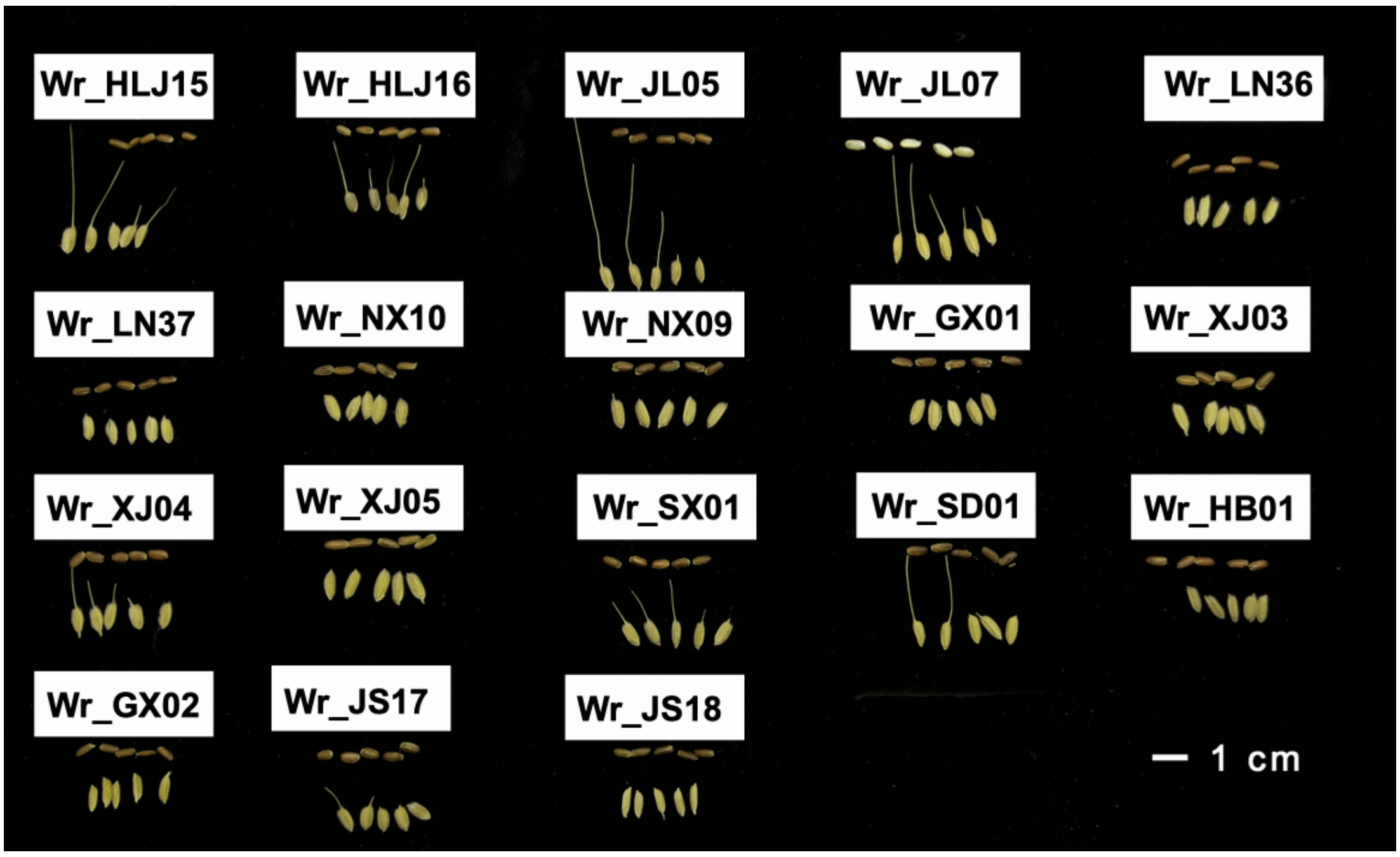

Supplemental Figure 1 | Weedy rice phenotypes (awn and seed coat) of partially collected samples. The white bar represents a length of 1 cm.

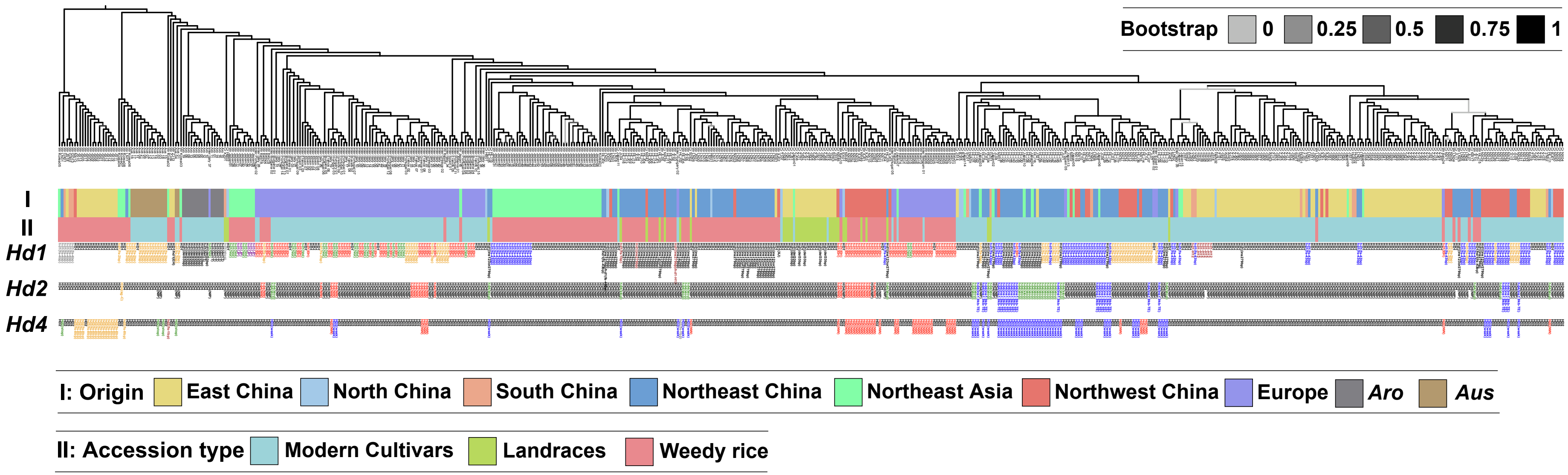

Supplemental Figure 2 | Phylogenetic tree of 582 rice samples based on genome-wide SNPs with sample IDs. Genotypes of *Hd1*, *Hd4*, and *Hd2* are annotated for each sample. The haplotype labels match the numbering used in Supplemental Data 4, Supplemental Data 5 and Supplemental Data 6. Different haplotypes are labeled using different font colors, with NW-originated haplotypes all shown in red. Annotation bars beneath the phylogenetic tree indicate geographic origin (I) and sample classification (II), using the same color scheme as in Figure 1C.

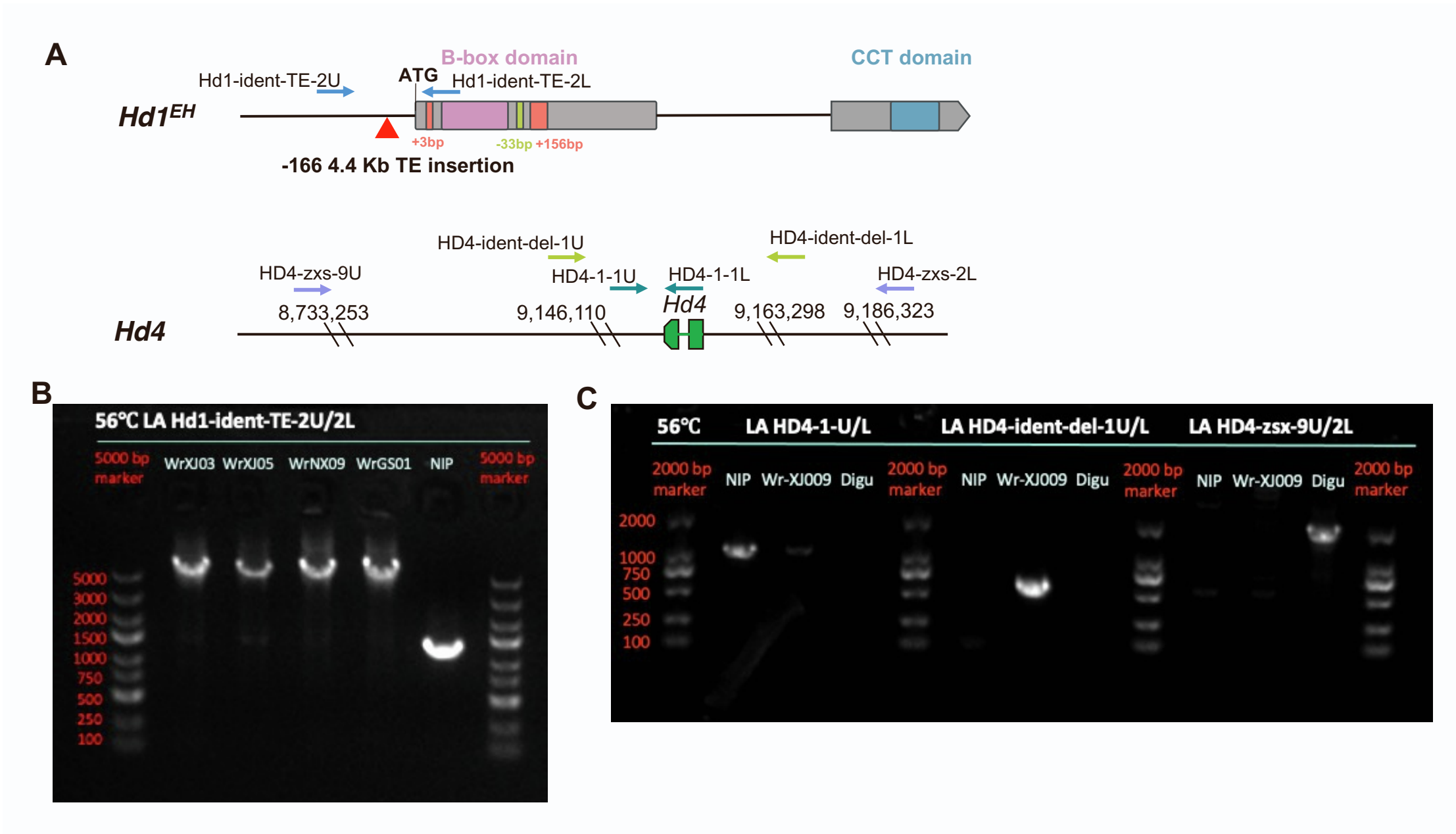

Supplemental Figure 3 | Primer design and experimental identification of *Hd1*^{EH} and *Hd4*^{NW} alleles in rice. (A) Schematic diagram showing the primer design regions used for the identification of *Hd1*^{EH} and *Hd4*^{NW} alleles. (b-c) PCR amplification results for *Hd1*^{EH} and *Hd4*^{NW} alleles. **(B)** Lane 1: DNA marker (size in 5000 bp); Lane 2-4: PCR products for *Hd1*^{NW}, showing a clear band over 5000 bp; Lane 5: PCR products for *Hd1*^{NIP}, showing a clear band at approximately 1380 bp. **(C)** NIP carries an intact Hd4 sequence, while Wr-XJ009 has a 17,187-bp deletion and Digu has a 453,070-bp deletion. Three distinct primer sets were designed to amplify specific bands corresponding to each genotype.

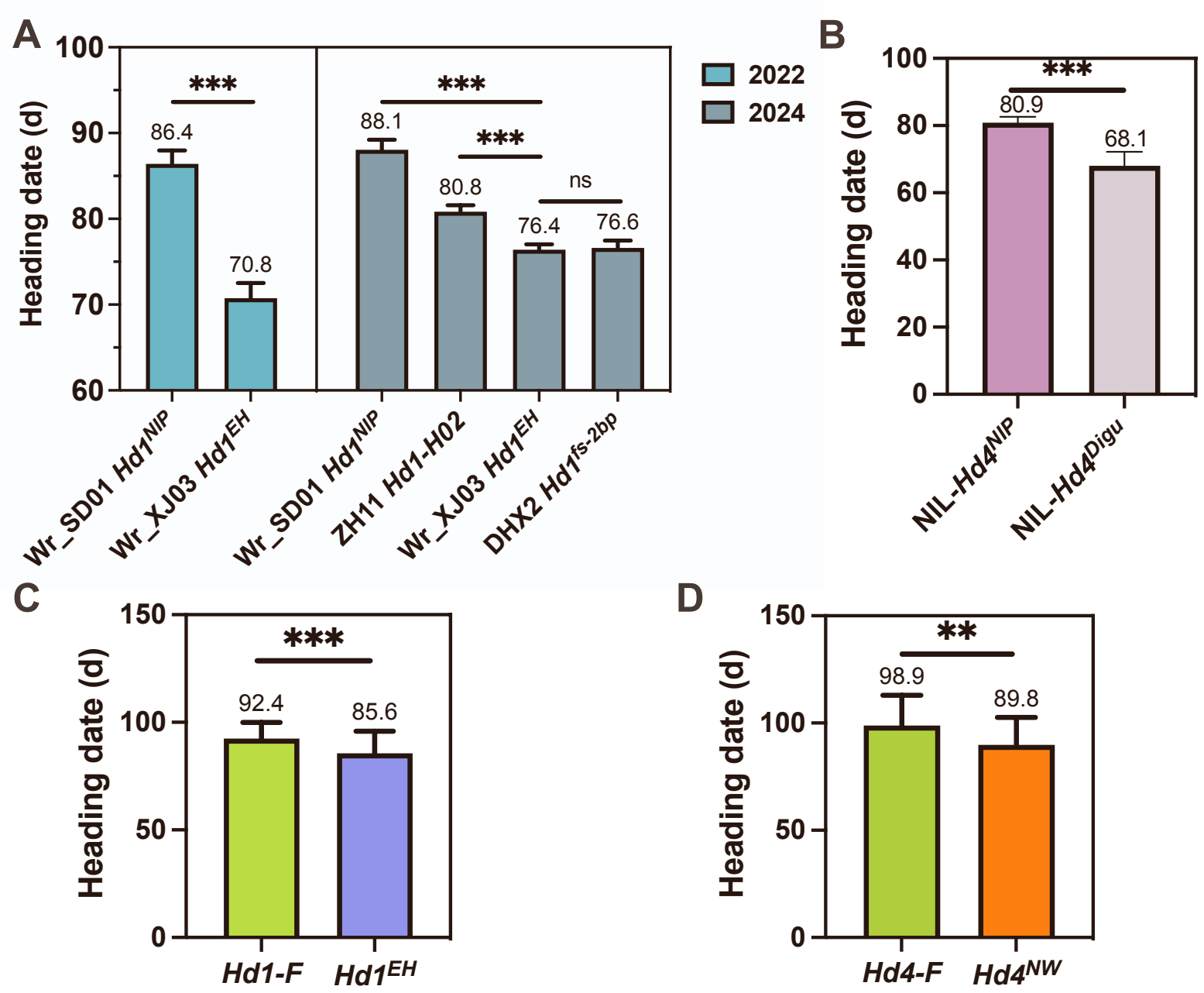

Supplemental Figure 4 | Functional validation of the flowering-time effects of $Hd1^{EH}$ and the deletion-type *Hd4*. (A) Weedy rice carrying $Hd1^{EH}$ flowers earlier than those with functional *Hd1* in a background with functional *Hd2, Hd4* and *Hd5*. Accessions with $Hd1^{EH}$ allele flower significantly earlier than those with functional *Hd1* alleles (Student's t-test, ***P < 0.001, Data S9) but do not differ significantly from the nonfunctional $Hd1^{fs\text{-}2bp}$ (P = 0.462, Data S9). **(B)** Flowering-time variation associated with the deletion-type *Hd4* ($Hd4^{Digu}$) versus functional *Hd4* alleles $Hd4^{NIP}$ in near isogenic lines (NILs). NILs were generated by crossing PA64s (functional $Hd4^{NIP}$) with Zhongjiazao 17 (deletion-type $Hd4^{Digu}$) and advancing progeny to the F_6 generation. From this segregating population, homozygous individuals with a uniform genetic background (functional *Hd1* and *RFT1*, and nonfunctional *Hd2* and *Hd5*) that differed only at the *Hd4* locus were selected. A significant difference in flowering time was observed between the two *Hd4* genotypes (Student's *t*-test, ***P < 0.001, Data S9). **(C)** Flowering-time differences between $Hd1^{EH}$ and functional *Hd1* (*Hd1-F*) alleles in European rice germplasm. Data from Gómez-Ariza et al. (2015); effects were estimated using linear models including genotype, planting site and year as fixed factors (**P < 0.01, Data S9). **(D)** Flowering-time differences between $Hd4^{NW}$ and functional *Hd4* (*Hd4-F*) in European rice germplasm. Data from Gómez-Ariza et al. (2015); effects were estimated using linear models including genotype, planting site and year as fixed factors (**P < 0.01, Data S9).

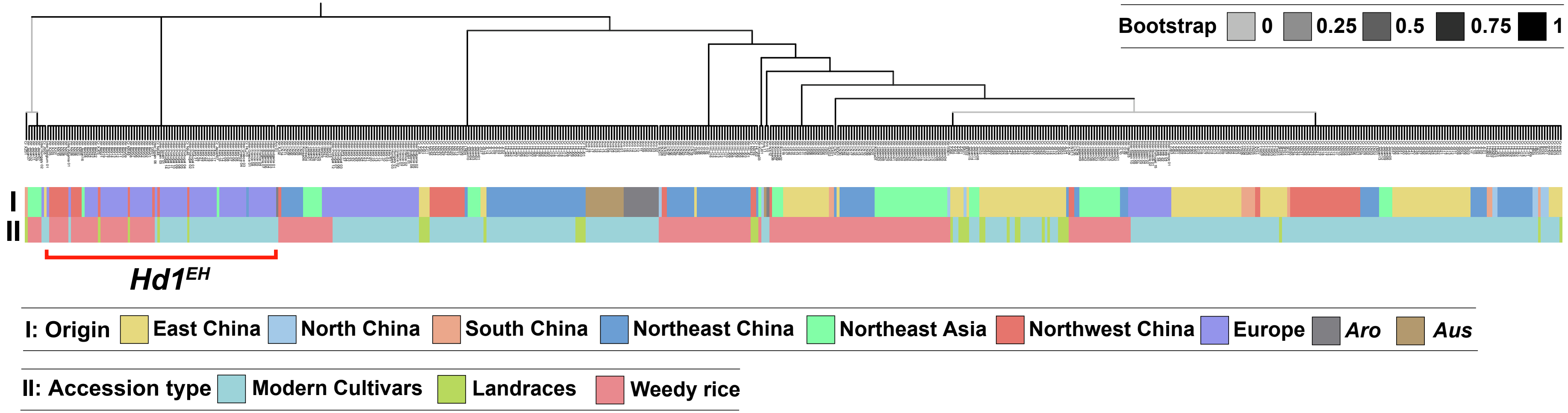

Supplemental Figure 5 | Phylogenetic tree based on SNPs in *Hd1* and its 1k bp promoter region. Individuals with the $Hd1^{EH}$ haplotype are highlighted in red parentheses. Sample IDs are shown beneath the phylogeny. Annotation bars beneath the phylogenetic tree indicate geographic origin (I) and sample classification (II), using the same color scheme as in Figure 1C.

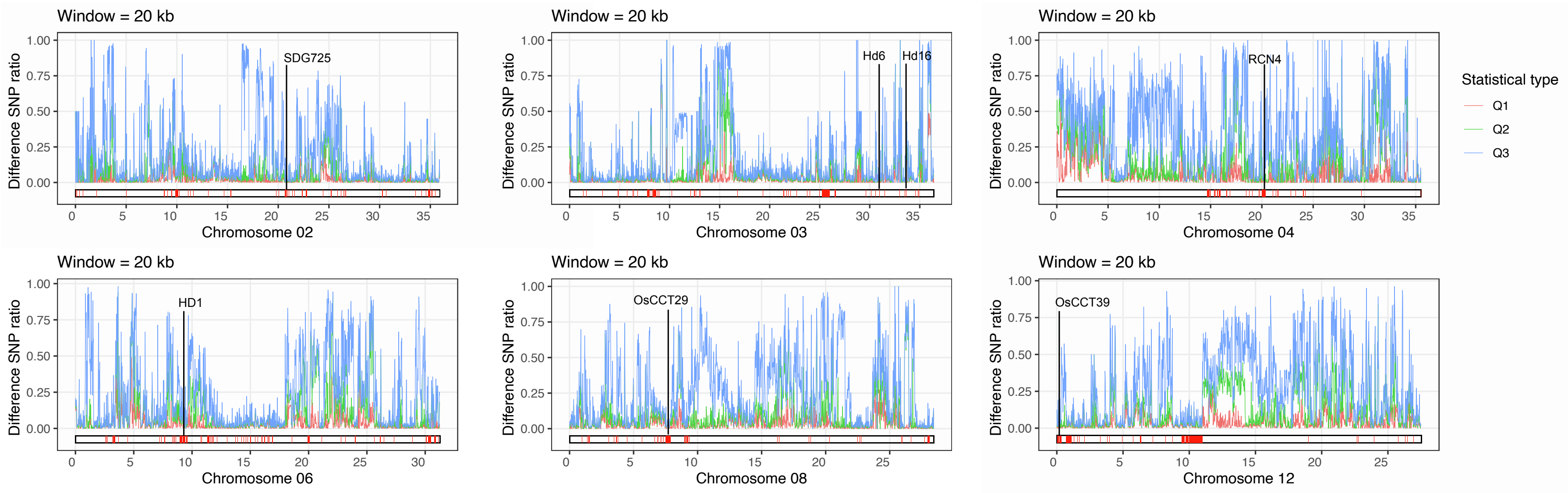

Supplemental Figure 6 | Conserved genomic regions between northwestern Chinese weedy rice and European rice identified based on SNP difference ratios. Red, green, and blue lines indicate the first quartile (Q1), median (Q2), and third quartile (Q3) of the SNP difference distribution, respectively. The red shaded regions at the bottom represent conserved genomic regions where the first quartile of the SNP differential distribution is less than 0.1.

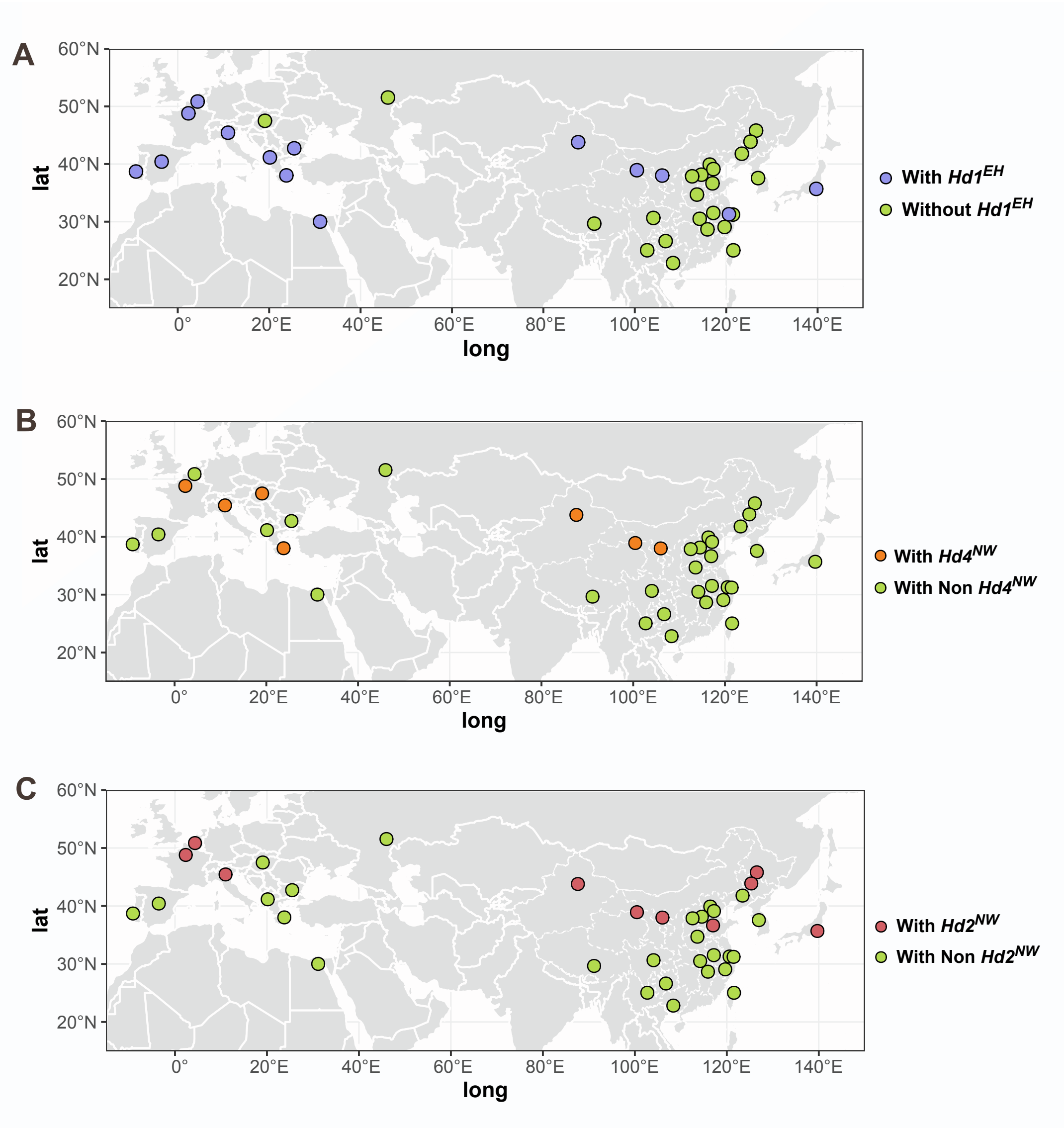

Supplemental Figure 7 | Geographic distribution of photoperiod genotypes of northwestern origin. The geographical distribution of *Hd1*^{EH} **(A)**, *Hd4*^{NW} **(B)** and *Hd2*^{NW} **(C)** in our samples across the Eurasia. Demonstrates a strong correspondence in flowering-time genotypes between northwestern Chinese and European rice populations.

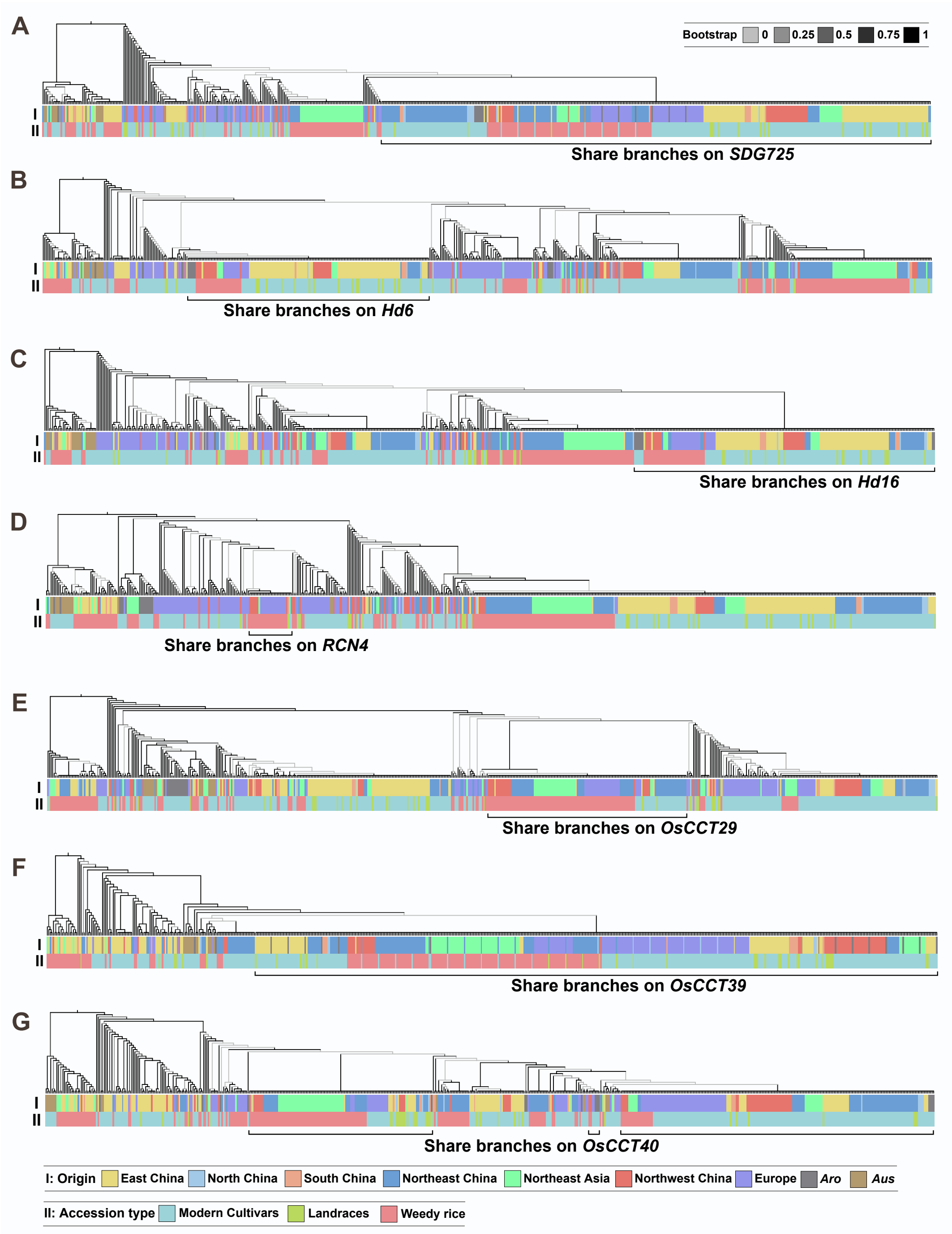

Supplemental Figure 8 | Phylogenies based on SNPs within the gene body and 3-kb upstream and downstream flanking regions of six other photoperiod-related genes. (A) *SDG725*, **(B)** *Hd6*, **(C)** *Hd16*, **(D)** *RCN4*, **(E)** *OsCCT29*, **(F)** *OsCCT39*, **(G)** *OsCCT40*. A subset of European cultivars and European weedy rice consistently cluster with northwestern Chinese weedy rice, and are highlighted with a black arc in the phylogenetic tree. Annotation bars beneath the phylogenetic tree indicate geographic origin (I) and sample classification (II), using the same color scheme as in Figure 1C.

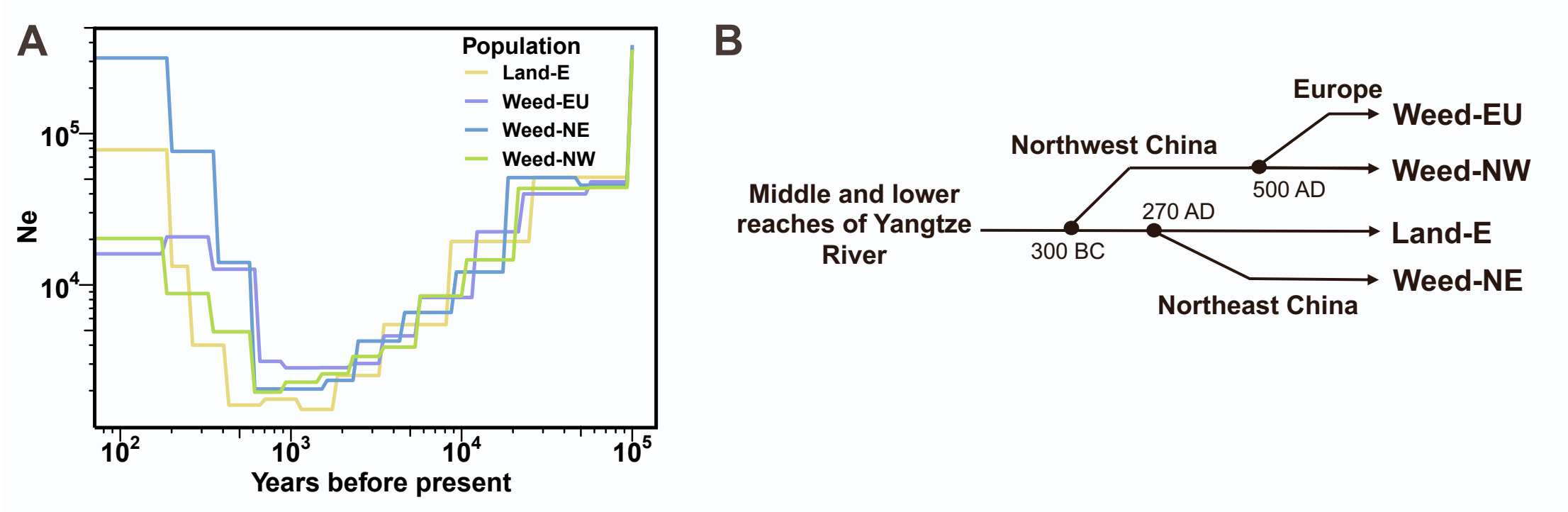

Supplemental Figure 9 | Schematic diagram of *japonica* weedy rice dispersal and effective population size history. (A) Effective population history predicted using the single population method. **(B)** Schematic diagram of the inferred dispersal timeline of *japonica* weedy rice based on effective population size history. Land-E, East Chinese landraces; Weed-NW, northwestern Chinese weedy rice; Weed-NE, northeastern Chinese weedy rice; Weed-EU, European weedy rice.

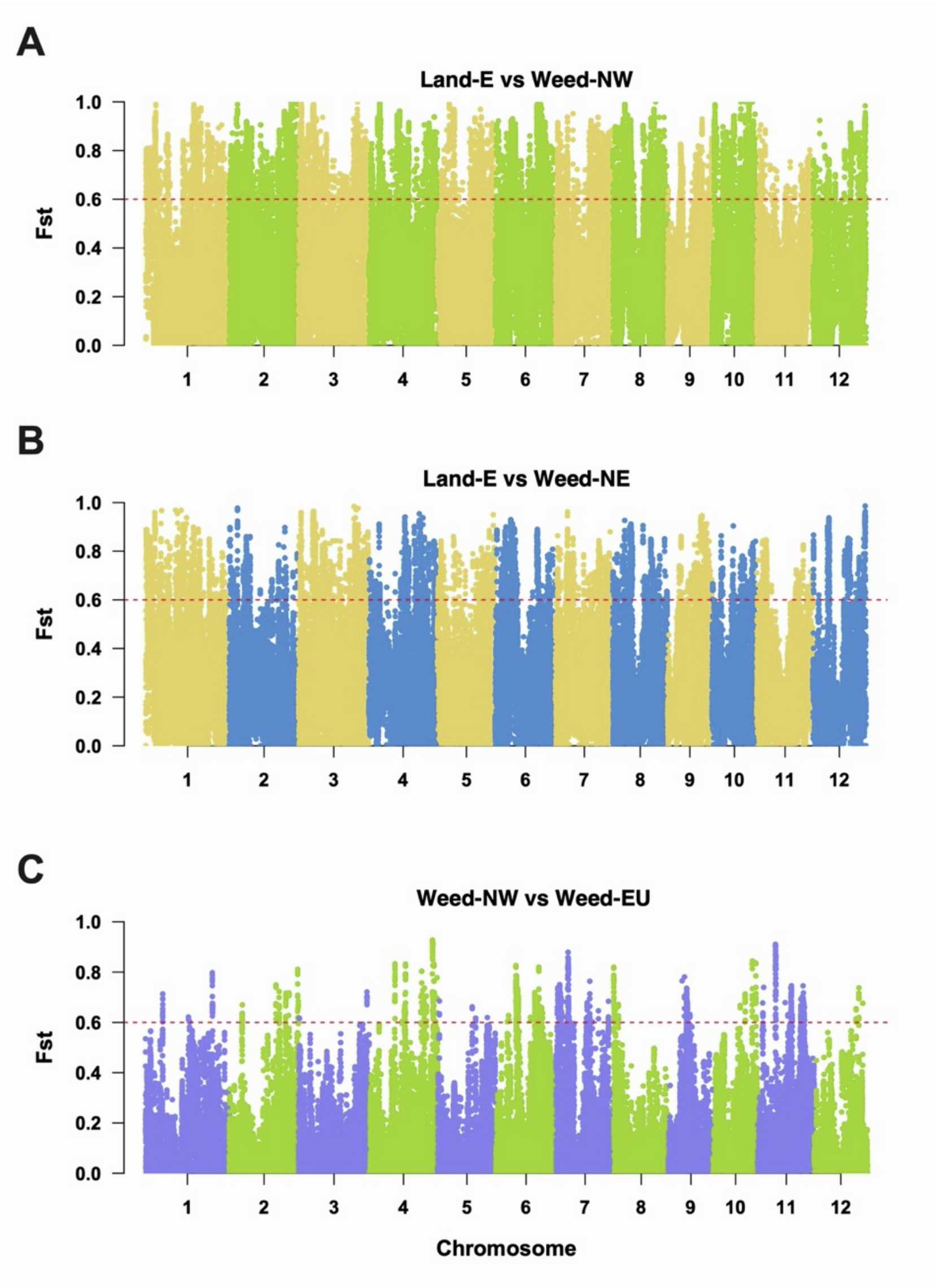

Supplemental Figure 10 | Pairwise genetic differentiation (F_{ST}) between the three weedy rice populations. **(A)** East Chinese landraces versus northwestern Chinese weedy rice; **(B)** East Chinese landraces versus northeastern Chinese weedy rice; (C) northwestern Chinese weedy rice versus European weedy rice. Land-E, East Chinese landraces; Weed-NW, northwestern Chinese weedy rice; Weed-NE, northeastern Chinese weedy rice; Weed-EU, European weedy rice.

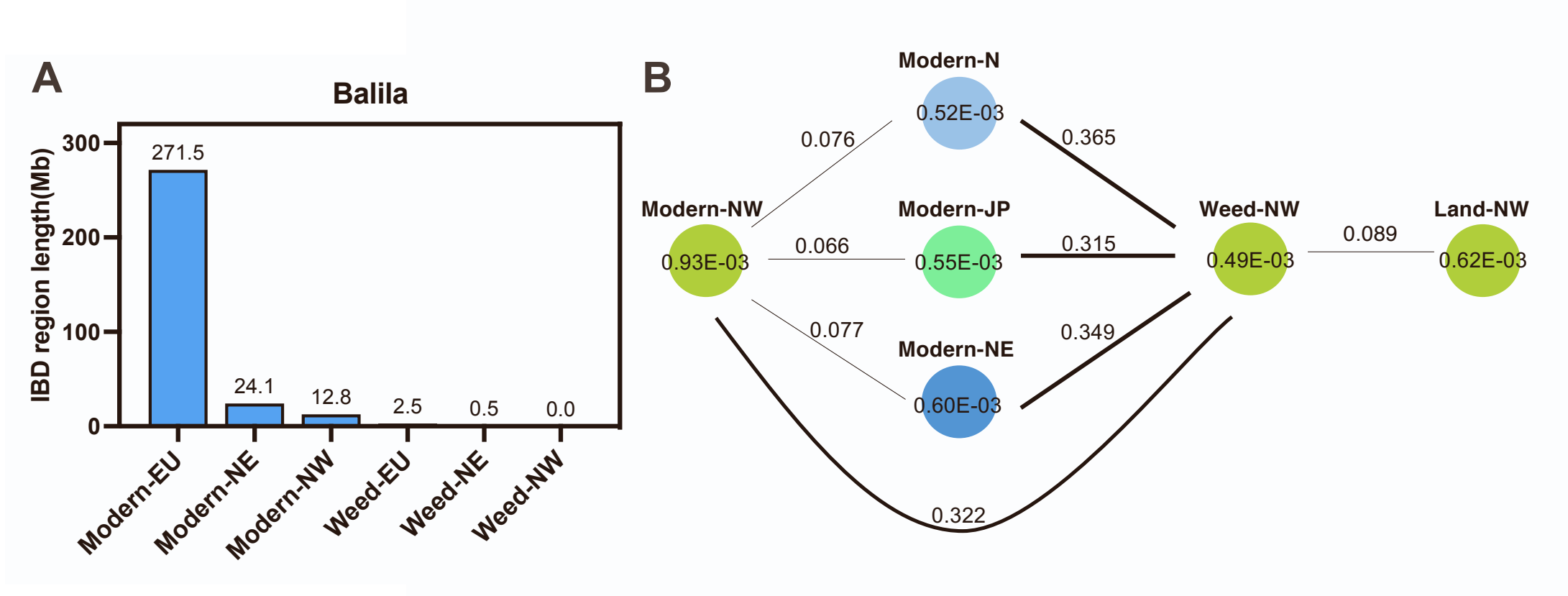

Supplemental Figure 11 | Minimal impact of modern breeding on local weedy rice populations. (A) Total length of IBD segments shared between the European cultivar *Balila* and European, northeastern, and northwestern weedy rice and modern cultivars. Numbers on the bars indicate cumulative IBD segment lengths. **(B)** Nucleotide diversity (π) and population differentiation (F_{ST}) between northwestern weedy rice, modern cultivars from various regions, and local northwestern landraces. Circle values indicate π for each population; numbers on connecting lines indicate F_{ST}. Thinner lines represent lower F_{ST} values. Modern-EU, European modern cultivars; Modern NE, northeastern Chinese modern cultivars; Modern-NW, northwestern Chinese modern cultivars; Weed-EU, European weedy rice; Weed-NE, northeastern Chinese weedy rice; Weed-NW, northwestern Chinese weedy rice; Land-NW, northwestern Chinese landraces; Modern-JP, Japanese modern cultivars.

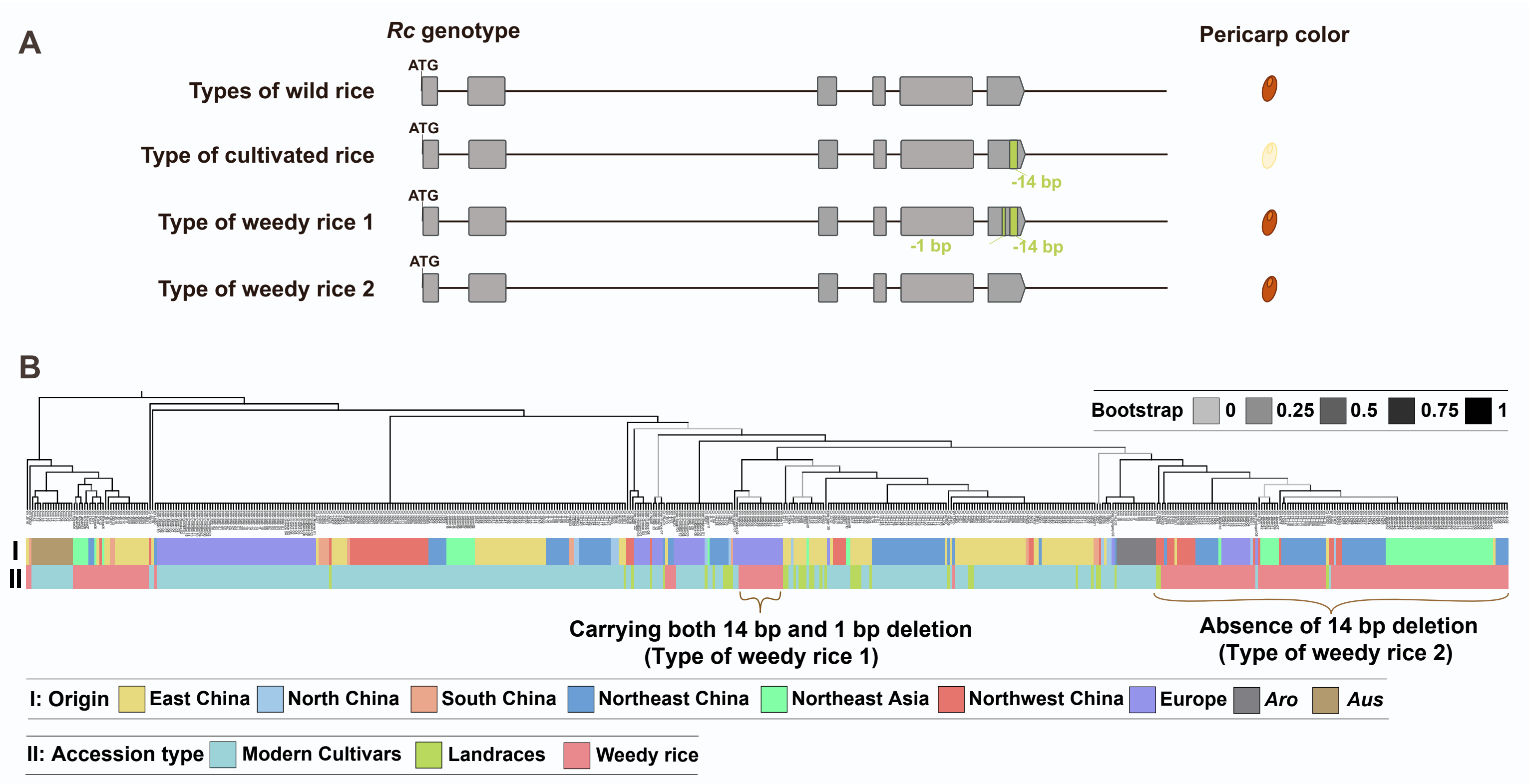

Supplemental Figure 12 | Phylogenetic analysis of the *Rc* gene provides additional evidence for the northwestern Chinese origin of European rice. (A) Representative *Rc* genotypes and corresponding pericarp color phenotypes in wild rice, cultivars, and weedy rice populations. **(B)** Phylogenetic tree constructed using SNPs from the *Rc* gene and its 3-kb upstream and downstream region, with sample IDs labeled. The two genotypes associated with red pericarp in weedy rice are marked in parentheses. Annotation bars beneath the phylogenetic tree indicate geographic origin (I) and sample classification (II), using the same color scheme as in Figure 1C.

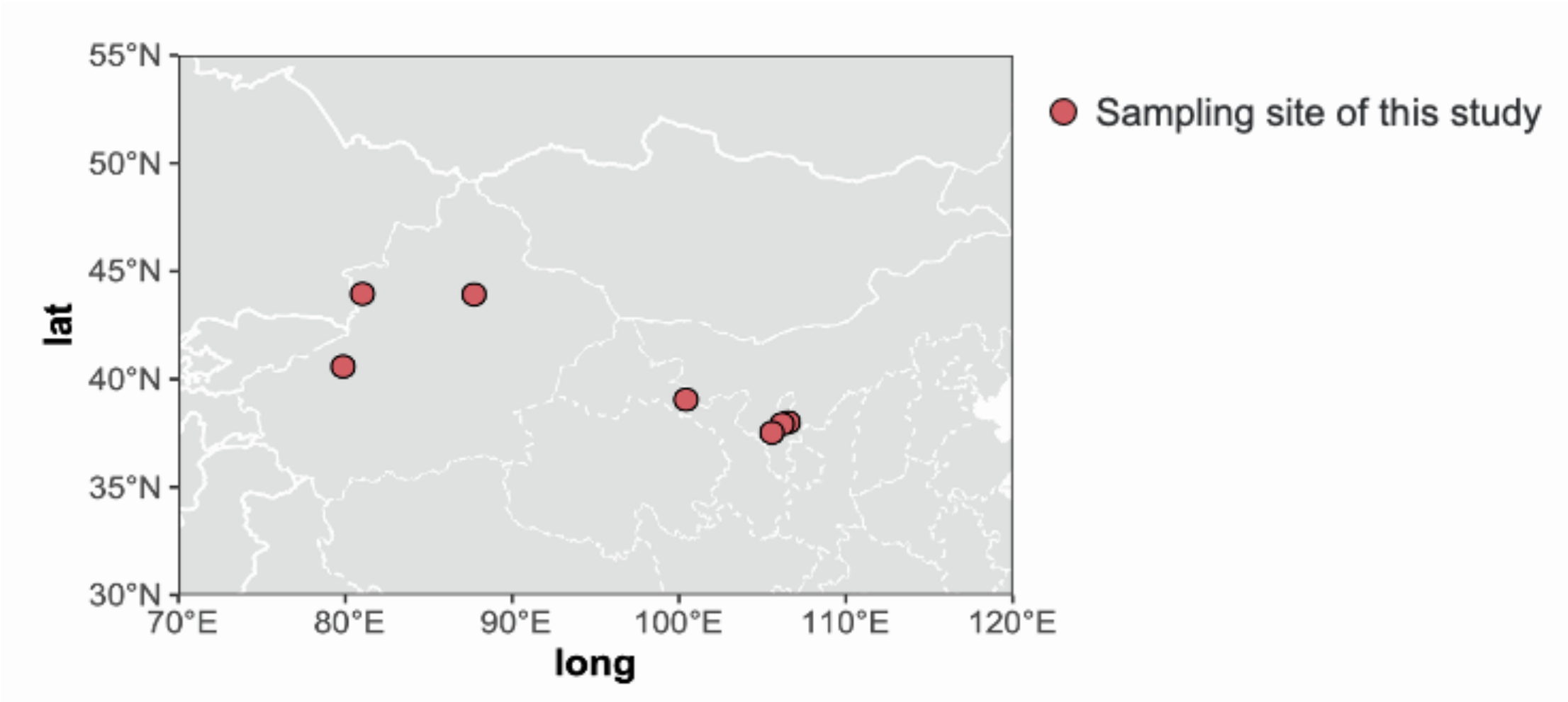

Supplemental Figure 13 | Sampling locations of the samples collected from northwestern China in this study.

Supplemental Table

Supplemental Table 1 | IBD segment lengths between Wr_Italy20 and European cultivars

Sample of European cultivars	IBD size (Mb)
Inter_Albania_01	0
Inter_Bulgaria_01	0
Inter_Bulgaria_02	0
Inter_Bulgaria_03	0.541288
Inter_Bulgaria_04	0
Inter_Bulgaria_05	0
Inter_Bulgaria_06	0
Inter_Bulgaria_07	0
Inter_Bulgaria_08	3.074247
Inter_Egypt_01	0
Inter_France_01	0
Inter_France_02	6.779831
Inter_France_03	0
Inter_France_04	0.890408
Inter_France_05	0
Inter_France_06	0
Inter_France_07	19.130954
Inter_France_08	14.591105
Inter_Greece_01	18.818832
Inter_Greece_02	69.635791
Inter_Greece_03	11.88922
Inter_Greece_04	0
Inter_Hungary_01	0
Inter_Hungary_03	0
Inter_Italy_01	0.78987
Inter_Italy_02	0
Inter_Italy_03	1.184416
Inter_Italy_04	0.702362
Inter_Italy_05	0

Inter_Italy_06	19.220945
Inter_Italy_07	1.626646
Inter_Italy_08	0.941679
Inter_Italy_09	0
Inter_Italy_10	0
Inter_Italy_11	5.636904
Inter_Italy_12	16.600106
Inter_Italy_13	0.354499
Inter_Italy_14	0
Inter_Italy_15	0
Inter_Italy_16	5.607676
Inter_Italy_17	2.053776
Inter_Italy_18	0
Inter_Italy_19	0
Inter_Italy_20	42.871146
Inter_Italy_21	5.596049
Inter_Italy_22	12.847456
Inter_Italy_23	23.386632
Inter_Italy_24	1.108355
Inter_Italy_25	0
Inter_Italy_26	71.973949
Inter_Italy_27	1.812559
Inter_Italy_28	0.468216
Inter_Italy_29	1.038515
Inter_Italy_30	2.188311
Inter_Italy_31	0
Inter_Italy_32	0
Inter_Italy_33	0.67916
Inter_Italy_34	146.413128
Inter_Italy_36	0
Inter_Italy_38	0
Inter_Portugal_01	0.971997
Inter_Portugal_02	6.073548
Inter_Portugal_03	1.037989
Inter_Portugal_04	0
Inter_Portugal_06	0

Inter_Portugal_07	0
Inter_Portugal_08	0
Inter_Portugal_09	0
Inter_Portugal_10	0.633855
Inter_Portugal_11	0
Inter_Portugal_12	0
Inter_Portugal_13	0
Inter_Portugal_14	0
Inter_Portugal_15	0
Inter_Russia_01	0
Inter_Russia_02	0
Inter_Spain_01	0
Inter_Spain_02	0
Inter_Spain_03	0
Inter_Spain_04	0
Inter_Spain_06	0
Inter_Spain_07	0
Inter_Spain_08	3.938009
Inter_Spain_09	1.373842
Inter_Spain_10	0

Supplemental Table 2 | IBD segment lengths between European weedy rice individuals and modern European modern cultivars

Sample	IBD share with CrEU (Mb)
Wr_Italy01	17.042525
Wr_Italy02	26.321698
Wr_Italy03	18.691196
Wr_Italy04	47.85537
Wr_Italy05	25.000292
Wr_Italy06	24.98659
Wr_Italy07	19.318802
Wr_Italy08	37.580425
Wr_Italy09	26.628139
Wr_Italy10	20.475191
Wr_Italy11	35.912636
Wr_Italy12	32.941376
Wr_Italy13	21.57655
Wr_Italy14	82.2356
Wr_Italy15	33.869091
Wr_Italy16	33.221424
Wr_Italy17	25.449308
Wr_Italy18	30.809438
Wr_Italy19	86.264013
Wr_Italy20	236.358609
Wr_Italy21	28.728231
Wr_Italy22	76.575907
Wr_Italy23	37.741635
Wr_Italy24	12.969275
Wr_Italy25	47.700568
Wr_Italy26	29.641857
Wr_Italy27	107.904443
Wr_Italy28	12.723567
Wr_Italy29	55.72497

Supplemental Table 3 | Information of primers in this study.

Primer name	Primer sequence	Purpose
Hd1-ident-TE-2U	ACAAACTCAATTCATCAACACGA	Identification of transposon Insertion in the *Hd1*^{EH}
Hd1-ident-TE-2L	TTTAGCTTGCACCACATGGAC	
Hd4-ident-del-1U	TTGCGGCTATAAAGATTTCCC	Identification of the *Hd4*^{NW} deletion type
Hd4-ident-del-1L	AGGAAGTATCCACAACGCAAG	
HD4-1-1U	GCGAAGCGACCTCTCACT	Identification of the *Hd4*^{NIP} functional type
HD4-1-1L	GTGCTTCTCGGTCTATTTACTT	
ZSX-9U	GCCGAAGATGACTTTATTTT	Identification of the *Hd4*^{Digu} deletion type
ZSX-2L	TGGGACCGCTTCAGTACTA	

Supplemental Table 4 | Rice accessions in the 3K rice genome database (3kRG) that carry photoperiod-related alleles found in northwest China. Accessions in bold represent those with the full *Hd1*^{EH}–*Hd2*^{NW}–*Hd4*^{NW} allele combination.

Haplotype	Ratio in 3kRG	Original area and variety name
Hd1^{EH}	39/1436	Argentina(**PRECOZ 2 F A**), Bulgaria(KALIN, PLOVDIV 22, SAEDINENIE), Chile(BULI INIA), China(AI YEH LU, **SHA TIAO TSAO**), Colombia(CT 23, CT 36, CT 58), France(CALENDAL, CIGALON, **MEJANES 2**), Georgia(**WIR 1951**), Greece(MELAS, ROXANI), Hungary(**SZANISZLO 2**), India(T 757), Italy(GIOVANNI MARCHETTI, GITANO, GRITNA, LUXOR, PIEMONTE, REA, SALVO), Philippines(FIDJI, KASIGNAYAN, PARAY QIKUG KABAYUH), Portugal(CAMPINO, ESCARLATE, FAISCA, HAREM, IBO 400, OTA, S 102/2, SAGRES, TOPAZIO), Red River Delta(DELTA), United States(DACCA6)
Hd2^{NW}	23/3015	Argentina(NANOAY P.A, 104 UPLA, **PRECOZ 2 F A**), Belgium(WIR 1072), Bulgaria(AERJITUO), China(HEIMANGDAO, **SHA TIAO TSAO**, LONGHUAMAOHU, MONGOLIA RICE), France(**MEJANES 2**, KELUODUO B), Georgia(**WIR 1951**, WIR 2091), Greece(REA), Hungary(PI 282203, KOPANCSI KEREK, **SZANISZLO 2**, AOMIERTE 168), Italy(GHIBLI, MIARA), Norway(WIR 884), Philippines(FIDJI), United States of America(M 102)
Hd4^{NW}	18/3024	Argentina(**PRECOZ 2 F A**), China(**SHA TIAO TSAO**, HEIMANGDAO), France(**MEJANES 2**, KELUODUO B), Georgia(**WIR 1951**, WIR 2091), Hungary(PI 282203, KOPANCSI KEREK, **SZANISZLO 2**, H 305-84, AUZGUSTA), Italy(SALVO, OPALE, GHIBLI, MIARA, DORELLA), Norway(WIR 884)

Supplemental Data

Supplemental Data 1 | List of 23 weedy rice accessions collected and used in this study. (Separate Excel File)

Supplemental Data 2 | List of 559 published rice accessions representing modern cultivars, landraces, and weedy rice. (Separate Excel File)

Supplemental Data 3 | Conserved genomic regions between European rice and northwestern weedy rice. (Separate Excel File)

Supplemental Data 4 | Summary of *Hd1* haplotypes and their distribution across *japonica* rice groups. (Separate Excel File)

Supplemental Data 5 | Summary of *Hd4* haplotypes and their distribution across *japonica* rice groups. (Separate Excel File)

Supplemental Data 6 | Summary of *Hd2* haplotypes and their distribution across *japonica* rice groups. (Separate Excel File)

Supplemental Data 7 | Representative samples used to predict population history. (Separate Excel File)

Supplemental Data 8 | Genotypes of major heading date genes in *japonica* rice samples used in this study. (Separate Excel File)

Supplemental Data 9 | Statistical analyses in this study. (Separate Excel File)

Supplemental references

Gómez-Ariza, J., Galbiati, F., Goretti, D., Brambilla, V., Shrestha, R., Pappolla, A., Courtois, B., and Fornara, F. (2015). Loss of floral repressor function adapts rice to higher latitudes in Europe. *Journal of Experimental Botany* **66**:2027–2039.

Grimm, A., Fogliatto, S., Nick, P., Ferrero, A., and Vidotto, F. (2013). Microsatellite markers reveal multiple origins for Italian weedy rice Advance Access published 2013.

Ouyang, Z., Jackson, R. B., McNicol, G., Fluet-Chouinard, E., Runkle, B. R. K., Papale, D., Knox, S. H., Cooley, S., Delwiche, K. B., Feron, S., et al. (2023). Paddy rice methane emissions across Monsoon Asia. *Remote Sensing of Environment* **284**:113335.
